# Changing life expectancy in European countries 1990–2021: a subanalysis of causes and risk factors from the Global Burden of Disease Study 2021

**DOI:** 10.1016/S2468-2667(25)00009-X

**Published:** 2025-02-18

**Authors:** GBD 2021 Europe Life Expectancy Collaborators, Nicholas Steel, Clarissa Maria Mercedes Bauer-Staeb, John A Ford, Cristiana Abbafati, Mohammed Altigani Abdalla, Atef Abdelkader, Parsa Abdi, Roberto Ariel Abeldaño Zuñiga, Olugbenga Olusola Abiodun, Hassan Abolhassani, Eman Abu-Gharbieh, Hana J Abukhadijah, Ahmed Abu-Zaid, Isaac Yeboah Addo, Giovanni Addolorato, Victor Adekanmbi, Juliana Bunmi Adetunji, Temitayo Esther Adeyeoluwa, Emilie E Agardh, Williams Agyemang-Duah, Danish Ahmad, Anisuddin Ahmed, Ayman Ahmed, Syed Anees Ahmed, Karolina Akinosoglou, Mohammed Ahmed Akkaif, Salah Al Awaidy, Syed Mahfuz Al Hasan, Omar Ali Mohammed Al Zaabi, Robert W Aldridge, Abdelazeem M Algammal, Adel Ali Saeed Al-Gheethi, Abid Ali, Mohammed Usman Ali, Syed Shujait Ali, Waad Ali, Gianfranco Alicandro, Sheikh Mohammad Alif, Adel Al-Jumaily, Peter Allebeck, Ahmad Alrawashdeh, Rami H Al-Rifai, Mohammed A Alsabri, Najim Z Alshahrani, Deborah Oyine Aluh, Mohammad Al-Wardat, Walid A Al-Zyoud, Sohrab Amiri, Deanna Anderlini, Catalina Liliana Andrei, Abhishek Anil, Saeid Anvari, Anayochukwu Edward Anyasodor, Seth Christopher Yaw Appiah, Michele Aquilano, Jalal Arabloo, Mosab Arafat, Demelash Areda, Abdulfatai Aremu, Keivan Armani, Benedetta Armocida, Johan Ärnlöv, Muhammad Asaduzzaman, Thomas Astell-Burt, Avinash Aujayeb, Marcel Ausloos, Sina Azadnajafabad, Shahkaar Aziz, Ahmed Y Azzam, Giridhara Rathnaiah Babu, Andreea Corina Badache, Ashish D Badiye, Saeed Bahramian, Atif Amin Baig, Jennifer L Baker, Hansi Bansal, Till Winfried Bärnighausen, Mark Thomaz Ugliara Barone, Amadou Barrow, Sandra Barteit, Shahid Bashir, Hameed Akande Bashiru, João Diogo Basso, Mohammad-Mahdi Bastan, Sanjay Basu, Kavita Batra, Matteo Bauckneht, Bernhard T Baune, Massimiliano Beghi, Maryam Beiranvand, Yannick Béjot, Michelle L Bell, Olorunjuwon Omolaja Bello, Luis Belo, Apostolos Beloukas, Alice A Beneke, Paulo J G Bettencourt, Akshaya Srikanth Bhagavathula, Neeraj Bhala, Sonu Bhaskar, Francesca Bisulli, Tone Bjørge, Aadam Olalekan Bodunrin, Alejandro Botero Carvajal, Souad Bouaoud, Carol Brayne, Hermann Brenner, Adam D M Briggs, Nikolay Ivanovich Briko, Raffaele Bugiardini, Danilo Buonsenso, Reinhard Busse, Yasser Bustanji, Florentino Luciano Caetano dos Santos, Mehtap Çakmak Barsbay, Angelo Capodici, Giulia Carreras, Andrea Carugno, Felix Carvalho, Márcia Carvalho, Joao Mauricio Castaldelli-Maia, Giulio Castelpietra, Alberico L Catapano, Maria Sofia Cattaruzza, Luca Cegolon, Edina Cenko, Ester Cerin, Sonia Cerrai, Anis Ahmad Chaudhary, Bryan Chong, Sonali Gajanan Choudhari, Dinh-Toi Chu, Isaac Sunday Chukwu, Sheng-Chia Chung, Iolanda Cioffi, Joao Conde, Samuele Cortese, Rosa A S Couto, Michael H Criqui, Natalia Cruz-Martins, Omid Dadras, Mary Anne Teresa Dallat, Emanuele D'Amico, Lucio D'Anna, Samuel Demissie Darcho, Paul I Dargan, Saswati Das, Alejandro de la Torre-Luque, Cristian Del Bo', Andreas K Demetriades, Nikolaos Dervenis, Brecht Devleesschauwer, Arkadeep Dhali, Kuldeep Dhama, Mostafa Dianatinasab, Michael J Diaz, Deepa Dongarwar, Mario D'Oria, Ojas Prakashbhai Doshi, Robert Kokou Dowou, Senbagam Duraisamy, Oyewole Christopher Durojaiye, Arkadiusz Marian Dziedzic, David Edvardsson, Kristina Edvardsson, Terje Andreas Eikemo, Michael Ekholuenetale, Temitope Cyrus Ekundayo, Rabie Adel El Arab, Frank J Elgar, Muhammed Elhadi, Chadi Eltaha, Francesco Esposito, Natalia Fabin, Adeniyi Francis Fagbamigbe, Omotayo Francis Fagbule, Aliasghar Fakhri-Demeshghieh, Luca Falzone, Carla Sofia e Sá Farinha, Pawan Sirwan Faris, Folorunso Oludayo Fasina, Patrick Fazeli, Timur Fazylov, Alireza Feizkhah, Ginenus Fekadu, Xiaoqi Feng, Seyed-Mohammad Fereshtehnejad, Daniela Ferrante, Pietro Ferrara, Nuno Ferreira, Getahun Fetensa, Florian Fischer, Marco Fonzo, Arianna Fornari, Daniela Fortuna, Celia Fortuna Rodrigues, Matteo Foschi, Sebastian S Fox, Alberto Freitas, Takeshi Fukumoto, Muktar A Gadanya, Silvano Gallus, Lucia Galluzzo, Balasankar Ganesan, Mohammad Arfat Ganiyani, Xiang Gao, MA Garcia-Gordillo, Federica Gazzelloni, Miglas Welay Gebregergis, Teferi Gebru Gebremeskel, Delaram J Ghadimi, Khalid Yaser Ghailan, Nermin Ghith, Ehsan Gholami, Alessandro Gialluisi, Paramjit Singh Gill, Tara Gillam, Giorgia Giussani, James C Glasbey, Scott D Glenn, Laszlo Göbölös, Mohamad Goldust, Mahaveer Golechha, Pouya Goleij, Davide Golinelli, Giuseppe Gorini, Simon Matthew Graham, Robert Griebler, Ashna Grover, Stefano Guicciardi, Sasidhar Gunturu, Vijai Kumar Gupta, Roberth Steven Gutiérrez-Murillo, Awoke Derbie Habteyohannes, Nils Haep, Nguyen Hai Nam, Sebastian Haller, Rifat Hamoudi, Senad Handanagic, Josep Maria Haro, Hamidreza Hasani, Md Saquib Hasnain, Rasmus J Havmoeller, Simon I Hay, Jeffrey J Hebert, Behzad Heibati, Henk B M Hilderink, Yuta Hiraike, Nguyen Quoc Hoan, Mehdi Hosseinzadeh, Sorin Hostiuc, Hanno Hoven, Chengxi Hu, Junjie Huang, Andrew Hughes, Michael Hultström, Kiavash Hushmandi, Javid Hussain, M Azhar Hussain, Adalia Ikiroma, Arit Inok, Md Rabiul Islam, Sheikh Mohammed Shariful Islam, Gaetano Isola, Mahalaxmi Iyer, Louis Jacob, Haitham Jahrami, Ammar Abdulrahman Jairoun, Sanobar Jaka, Mihajlo Jakovljevic, Talha Jawaid, Bijay Mukesh Jeswani, Jost B Jonas, Charity Ehimwenma Joshua, Billingsley Kaambwa, Zubair Kabir, Dler H Hussein Kadir, Rajesh Kamath, Kehinde Kazeem Kanmodi, Neeti Kapoor, Paschalis Karakasis, Marina Karanikolos, Ibraheem M Karaye, Joonas H Kauppila, Sina Kazemian, Emmanuelle Kesse-Guyot, Faham Khamesipour, Ajmal Khan, Shaghayegh Khanmohammadi, Khaled Khatab, Moawiah Mohammad Khatatbeh, Moein Khormali, Atulya Aman Khosla, Majid Khosravi, Mahmood Khosrowjerdi, Jagdish Khubchandani, Kwanghyun Kim, Min Seo Kim, Adnan Kisa, Sezer Kisa, Ann Kristin Skrindo Knudsen, Gerbrand Koren, Md Abdul Kuddus, Ilari Kuitunen, Mukhtar Kulimbet, Rakesh Kumar, Setor K Kunutsor, Om P Kurmi, Dian Kusuma, Ville Kytö, Carlo La Vecchia, Hanpeng Lai, Tea Lallukka, Francesco Lanfranchi, Berthold Langguth, Ariane Laplante-Lévesque, Heidi Jane Larson, Anders O Larsson, Munjae Lee, Paul H Lee, Seung Won Lee, Wei-Chen Lee, Daniel Lindholm, Christine Linehan, Xuefeng Liu, Erand Llanaj, José Francisco López-Gil, Stefan Lorkowski, Giancarlo Lucchetti, Alessandra Lugo, Raimundas Lunevicius, Lisha Luo, Hawraz Ibrahim M Amin, Zheng Feei Ma, Nikolaos Machairas, Monika Machoy, Kashish Malhotra, Ahmad Azam Malik, Ali Mansour, Emmanuel Manu, Hamid Reza Marateb, Daniela Martini, Miquel Martorell, Roy Rillera Marzo, Yasith Mathangasinghe, Medha Mathur, Fernanda Penido Matozinhos, Richard James Maude, Andrea Maugeri, Juergen May, Mahsa Mayeli, Mohsen Mazidi, Martin McKee, Enkeleint A Mechili, Sepideh Mehravar, Tesfahun Mekene Meto, Hadush Negash Meles, Alexios-Fotios A Mentis, Atte Meretoja, Tuomo J Meretoja, Sachith Mettananda, Georgia Micha, Irmina Maria Michalek, Ted R Miller, Giuseppe Minervini, Antonio Mirijello, Gabriele Mocciaro, Atousa Moghadam Fard, Jama Mohamed, Nouh Saad Mohamed, Abdollah Mohammadian-Hafshejani, Shafiu Mohammed, Lorenzo Monasta, Stefania Mondello, Mohammad Ali Moni, Paula Moraga, Lidia Morawska, Tilahun Belete Mossie, Rohith Motappa, Sumaira Mubarik, Lorenzo Muccioli, Ulrich Otto Mueller, Faraz Mughal, Francesk Mulita, Daniel Munblit, Yanjinlkham Munkhsaikhan, Christopher J L Murray, Mohsen Naghavi, Pirouz Naghavi, Ganesh R Naik, Soroush Najdaghi, Atta Abbas Naqvi, Delaram Narimani Davani, Gustavo G Nascimento, Abdallah Y Naser, Abdulqadir J Nashwan, Javaid Nauman, Samidi Nirasha Kumari Navaratna, Athare Nazri-Panjaki, Chakib Nejjari, Evangelia Nena, Henok Biresaw Netsere, Anh Hoang Nguyen, Phat Tuan Nguyen, Van Thanh Nguyen, Lawrence Achilles Nnyanzi, Syed Toukir Ahmed Noor, Mehran Nouri, Fred Nugen, Mario Cesare Nurchis, Ogochukwu Janet Nzoputam, Bogdan Oancea, Martin James O'Donnell, Michael Safo Oduro, Oluwaseun Adeolu Ogundijo, Ropo Ebenezer Ogunsakin, Sylvester Reuben Okeke, Osaretin Christabel Okonji, Andrew T Olagunju, Susan Oliver, Isaac Iyinoluwa Olufadewa, Alberto Ortiz, Mayowa O Owolabi, Mahesh Padukudru P A, Jagadish Rao Padubidri, Raul Felipe Felipe Palma-Alvarez, Sujogya Kumar Panda, Songhomitra Panda-Jonas, Georgios D Panos, Leonidas D Panos, Ioannis Pantazopoulos, Shahina Pardhan, Romil R Parikh, Roberto Passera, Shankargouda Patil, Dimitrios Patoulias, Shrikant Pawar, Umberto Pensato, Gavin Pereira, Norberto Perico, Simone Perna, Fanny Emily Petermann-Rocha, Hoang Nhat Pham, Anil K Philip, Daniela Pierannunzio, Manon Pigeolet, Enrico Pisoni, Dimitri Poddighe, Ramesh Poluru, Maarten J Postma, Jalandhar Pradhan, Elisabetta Pupillo, Jagadeesh Puvvula, Alberto Raggi, Mosiur Rahman, Muhammad Aziz Rahman, Diego Raimondo, Ivano Raimondo, Shakthi Kumaran Ramasamy, Sheena Ramazanu, Rishabh Kumar Rana, Sowmya J Rao, Davide Rasella, Ahmed Mustafa Rashid, Santosh Kumar Rauniyar, Ilari Rautalin, David Laith Rawaf, Salman Rawaf, Murali Mohan Rama Krishna Reddy, Elrashdy M Moustafa Mohamed Redwan, Lennart Reifels, Giuseppe Remuzzi, Mohsen Rezaeian, Ana Isabel Ribeiro, Anupa Rijal, Jefferson Antonio Buendia Rodriguez, Michele Romoli, Luca Ronfani, Kevin T Root, Himanshu Sekhar Rout, Nitai Roy, Michele Russo, Aly M A Saad, Cameron John Sabet, Mamta Sachdeva Dhingra, Umar Saeed, Mehdi Safari, Mahdi Safdarian, Mohamed A Saleh, Mohammed Z Y Salem, Giovanni A Salum, Vijaya Paul Samuel, Abdallah M Samy, Aswini Saravanan, Babak Saravi, Chinmoy Sarkar, Jennifer Saulam, Nikolaos Scarmeas, Benedikt Michael Schaarschmidt, Christophe Schinckus, Markus P Schlaich, Jurgen Carlo Schmidt, Art Schuermans, Austin E Schumacher, Falk Schwendicke, Catherine Schwinger, Sadaf G Sepanlou, Mahan Shafie, Hamid R Shahsavari, Masood Ali Shaikh, Husain Shakil, Sunder Sham, Muhammad Aaqib Shamim, Nigussie Tadesse Sharew, Amin Sharifan, Amin Shavandi, Rekha Raghuveer Shenoy, Mahabalesh Shetty, Pavanchand H Shetty, Premalatha K Shetty, Mika Shigematsu, Aminu Shittu, Ivy Shiue, Seyed Afshin Shorofi, Rajan Shrestha, Roman Shrestha, Emmanuel Edwar Siddig, João Pedro Silva, Luís Manuel Lopes Rodrigues Silva, Soraia Silva, Puneetpal Singh, Surjit Singh, Jussi O T Sipilä, Anna Aleksandrovna Skryabina, Anton Sokhan, Soroush Soraneh, Joan B Soriano, Ireneous N Soyiri, Michael Spartalis, Paschalis Steiropoulos, Leo Stockfelt, Jing Sun, Johan Sundström, David Sunkersing, Katharina S Sunnerhagen, Chandan Kumar Swain, Lukasz Szarpak, Sree Sudha T Y, Payam Tabaee Damavandi, Rafael Tabarés-Seisdedos, Seyyed Mohammad Tabatabaei, Celine Tabche, Ramin Tabibi, Jabeen Taiba, Manoj Tanwar, Nathan Y Tat, Nuno Taveira, Mohamad-Hani Temsah, Rasiah Thayakaran, Tenaw Yimer Tiruye, Mathilde Touvier, Marcos Roberto Tovani-Palone, Jasmine T Tran, Ngoc Ha Tran, Thang Huu Tran, Domenico Trico, Samuel Joseph Tromans, Evangelia Eirini Tsermpini, Lorainne Tudor Car, Munkhtuya Tumurkhuu, Saeed Ullah, Brigid Unim, Asokan Govindaraj Vaithinathan, Mario Valenti, Jef Van den Eynde, Orsolya Varga, Tommi Juhani Vasankari, Balachandar Vellingiri, Massimiliano Veroux, Dominique Vervoort, Jorge Hugo Villafañe, Francesco S Violante, Giuseppe Vizzielli, Alice Vodden, Stein Emil Vollset, Theo Vos, Hatem A Wafa, Yanzhong Wang, Emebet Gashaw Wassie, Kosala Gayan Weerakoon, Ronny Westerman, Nuwan Darshana Wickramasinghe, Peter Willeit, Marcin W Wojewodzic, Axel Walter Wolf, Charles D A Wolfe, Grant M A Wyper, Xiaoyue Xu, Yuichi Yasufuku, Sanni Yaya, Saber Yezli, Arzu YiÄŸit, Dong Keon Yon, Chuanhua Yu, Fathiah Zakham, Aurora Zanghì, Michael Zastrozhin, Mohammed G M Zeariya, Liqun Zhang, Zhiqiang Zhang, Claire Chenwen Zhong, Bin Zhu, Makan Ziafati, Magdalena Zielińska, Elric Zweck, Sa'ed H Zyoud, John N Newton

## Abstract

**Background:**

Decades of steady improvements in life expectancy in Europe slowed down from around 2011, well before the COVID-19 pandemic, for reasons which remain disputed. We aimed to assess how changes in risk factors and cause-specific death rates in different European countries related to changes in life expectancy in those countries before and during the COVID-19 pandemic.

**Methods:**

We used data and methods from the Global Burden of Diseases, Injuries, and Risk Factors Study 2021 to compare changes in life expectancy at birth, causes of death, and population exposure to risk factors in 16 European Economic Area countries (Austria, Belgium, Denmark, Finland, France, Germany, Greece, Iceland, Ireland, Italy, Luxembourg, the Netherlands, Norway, Portugal, Spain, and Sweden) and the four UK nations (England, Northern Ireland, Scotland, and Wales) for three time periods: 1990–2011, 2011–19, and 2019–21. Changes in life expectancy and causes of death were estimated with an established life expectancy cause-specific decomposition method, and compared with summary exposure values of risk factors for the major causes of death influencing life expectancy.

**Findings:**

All countries showed mean annual improvements in life expectancy in both 1990–2011 (overall mean 0·23 years [95% uncertainty interval [UI] 0·23 to 0·24]) and 2011–19 (overall mean 0·15 years [0·13 to 0·16]). The rate of improvement was lower in 2011–19 than in 1990–2011 in all countries except for Norway, where the mean annual increase in life expectancy rose from 0·21 years (95% UI 0·20 to 0·22) in 1990–2011 to 0·23 years (0·21 to 0·26) in 2011–19 (difference of 0·03 years). In other countries, the difference in mean annual improvement between these periods ranged from –0·01 years in Iceland (0·19 years [95% UI 0·16 to 0·21] *vs* 0·18 years [0·09 to 0·26]), to –0·18 years in England (0·25 years [0·24 to 0·25] *vs* 0·07 years [0·06 to 0·08]). In 2019–21, there was an overall decrease in mean annual life expectancy across all countries (overall mean –0·18 years [95% UI –0·22 to –0·13]), with all countries having an absolute fall in life expectancy except for Ireland, Iceland, Sweden, Norway, and Denmark, which showed marginal improvement in life expectancy, and Belgium, which showed no change in life expectancy. Across countries, the causes of death responsible for the largest improvements in life expectancy from 1990 to 2011 were cardiovascular diseases and neoplasms. Deaths from cardiovascular diseases were the primary driver of reductions in life expectancy improvements during 2011–19, and deaths from respiratory infections and other COVID-19 pandemic-related outcomes were responsible for the decreases in life expectancy during 2019–21. Deaths from cardiovascular diseases and neoplasms in 2019 were attributable to high systolic blood pressure, dietary risks, tobacco smoke, high LDL cholesterol, high BMI, occupational risks, high alcohol use, and other risks including low physical activity. Exposure to these major risk factors differed by country, with trends of increasing exposure to high BMI and decreasing exposure to tobacco smoke observed in all countries during 1990–2021.

**Interpretation:**

The countries that best maintained improvements in life expectancy after 2011 (Norway, Iceland, Belgium, Denmark, and Sweden) did so through better maintenance of reductions in mortality from cardiovascular diseases and neoplasms, underpinned by decreased exposures to major risks, possibly mitigated by government policies. The continued improvements in life expectancy in five countries during 2019–21 indicate that these countries were better prepared to withstand the COVID-19 pandemic. By contrast, countries with the greatest slowdown in life expectancy improvements after 2011 went on to have some of the largest decreases in life expectancy in 2019–21. These findings suggest that government policies that improve population health also build resilience to future shocks. Such policies include reducing population exposure to major upstream risks for cardiovascular diseases and neoplasms, such as harmful diets and low physical activity, tackling the commercial determinants of poor health, and ensuring access to affordable health services.

**Funding:**

Gates Foundation.

**Research in context**

**Evidence before this study**

In 2018, the Organisation for Economic Co-operation and Development reported on the slowdown in improvements in life expectancy in many European countries since 2011, and called for further analysis to better understand the relative contributions of different factors. Since then, the high mortality during the COVID-19 pandemic led to decreases in life expectancy in many, but not all, European countries. The Global Burden of Diseases, Injuries, and Risk Factors Study (GBD) 2021 estimated life expectancy, causes of death, and associated risk factors from 1990 to 2021. GBD methods facilitate international comparisons and analysis of the relative contributions of different risk exposure levels to changes in life expectancy before and during the COVID-19 pandemic. In Europe, the 16 founding European Economic Area (EEA) countries and the four nations of the UK are reasonably similar in terms of their economies and geographical locations, yet have different government policies that influence population exposure to major risk factors that likely have a knock-on effect on life expectancy.

**Added value of this study**

This study compared changes in life expectancy, disaggregated by causes of death, and changes in population exposure to attributable risk factors for the major causes of death, in the 16 founding EEA countries and four UK nations over three time periods: 1990 to 2011 (pre-slowdown in life expectancy), 2011 to 2019 (slowdown in life expectancy to pre-COVID-19 pandemic), and 2019 to 2021 (COVID-19 pandemic). We found that from 1990 to 2011, reductions in deaths from cardiovascular diseases and cancers led to substantial improvements in life expectancy in all the studied countries. From 2011 to 2019, life expectancy improvement slowed with marked international differences. The countries that best maintained improvements in life expectancy after 2011 (Norway, Iceland, Sweden, Denmark, and Belgium) had no decrease in life expectancy from 2019 to 2021, despite the COVID-19 pandemic. Exposure to some of the major attributable risks for cardiovascular diseases and cancers, such as high BMI, high systolic blood pressure, and high LDL cholesterol, increased or stopped improving in many or all countries after 2011.

**Implications of all the available evidence**

The extent to which life expectancy slowed during 2011–19 was largely determined by changes in mortality from cardiovascular diseases and cancers. Countries with maintained improvements in mortality from these conditions went on to maintain increases in life expectancy during the COVID-19 pandemic. The slowdown in life expectancy in other countries suggests that improved treatment for individuals with raised lipids or blood pressure is not sufficient to offset the effect of adverse population changes, for example in BMI, or sustained high exposure to dietary risks. Different national policies were potentially associated with the changes in risk factors and mortality patterns that were observed. Our findings suggest that stronger government policies are needed to reduce population levels of major attributable risks including high BMI, dietary risks, and upstream factors such as low physical activity and the wider commercial and social determinants of health, to improve population health over the long term and build resilience to future shocks.

## Introduction

Life expectancy is an important summary measure of the health of populations and has been increasing in high-income countries since at least 1900, interrupted only by periods of high mortality during both world wars and the 1918 influenza pandemic.^1^ The increase has been due to sustained and progressive improvements in infant mortality, nutrition, living standards, and the control of major infectious diseases such as tuberculosis and cholera.^2^, ^3^ In recent decades, increases in life expectancy among high-income countries have been due to reducing death rates from non-communicable diseases, especially cardiovascular diseases and some cancers, with reductions in risk factors such as smoking and raised blood pressure.^3^ The rise in life expectancy has slowed down since 2011,^3^, ^4^ and further slowed in many countries when the COVID-19 pandemic occurred in 2020. The COVID-19 pandemic itself led to exceptionally high mortality rates and corresponding decreases in life expectancy due to COVID-19 in many countries. These falls in life expectancy are not yet recovering as consistently as they did in 2014–15 after a severe influenza season, and there remains substantial heterogeneity across countries with some locations continuing to have substantial excess mortality post-2021.^5^, ^6^ There could still be a continuing impact on life expectancy due to the COVID-19 pandemic, for example from continued disruption to health services as a result of work postponed during the pandemic, and from post-COVID-19 condition and effects on multiple organ systems.^7^

Recovery from the sustained slowdown in improvements in life expectancy from around 2011 could be especially difficult if the underlying causes remain poorly understood. The Organisation for Economic Co-operation and Development (OECD) has reported on potential explanations for the slowdown in life expectancy. The OECD highlighted changes in direct causes of death, including smaller reductions in deaths from cardiovascular diseases and some cancers, and increased respiratory deaths in older people in some winters, since 2011.^3^ Meanwhile, a rise in age-specific death rates due to dementia is at least partly due to changes in coding practices over time.^8^ The OECD report considered potential effects of changes in underlying risk factors, including the well established links between the rise in obesity and diabetes and raised mortality from cardiovascular diseases, widening socioeconomic inequalities in mortality, and economic downturns and austerity.

Multiple causes are likely to be responsible for the observed trends in life expectancy and researchers have previously called for further analysis to improve understanding of the relative contributions of different factors.^3^, ^9^ One difficulty with such international research is that nationally produced mortality statistics are often not comparable between countries due to methodological differences. The long-standing Global Burden of Diseases, Injuries, and Risk Factors Study (GBD) has provided comprehensive assessments of global health for three decades and goes to great lengths to achieve internationally comparable estimates of mortality, life expectancy, morbidity, and associated risk factors, and is therefore well suited to analysis of the causes of international trends in life expectancy.^10^, ^11^, ^12^

In this analysis, we compared trends in life expectancy, causes of death, and risk factors estimated by GBD 2021 for the 16 founding European Economic Area (EEA) countries and the four nations of the UK from 1990 to 2021. We built on the published GBD 2021 capstone papers on global causes of death, life expectancy, risk factors, and disease forecasting.^10^, ^11^, ^12^, ^13^ We aimed to identify trends in specific causes of death and risk factors associated with changing life expectancy in specific countries and for the EEA and the UK. This manuscript was produced as part of the GBD Collaborator Network and in accordance with the GBD Protocol.

## Methods

### Overview

Life expectancy at birth, overall and with decomposition by cause of death, summary exposure values (SEVs) for risk factors, and deaths attributable to specific risk factors were estimated from 1990 to 2021 for the 16 EEA countries and the four UK nations that were part of the EEA at its inception. These countries were chosen because they are reasonably similar in terms of their economies and geographical locations. The countries were Austria, Belgium, Denmark, Finland, France, Germany, Greece, Iceland, Ireland, Italy, Luxembourg, the Netherlands, Norway, Portugal, Spain, Sweden, and the UK nations of England, Northern Ireland, Scotland, and Wales. We compared three time periods: 1990 to 2011, 2011 to 2019 and 2019 to 2021. 2011 and 2019 were included at both the start and end of the time periods so as not to exclude changes from 2010 to 2011 and from 2019 to 2020. 2021 is the most recent GBD data year, 2019 was the last year before the COVID-19 pandemic was declared in 2020, and 2011 was when we identified a statistically significant change in the slope of life expectancy improvement for the region from 1990 to 2019 (see next section). The mean annual changes in life expectancy during these intervals were estimated, with 95% uncertainty intervals (UIs) for each year's individual life expectancy. Life expectancy at birth is the mean number of years that a newborn infant could expect to live, if he or she were to pass through life exposed to the sex-specific and age-specific death rates prevailing at the time of his or her birth, in a given country. Countries are presented according to 2019 life expectancy as 2019 was the last year before the COVID-19 pandemic. Population sizes in 2021 are also presented, sourced from the GBD Results tool.

### Change in life expectancy improvement

The year when there was an overall slowdown in life expectancy improvement was estimated by joinpoint regression modelling, described in detail previously.^14^ A multisegmented line was fitted with use of the population-weighted means of all selected countries' life expectancy at birth from 1990 to 2019, in order to find an approximate point where a change in trend occurred (pooled life expectancy values for the combined group of countries were not available). Joinpoint regression identifies a statistically significant change in trend for time-series data. It assumes that a single linear model does not fully capture a trend but rather that data can be sectioned, with each section having a unique trend. To identify one predominant joinpoint, and avoid capturing small fluctuations in trend, the number of joinpoints was restricted to one. Joinpoint regression was implemented in the National Cancer Institute Joinpoint Regression Program (version 4.9.0.0).^15^

### Life expectancy decomposition

Life expectancy was calculated using age-specific mortality rates with data from vital statistics registers, surveys and censuses, and standard demographic methods as described previously.^10^ The method for estimation of 95% UIs is presented in appendix 1 (p 6). Cause-specific death rates for 288 causes of death, organised in hierarchical levels (levels 1–4) of increasing granularity for causes of death, were estimated with the Cause of Death Ensemble model, a modelling tool developed for GBD to produce stable estimates of mortality across age, location, year, and sex, adjusted to match the total number of all-cause deaths, as previously described.^12^ Imprecise causes of deaths were redistributed to the most likely alternative causes of death.^12^

Changes in life expectancy were attributed to changes in causes of death (at GBD level 2, in which causes are grouped into 22 clusters) for each period, in order to identify the contribution of changes in specific causes of death to the slowdown of improvement in life expectancy. Life expectancy decomposition by cause was used to quantify contributions from specific causes of death by country with an established decomposition method.^12^ Firstly, age-specific life expectancy was calculated, and secondly, the top 20 GBD causes of death that contributed to the variation in life expectancy within each age group were identified. Finally, these cause-age-specific contributions were aggregated across age groups to produce cause-specific contributions to the overall change in life expectancy over a given period. Life expectancy decomposition data for the total years within each period were explored as well as annualised data (estimates divided by the total number of years in each period) to facilitate comparisons between intervals with differing lengths of time. One of the causes identified was other COVID-19 pandemic-related outcomes; the process for estimating mortality from other COVID-19 pandemic-related outcomes has been described previously.^6^, ^10^ Briefly, this mortality estimate is the difference between excess mortality due to the COVID-19 pandemic and the sum of deaths due directly to COVID-19 infection and indirect deaths due to lower respiratory infections, measles, and pertussis. The top five causes were presented, with remaining causes grouped into “other” causes. Respiratory infections and tuberculosis were included as a cause across all time periods regardless of whether a top five cause to allow for comparisons before and during the COVID-19 pandemic.

### Risk factor estimation

GBD 2021 produced epidemiological estimates for 88 risk factors (organised into four hierarchical levels of increasing granularity, with 20 risks at level 2) and their associated health outcomes for a total of 631 risk–outcome pairs.^11^ The methods used to synthesise large amounts of heterogeneous data for risk–outcome pairs have been described previously.^11^ The relative risks of each outcome occurring as a result of exposure to each risk were estimated for each risk–outcome pair. A new method in GBD 2021 is the burden of proof approach to evaluating the strength of evidence of risk–outcome relationships by combining effect size and consistency of evidence.^11^, ^16^ SEVs and theoretical minimum risk exposure levels were estimated for each risk factor, as previously described.^11^ SEV is the GBD measure of risk-weighted exposure prevalence, which indicates a population's exposure to a risk factor accounting for the extent of exposure by risk level and the severity of that risk's contribution to disease burden. The SEV is on a 0–100 scale where 100 means the entire population is at maximum risk and 0 means the population is at minimum risk. The theoretical minimum risk exposure level is the minimum theoretically possible level of risk in the exposed population.

The crude mean age-standardised death rates attributable to major risk factors for all countries combined in 2019 were estimated for each of cardiovascular diseases and neoplasms, as the causes of death responsible for the largest improvements in life expectancy up to 2019, as a prespecified part of the analysis. The top 10 level 2 risk factors, plus low physical activity if not included in the top 10 as an upstream risk factor for both cardiovascular diseases and neoplasms (within the top 11 risk factors for both causes), were presented. Changes in SEVs for risk factors for cardiovascular diseases and neoplasms over time from 1990 to 2021 were plotted by country. Additionally, the crude mean annual rates of change in SEVs for risk factors for cardiovascular diseases and neoplasms for each country were plotted against the crude mean annual rates of change for life expectancy for 1990–2011 and 2011–19, to show any associations. The visual display of information on multiple risk factors was limited to the top five risk factors (based on death rates attributable to the risk factors) for each of cardiovascular diseases and neoplasms to manage the number of datapoints and lines. Collectively, these risk factors were high systolic blood pressure, dietary risks, tobacco smoke, high LDL cholesterol, high BMI, occupational risks, and high alcohol use, plus low physical activity as an upstream risk factor for both cardiovascular diseases and neoplasms. GBD definitions for risk factors were used, presented in appendix 1 (pp 4–5). The mean annual rate of change was calculated as the change between the start year and end year of a period divided by the number of years in that period. Analyses of changes over time and estimation of standard errors were done in R (version 4.2.1), with use of ggplot2 for visualisations.^17^, ^18^

### Role of the funding source

The funder of the study had no role in study design, data collection, data analysis, data interpretation, writing of the report, or the decision to submit the manuscript for publication.

## Results

**Figure 1:**
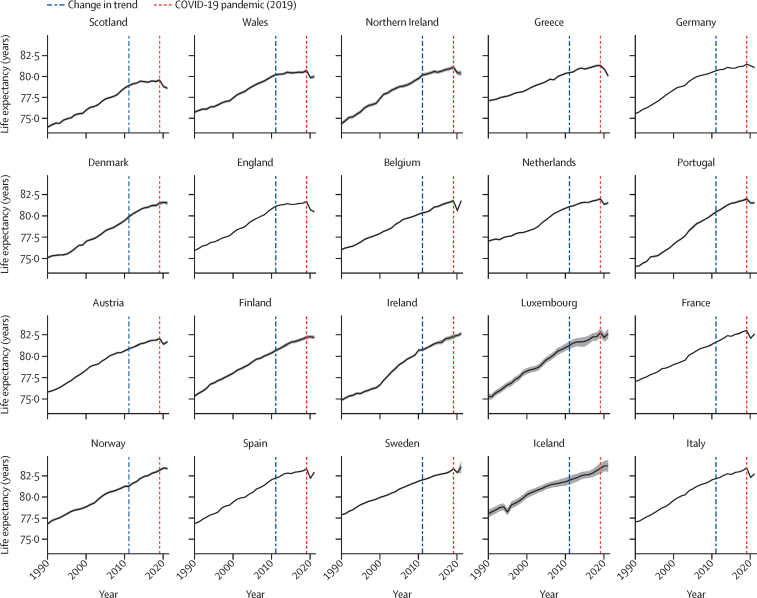
Life expectancy at birth for both sexes combined, from 1990 to 2021 by country, ordered by 2019 life expectancy 95% uncertainty intervals are shown as grey shading around the central lines.

Life expectancy steadily improved for at least two decades in all the included countries until around 2011, estimated by joinpoint regression modelling as the year when there was a statistically significant change in the slope of life expectancy improvement for the mean of all countries. Countries had different life expectancies and improvements over time (figure 1). All countries showed mean annual improvements in life expectancy in both 1990–2011 (overall mean 0·23 years [95% UI 0·23 to 0·24]) and 2011–19 (0·15 years [0·13 to 0·16]) but the rate of improvement varied substantially between countries. The rate of improvement was lower in 2011–19 than in 1990–2011 for all countries expect Norway, where the mean annual increase in life expectancy rose from 0·21 years (95% UI 0·20 to 0·22) in 1990–2011 to 0·23 years (0·21 to 0·26) in 2011–19 (difference of 0·03 years [95% UI 0·00 to 0·06]). Conversely, England showed the biggest decrease in the rate of improvement between these two periods, going from a mean annual increase in life expectancy of 0·25 (0·24 to 0·25) to 0·07 (0·06 to 0·08; difference of –0·18 years [–0·19 to –0·17]; table 1). Iceland had the smallest decrease (0·19 [0·16 to 0·21] *vs* 0·18 [0·09 to 0·26]; difference of –0·01 [–0·11 to 0·08]). Between 2019 and 2021, there was an overall decrease in mean annual life expectancy across all countries (overall mean –0·18 years [–0·22 to –0·13]), with all countries having an absolute fall in life expectancy except for Ireland, Iceland, Sweden, Norway, Denmark, and Belgium. The improvement was very marginal for Denmark, and marginal for Norway, Sweden, Iceland, and Ireland, due to the small improvements and relatively wide 95% UIs for this short period. Belgium showed no change in life expectancy during this period. The greatest decreases in 2019–21 were observed in Greece (mean annual change –0·61 [–0·70 to –0·51]) and England (–0·60 [–0·65 to –0·56]).

**Table 1:** Mean annual changes in life expectancy at birth in years by time periods, ordered by 2019 life expectancy

	**2019 life expectancy, years**	**1990 to 2011 change, years**	**2011 to 2019 change, years**	**2019 to 2021 change, years**	**Difference between 1990–2011 and 2011–19 changes, years**	**Difference between 2011–19 and 2019–21 changes, years**	**Population size in 2021, n**
Scotland	79·48 (79·32 to 79·62)	0·23 (0·22 to 0·24)	0·08 (0·05 to 0·10)	−0·48 (−0·57 to −0·36)	−0·15 (−0·18 to −0·12)	−0·56 (−0·67 to −0·43)	5 515 838
Wales	80·60 (80·43 to 80·76)	0·21 (0·20 to 0·22)	0·06 (0·03 to 0·09)	−0·35 (−0·48 to −0·23)	−0·15 (−0·18 to −0·11)	−0·42 (−0·56 to −0·27)	3 152 120
Northern Ireland	81·00 (80·76 to 81·22)	0·27 (0·26 to 0·29)	0·11 (0·08 to 0·15)	−0·35 (−0·55 to −0·16)	−0·16 (−0·20 to −0·11)	−0·46 (−0·68 to −0·25)	1 930 081
Greece	81·19 (81·04 to 81·34)	0·16 (0·15 to 0·16)	0·10 (0·08 to 0·12)	−0·61 (−0·70 to −0·51)	−0·05 (−0·08 to −0·03)	−0·71 (−0·81 to −0·61)	10 174 910
Germany	81·35 (81·28 to 81·41)	0·24 (0·24 to 0·24)	0·10 (0·09 to 0·11)	−0·20 (−0·23 to −0·15)	−0·14 (−0·15 to −0·13)	−0·29 (−0·34 to −0·25)	85 371 848
Denmark	81·49 (81·28 to 81·68)	0·23 (0·22 to 0·24)	0·20 (0·17 to 0·23)	0·01 (−0·10 to 0·11)	−0·03 (−0·06 to 0·01)	−0·19 (−0·31 to −0·07)	5 851 783
England	81·69 (81·63 to 81·74)	0·25 (0·24 to 0·25)	0·07 (0·06 to 0·08)	−0·60 (−0·65 to −0·56)	−0·18 (−0·19 to −0·17)	−0·67 (−0·72 to −0·62)	57 250 352
Belgium	81·76 (81·61 to 81·91)	0·21 (0·20 to 0·21)	0·18 (0·15 to 0·20)	0·00 (−0·08 to 0·07)	−0·03 (−0·06 to −0·01)	−0·17 (−0·26 to −0·09)	11 469 272
Netherlands	81·99 (81·86 to 82·11)	0·19 (0·19 to 0·20)	0·11 (0·09 to 0·13)	−0·23 (−0·29 to −0·17)	−0·08 (−0·10 to −0·06)	−0·34 (−0·42 to −0·27)	17 210 662
Portugal	82·01 (81·86 to 82·15)	0·30 (0·30 to 0·31)	0·19 (0·17 to 0·22)	−0·24 (−0·31 to −0·16)	−0·11 (−0·13 to −0·08)	−0·43 (−0·52 to −0·35)	10 607 849
Austria	82·07 (81·91 to 82·21)	0·24 (0·23 to 0·25)	0·15 (0·13 to 0·17)	−0·19 (−0·27 to −0·11)	−0·09 (−0·12 to −0·07)	−0·34 (−0·43 to −0·25)	8 982 312
Finland	82·22 (82·00 to 82·43)	0·25 (0·24 to 0·26)	0·19 (0·15 to 0·22)	−0·02 (−0·12 to 0·10)	−0·06 (−0·11 to −0·03)	−0·21 (−0·33 to −0·07)	5 535 925
Ireland	82·31 (82·07 to 82·56)	0·28 (0·27 to 0·29)	0·20 (0·16 to 0·23)	0·16 (0·03 to 0·28)	−0·09 (−0·13 to −0·05)	−0·04 (−0·18 to 0·11)	4 941 374
Luxembourg	82·72 (82·22 to 83·21)	0·29 (0·26 to 0·31)	0·18 (0·10 to 0·26)	−0·05 (−0·23 to 0·14)	−0·11 (−0·20 to −0·02)	−0·23 (−0·42 to −0·02)	644 266
France	82·99 (82·92 to 83·06)	0·22 (0·22 to 0·22)	0·17 (0·16 to 0·18)	−0·21 (−0·26 to −0·16)	−0·05 (−0·06 to −0·04)	−0·38 (−0·43 to −0·33)	66 389 877
Norway	83·08 (82·95 to 83·22)	0·21 (0·20 to 0·22)	0·23 (0·21 to 0·26)	0·10 (0·00 to 0·20)	0·03 (0·00 to 0·06)	−0·13 (−0·24 to −0·02)	5 418 070
Spain	83·24 (83·16 to 83·32)	0·25 (0·25 to 0·26)	0·13 (0·12 to 0·15)	−0·19 (−0·24 to −0·15)	−0·12 (−0·13 to −0·10)	−0·33 (−0·38 to −0·27)	45 549 328
Sweden	83·26 (83·15 to 83·36)	0·19 (0·19 to 0·20)	0·16 (0·15 to 0·18)	0·11 (−0·24 to 0·45)	−0·03 (−0·05 to −0·01)	−0·06 (−0·41 to 0·29)	10 373 513
Iceland	83·31 (82·66 to 83·92)	0·19 (0·16 to 0·21)	0·18 (0·09 to 0·26)	0·15 (−0·01 to 0·31)	−0·01 (−0·11 to 0·08)	−0·02 (−0·21 to 0·16)	350 386
Italy	83·37 (83·32 to 83·42)	0·24 (0·24 to 0·24)	0·16 (0·15 to 0·17)	−0·36 (−0·40 to −0·32)	−0·08 (−0·09 to −0·08)	−0·52 (−0·56 to −0·47)	59 811 452
**Overall**	NA	0·23 (0·23 to 0·24)	0·15 (0·13 to 0·16)	−0·18 (−0·22 to −0·13)	−0·08 (−0·10 to −0·07)	−0·32 (−0·37 to −0·28)	NA

Numbers in parentheses are 95% uncertainty intervals. Values are rounded to two and three decimal places. Population sizes in 2021 were sourced from the GBD Results tool. NA=not applicable.

**Figure 2:**
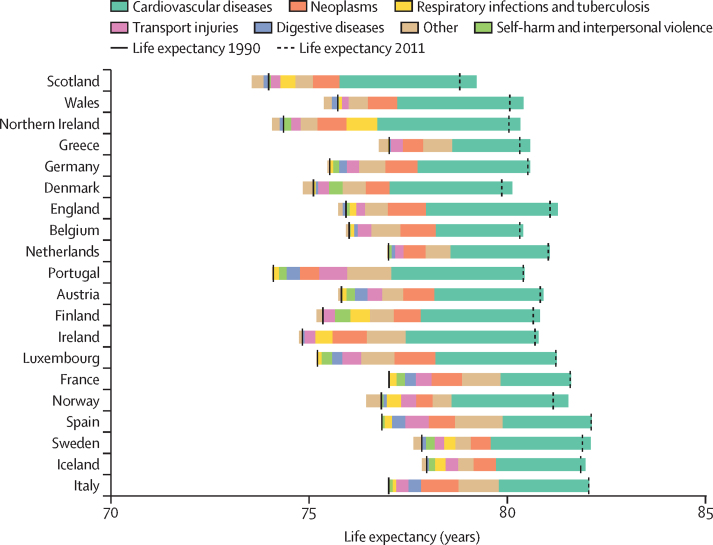
Changes in life expectancy at birth for both sexes combined, by country and cause of death from 1990 to 2011, ordered by 2019 life expectancy The solid vertical black bars show life expectancy in 1990 for each country, and the dashed vertical black bars show life expectancy in 2011. The coloured bars to the right of the 1990 life expectancy line represent the number of years of improvement that were attributed to specific causes of death. Any coloured bars to the left of the 1990 line represent years of worsening of life expectancy attributed to specific causes of death between 1990 and 2011. Coloured bars to the right of the 2011 life expectancy line represent years of improvement attributed to specific causes of death, which are equal to the number of years to the left of the 1990 line. Further details on the methodology for the figure are provided in appendix 1 (p 6). Small proportions visible in appendix 1 (p 1) might not be visible in this figure due to the difference in scale.

**Figure 3:**
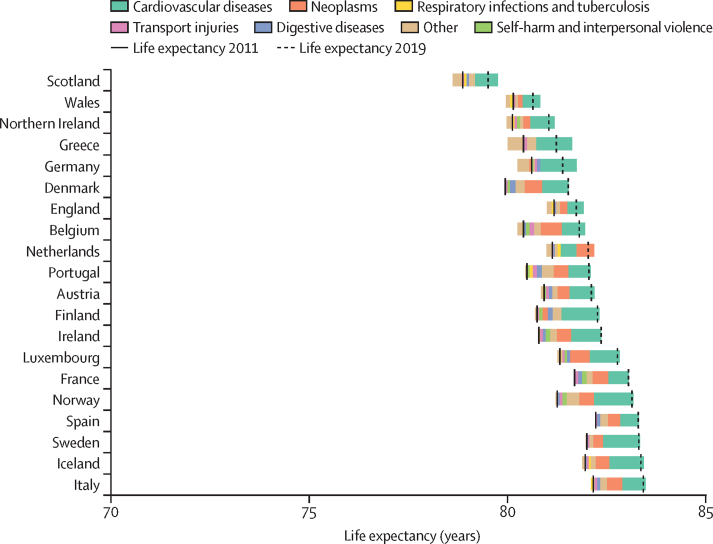
Changes in life expectancy at birth for both sexes combined, by country and cause of death from 2011 to 2019, ordered by 2019 life expectancy The solid vertical black bars show life expectancy in 2011 for each country, and the dashed vertical black bars show life expectancy in 2019. The coloured bars to the right of the 2011 life expectancy line represent the number of years of improvement that were attributed to specific causes of death. Any coloured bars to the left of the 2011 line represent years of worsening of life expectancy attributed to specific causes of death between 2011 and 2019. Coloured bars to the right of the 2019 life expectancy line represent years of improvement attributed to specific causes of death, which are equal to the number of years to the left of the 2011 line. Further details on the methodology for the figure are provided in appendix 1 (p 6). Small proportions visible in appendix 1 (p 1) might not be visible in this figure due to the difference in scale.

**Figure 4:**
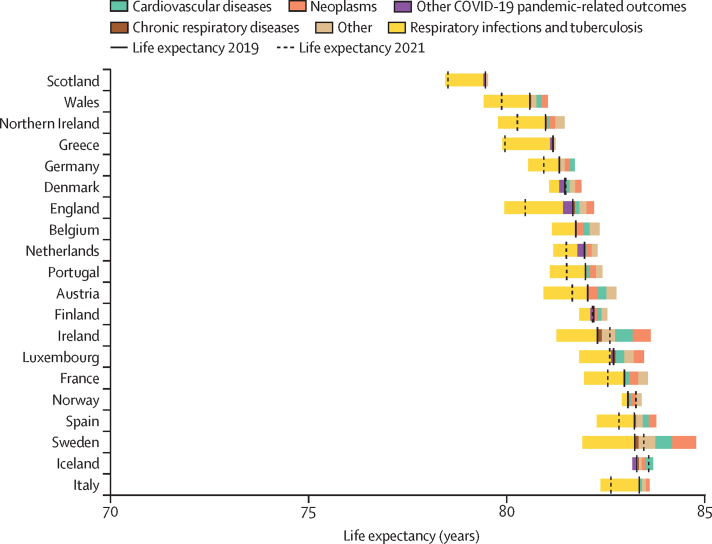
Changes in life expectancy at birth for both sexes combined, by country and cause of death from 2019 to 2021, ordered by 2019 life expectancy The solid vertical black bars show life expectancy in 2019 for each country, and the dashed vertical black bars show life expectancy in 2021. The coloured bars to the right of the 2019 life expectancy line represent the number of years of improvement that were attributed to specific causes of death. Any coloured bars to the left of the 2019 line represent years of worsening of life expectancy attributed to specific causes of death between 2019 and 2021. Bars on the outsides of the solid and dashed lines represent equal numbers of years. Further details on the methodology for the figure are provided in appendix 1 (p 6).

The causes of death responsible for the largest improvements in life expectancy from 1990 to 2011 were cardiovascular diseases and neoplasms, shown by the decomposition analysis (figure 2). The countries where gains in life expectancy attributed to these causes of death were similar going from 1990–2011 to 2011–19 were also countries that best maintained improvements in life expectancy between 1990–2011 and 2011–19: Norway, Iceland, Belgium, Denmark, and Sweden (figure 3, table 1, appendix 1 p 1). Although Denmark was one of the best at maintaining improvements in life expectancy post-2011, the contribution of cardiovascular disease-related deaths to life expectancy gains was reduced in Denmark after 2011 while the contribution of neoplasm-related deaths was increased. These countries also maintained or marginally improved life expectancy from 2019 to 2021 during the COVID-19 pandemic, when life expectancy decreased in all other countries except Ireland. During 2019–21, in countries where life expectancy decreased, the decreases were entirely attributable to deaths from respiratory infections and other COVID-19-related outcomes apart from in Greece (where a very small portion of the decrease was also attributable to deaths from neoplasms; figure 4, table 1).

Up until the COVID-19 pandemic, deaths from cardiovascular diseases were the primary driver of reductions in life expectancy improvement from pre-2011 to post-2011, with reductions in the amount that deaths from cardiovascular diseases contributed to improvements going from 1990–2011 to 2011–19 in all countries except Greece (appendix 1 p 1). Countries varied in how well they maintained progress with neoplasm-related mortality during 2011–19. Norway, Denmark, Belgium, the Netherlands, Iceland, Portugal, Sweden, Luxembourg, France, and Spain had larger gains in life expectancy attributed to deaths from neoplasms in 2011–19 versus 1990–2011, whereas England, Wales, Northern Ireland, and Finland had smaller gains in life expectancy attributed to deaths from neoplasms in the post-2011 period (appendix 1 p 1). Germany, Scotland, and Greece had decreases in life expectancy attributable to neoplasms in 2011–19. Austria, Ireland, and Italy had little change in the gains in life expectancy attributable to deaths from neoplasms between 1990–2011 and 2011–19.

Among the studied countries, those with the greatest slowdown in life expectancy improvements before the COVID-19 pandemic were generally most severely affected by COVID-19 and had some of the largest decreases in life expectancy in 2019–21 (Figure 3, Figure 4, table 1). Greece, the four UK nations, and Italy had the largest decreases in life expectancy (mean annual change ranging from –0·35 years [95% UI –0·55 to –0·16] in Northern Ireland and –0·35 years [–0·48 to –0·23] in Wales, to –0·61 years [–0·70 to –0·51] in Greece) between 2019 and 2021. As aforementioned, Ireland, Iceland, Sweden, Norway, and Denmark maintained marginal improvements (mean annual change ranging from 0·01 years [–0·10 to 0·11) in Denmark to 0·16 years [0·03 to 0·28] in Ireland). Sweden and Ireland both had a high number of deaths from respiratory infections during 2019–21, but maintained an overall improvement in life expectancy due to decreased deaths from cardiovascular diseases and neoplasms. By comparison, the UK nations (Scotland in particular), Greece, and Italy showed decreases in life expectancy because these countries had high death rates due to respiratory infections and other COVID-19-related outcomes, while making little or no progress with cardiovascular diseases and neoplasms.

The specific risk factors with the three highest mean age-standardised death rates (per 100 000 of the population) attributable to cardiovascular diseases in 2019, for both sexes in all countries combined, were high systolic blood pressure (54·25 per 100 000), dietary risks (27·74 per 100 000), and high LDL cholesterol (23·02 per 100 000). The top three risk factors for neoplasms were tobacco smoke (27·30 per 100 000), dietary risks (9·83 per 100 000), and occupational risks (8·89 per 100 000). The major risk factors observed for both cardiovascular diseases and neoplasms were dietary risks, tobacco smoke, high BMI, high fasting plasma glucose, air pollution, other environmental risks, and low physical activity. Death rates for the top 10 risk factors plus physical activity for both causes are provided in table 2.

**Table 2:** Mean age-standardised death rates (per 100 000 of the population) attributable to specific risk factors for cardiovascular diseases and neoplasms for both sexes for all countries combined in 2019

	**Deaths per 100 000 of the population**
**Cardiovascular diseases**	
High systolic blood pressure	54·25
Dietary risks	27·74
High LDL cholesterol	23·02
Tobacco smoke	13·95
High BMI	12·80
Kidney dysfunction	11·74
High fasting plasma glucose	11·54
Non-optimal temperature	6·99
Air pollution	5·56
Other environmental risks	4·60
Low physical activity	2·34
**Neoplasms**	
Tobacco smoke	27·30
Dietary risks	9·83
Occupational risks	8·89
High BMI	6·48
High alcohol use	5·96
High fasting plasma glucose	4·88
Low physical activity	1·78
Other environmental risks	1·76
Air pollution	1·60
Unsafe sex	1·30

Global Burden of Disease Study risk factor definitions are provided in appendix 1 (pp 4–5).

The SEVs for the top five attributable risk factors for each of cardiovascular diseases and neoplasms changed over time from 1990 to 2021, with some consistent patterns (appendix 1 p 2). Exposure to tobacco smoke remained a high population risk but exposure decreased steadily in all countries from 1990 to 2021, in contrast to the SEV for high BMI, which steadily increased in all countries. Reductions in the SEVs for high LDL cholesterol slowed or began to reverse in most countries around 2011, and reductions in the SEVs for high systolic blood pressure slowed or began to reverse in many countries. Exposure to other risk factors including dietary risks, high alcohol use, and low physical activity remained elevated or slightly increased in most countries over the three decades. There were some examples where countries that maintained improvements in life expectancy up to 2021 tended to have favourable risk factor trends, although the patterns were complex. For example, comparing England and Sweden, Sweden had a continued reduction in SEV for high systolic blood pressure after 2011, whereas improvements stalled in England, and Sweden had substantially lower SEVs than England for low physical activity over the three decades.

A scatter graph of change in life expectancy against change in SEVs for the top five attributable risk factors for each of cardiovascular diseases and neoplasms by country was used to identify associations and compare the two periods of 1990–2011 and 2011–19 (appendix 1 p 3). This graph complemented the timelines for SEVs (appendix 1 p 2) and showed that SEVs for high systolic blood pressure and high LDL cholesterol were reducing in many countries before 2011 but increasing after 2011. These increasing SEVs after 2011 generally coincided with decreasing annual improvements in life expectancy. SEVs for high BMI were increasing in all countries in both periods, and SEVs for dietary risks and low physical activity remained reasonably constant with minor increases for several countries in each period. Tobacco smoke exposure continued to improve after 2011. These steady or increasing exposures to high BMI, dietary risks, and low physical activity were associated with slowing improvements in life expectancy over time, despite the benefits to be expected from reduced exposure to tobacco smoke over the same time period.

## Discussion

### Main findings

All countries in this study except Norway had a slowing in life expectancy improvements after 2011, but the slowdown was greater in some countries than others. Improvements in mortality from cardiovascular diseases and neoplasms slowed substantially in many countries after 2011, as did improvements in high LDL cholesterol and high systolic blood pressure. High BMI steadily increased over the three decades and other risks including dietary risks, high alcohol use, and low physical activity remained high in most countries. Exposure to tobacco smoke decreased steadily in all countries, but remains important. There were notable international differences in life expectancy improvement, with Norway, Iceland, Sweden, Denmark, and Belgium continuing to make progress after 2011. These countries (except Belgium) also maintained improvements in life expectancy in 2019–21 during the COVID-19 pandemic, when life expectancy decreased in all other countries except Ireland.

Various underlying factors are likely to explain the observed patterns in cause-specific death rates and trends in risk factors observed in this study. These include structural, economic, commercial, and environmental determinants of health and illness, access to high quality preventive health care and treatment, and individual behavioural factors. The relative contribution of each factor is difficult to ascertain from statistical analyses such as ours and remains the subject of considerable debate. The findings reported in this paper do not support the hypothesis that the slowing of life expectancy improvements is because a natural longevity ceiling has been reached at around 110 years of age.^19^ The GBD 2019 life tables estimate age-specific mortality rates above 110 years, reflecting the continued rise in life expectancy for older people in many countries.^20^ Even if some countries were approaching a longevity ceiling, life expectancy is driven by changes in mortality below age 100 years, where there remains considerable scope for reduced mortality and key risks.

### Health services

Reductions in cardiovascular risk factors, particularly high blood pressure and tobacco smoking, explained 30–40% of the reduction in cardiovascular mortality in England in 2000–07, and improvements in medical treatments (including antihypertensives and cholesterol-lowering medication) explained approximately half of the decrease in cardiovascular mortality between 1995 and 2008 in Turkey.^21^, ^22^ It is possible that a limit is being reached on the reductions that can be achieved through current medical treatments, although surveys suggest that there are still many individuals who would benefit from preventive treatments who are not yet receiving them, particularly among socioeconomically deprived subgroups.^23^

In this study, the worsening trend in population exposure to high LDL cholesterol and high systolic blood pressure after 2011 in many countries suggests that improved treatment for individuals with raised lipid concentrations or blood pressure is not sufficient to offset the effect of adverse population changes, for example in BMI, or continued high exposure to dietary risks. Italy has one of the highest life expectancies in the world, which has been attributed to the quality of the universal health system and healthy behaviours.^24^ However, life expectancy in Italy decreased substantially in 2019–21 with a downturn in life expectancy gains due to neoplasms and cardiovascular diseases from pre-2019 to post-2019 for uncertain reasons, but possibly associated with reduced spending on public health and preventive measures rather than a specific policy. National policies that address diagnosis, treatment, and prevention of neoplasms through reduction in risks have been implemented to different degrees in different countries. Belgium, France, and Norway all increased their cancer diagnosis and treatment activity in recent years through national policies^25^, ^26^, ^27^ and have maintained progress in improving life expectancy linked to neoplasms between 1990–2011 and 2011–19 (appendix 1 p 1). Belgium's National Cancer Plan for 2008–10 emphasised prevention (screening and action on smoking, alcohol, and food) as well as treatment.^26^ In Norway, national guidance to standardise prevention, diagnosis, and monitoring for breast, lung, colon, and prostate cancer was issued in 2013 as one of an ongoing series of National Cancer Strategies.^27^ Similar approaches to the prevention and treatment of cardiovascular diseases have been shown to be effective, for example the National Service Framework approach adopted in England in 2000.^28^

### Risk factors for cardiovascular diseases and neoplasms

Four industry sectors (tobacco, ultra-processed food, fossil fuel, and alcohol) are responsible for at least a third of global deaths, and the power of the commercial sector tends to prevent the implementation of effective policies to mitigate risks.^29^ A partial exception is tobacco, for which sustained action over decades has reduced exposure, although tobacco smoke remains the biggest risk factor for cancer by some margin (table 2), and a continued policy focus on tobacco smoking is essential to maintain the current gains. Successful actions to reduce the harm from tobacco offer a template for reducing alcohol and dietary risks. Effective national policies on minimum unit pricing to reduce the consumption of alcohol exist, but are not being widely implemented.^30^, ^31^ Risks related to alcohol consumption are increased in people with low economic status due to the coexistence of other risk factors such as smoking and heavy episodic drinking.^32^

The effects of upstream risk factors, such as diet and low physical activity, are particularly important as they have a wide range of beneficial effects, some of which are mediated through high systolic blood pressure, high LDL cholesterol, and high plasma glucose, and some through other mechanisms which are less well understood.^33^ Dietary risk in GBD is an aggregate risk factor for a diet low in wholegrains, fruit, fibre, legumes, nuts and seeds, omega-3 fatty acids, polyunsaturated fats, vegetables, milk, and calcium; and a diet high in sodium, trans fats, red or processed meat, and sugar-sweetened beverages (appendix 1 p 4).^34^ There is strong evidence that improving diet offers scope for large population health gains: in a meta-analysis of studies comparing a Mediterranean diet with unrestricted fat intake with other diets, the Mediterranean diet reduced major cardiovascular events by around 30% and cancer mortality by 25%.^35^, ^36^ The EAT–*Lancet* Commission defines a healthy diet as one that provides plenty of fruit, vegetables, wholegrains, and healthy proteins, with minimal saturated fats, highly processed foods, and added sugar.^37^

Variation in diet between countries reflects a combination of cultural, social, economic, and policy factors, but can still be influenced by sustained policy intervention. For example, Norway has a long history of fiscal intervention to reduce sugar consumption and protect domestic food production, as well as strong social security and policies aiming for regional balance in economic growth, sustainability, travel, and access to education and health services.^38^ A tax on sugar has been in place since 1922 and the Norwegian Government consulted with industry as early as the 1980s to reduce the amount of salt in food products. A cross-sectoral national plan also led to improvements in the national diet.^39^ This approach has been more successful than voluntary and educational approaches, such as the Scottish Government policy since 2001 to make healthy foods more widely available, primarily through voluntary reformulation of foods and improving consumer knowledge. This approach led to little change in the proportion of people in Scotland who consumed the recommended amount of five or more portions per day of fruit and vegetables.^40^

Levels of physical activity are assessed in GBD based on the frequency, duration, and intensity of activity. The wide-ranging effects of physical activity are reflected in the benefits seen in large observational studies of regular physical activity, which consistently report a 20–30% risk reduction in premature mortality and the incidence of several chronic health conditions.^41^ Individuals who are sedentary experience substantial health benefits from even a small increase in their physical activity and there is no minimum threshold for benefit.^41^ However, at a population level in the current analysis, there had been little change in exposures to low physical activity across the studied countries despite the strengthening evidence for its benefits. Again, coordinated systematic strategies are required to achieve improvement in levels of physical activity at the population level. A further incentive to do so is the clear additional benefit of an active travel policy in reducing climate risk.

### Fiscal policies

The links between poverty and health and life expectancy are well established and are partly mediated through the biomedical risk factors considered in this paper. The biomedical risk factors are prioritised when quantifying the effect of poverty on health by the GBD selection of risk–outcome pairs, which depends on robust quantitative estimates of effect size. The broader determinants of health, such as fiscal policies, also influence population health more widely than can be captured by changes in measurable biomedical risk factors.^11^, ^42^, ^43^, ^44^ A worsening economic position could increase short-term mortality in the most socioeconomically deprived individuals as well as increasing longer term exposure to risks such as poverty, poor diet, food insecurity, low pay and employment levels, and poor housing.^42^ Among 28 EU countries between 1991 and 2013, austerity regimes were associated with an overall 0·7% increase in all-cause mortality, and similar findings have been observed in England and Wales.^45^, ^46^

Most countries had some degree of reduced spending on public services and benefits following the economic recession of 2007–08, and although deficit reduction policies varied considerably by country, health expenditure plateaued in nearly all countries.^47^ The 2007–08 recession was followed by adverse impacts on health and social wellbeing across Europe (for example, through unemployment and poverty) in addition to health service cuts.^48^ In the UK, a National Health Inequalities Strategy between 1997 and 2010 coincided with a reduction in health inequalities in England, overall and for cardiovascular diseases and some cancers.^49^ Subsequent large funding cuts to health, social care, and welfare since 2010, particularly in areas of socioeconomic deprivation, affected the social determinants of health and therefore contributed to the slowdown in mortality improvement.^50^, ^51^

### Cohort effects

National and international cohort effects might be contributing to the slowdown in life expectancy improvements. Populations born around the same time and sharing specific life experiences might have increased or decreased mortality compared with other groups born before or after that period. For example, in Scotland in the early 1980s, there was widespread deindustrialisation and unemployment, which might have led to negative future health effects for those children whose parents were unemployed or for unemployed young adults.^52^ These people will have had increased exposure to risk factors such as high BMI, tobacco smoke, dietary risks, and alcohol use. In Scotland, there is evidence of a cohort effect on drug-related deaths and suicide, but weaker evidence for a cohort effect on ischaemic heart disease and stroke.^52^, ^53^, ^54^, ^55^, ^56^ Another example of a cohort effect is the increased mortality in the USA in individuals who were born during the Great Depression of 1929 compared to those born before or after this period.^57^ The long latency of these cohort effects needs to be considered in any analysis of future scenarios.

### Strengths and limitations

A major strength of the GBD method is that it goes to great lengths to produce comparable and consistent estimates of mortality, causes of death, and risk factors. GBD methods for estimating life expectancy differ slightly from those used by Eurostat which inform OECD estimates of life expectancy. Eurostat uses data from national statistical offices and Farr's method, which calculates life expectancy with use of life tables presenting age-specific mortality rates.^58^ GBD adds additional steps, evaluating the completeness of vital registration data via death distribution methods.^59^ Life expectancy estimates produced by individual nations might also differ from both GBD and OECD estimates because of methodological inconsistencies, but the overall trends are similar. We did not present results for healthy life expectancy as well as life expectancy due to space limitations and because the two measures give similar results, except that there were slightly smaller gaps between male and female individuals with respect to healthy life expectancy (with female individuals having higher life expectancy; data not shown).

In this paper, we chose fixed periods to evaluate differences and combined findings for both sexes. Although this allows for a consistent approach, changes in life expectancy trends occurred at different times for different countries. Any differences would be exacerbated or underestimated if different periods were chosen, or if estimates for male and female individuals were analysed separately. For example, the increase in life expectancy in Denmark, Finland, and Portugal appeared to slow down in 2015 or 2016, compared with 2010 in England. Trends in life expectancy and both risk exposure and mortality vary between male and female individuals, such as in Finland and the UK.^60^ Differences in male and female individuals might, for example, reflect broader inequalities in society. In Sweden and Norway, the gender gaps in labour market participation and employment are among the smallest of the OECD countries, and this might be a contributor to the continued rise in life expectancy that we observed up to 2021.^61^

The production of cause-specific estimates of mortality relies on accurately coded death certificates that correctly consider comorbidities at the time of death. Where necessary, GBD redistributes inaccurate causes of deaths to more accurate plausible causes of death.^12^ Attributable risk estimates within GBD need to be interpreted with caution because of modelling assumptions. The lag time between exposure and harm varies for different risk factors, and some major risks such as tobacco smoking and obesity can have an effect over decades. Exposure before our start date of 1990 has not been considered in the analyses. GBD does not have data to explore the effect on life expectancy of immigration, including asylum seekers, into Europe between 1990 and 2021.

The evidence-based focus on single proximal determinants of health does not capture the effect of risk factors acting in combination, where the evidence is scant. In general, the quality and extent of the evidence base correlates poorly with the public health importance of many risks. This explains why GBD includes few risk factors associated with wider determinants of health, such as poverty, employment, and housing, for which the non-observational evidence base is scarce due to the unfeasibility of conducting trials of interventions.

### Implications for policy and research

The present results can guide action by policy makers seeking to reverse the slowdown in life expectancy improvements in the studied European nations. The immediate signal was changing mortality patterns, and the underlying population risk factors are clear. Improvements in upstream risk factors such as dietary risks and physical inactivity offer the potential for substantial improvements in mortality across multiple conditions.^13^

Country-specific public health and health-care policies can have a positive impact. Policy responses should build on the experience of those countries who have implemented successful health-care and public health strategies. Multiple factors have contributed to the slowdown in life expectancy, and coordinated action across multiple sectors will be needed to reverse it. The wider economic, social, and commercial determinants of health are particularly important as they affect multiple conditions, and a Health in All Policies approach is needed to address these determinants and to reduce health inequalities.^62^, ^63^ The growing evidence of an association between fiscal austerity and changes in life expectancy implies that addressing the continuing shortfall in services, especially for the most socioeconomically deprived and for marginalised groups, will be an essential component of any recovery plan.^64^, ^65^

The link between pre-existing general health and mortality during the COVID-19 pandemic period shows the need to build resilience in a population by addressing non-communicable diseases as well as reducing the risk of infection. An unequal society with limited social welfare will see early spreading of infectious disease among populations who live in crowded multiple occupancy households, those who have a high prevalence of multimorbidity, exacerbating poor outcomes from infection, and those who cannot afford to isolate and take time off work. The public health control measures implemented during the COVID-19 pandemic (including closures to schools and the hospitality sector) disproportionately affected deprived communities, exacerbating existing inequalities.^66^ A life course approach is likely to be needed to minimise the long-term impact of future major adverse events such as the financial crash of 2007–08 or the COVID-19 pandemic, and to reduce risks to future health.^67^

### Conclusions

Gains in life expectancy have slowed and in most cases life expectancy has decreased across the included European countries between 1990 and 2021. These findings show that the stalled progress in reducing deaths from the major causes of cardiovascular diseases and cancer is attributable to changes in population exposure to common risk factors, including high BMI, and continued high exposure to dietary risks. Trends in life expectancy at the national level are associated with major long-term policy interventions, implying that governments can substantially influence the longevity of their population through policy choices that should include addressing the commercial determinants of health, reducing dietary risks, improving physical activity levels, and ensuring access to effective health care for prevention and treatment. Further development of long-term national and international cross-sectoral strategies, involving governments, communities, schools, and employers, is urgently needed to reverse the slowdown in life expectancy improvements and worsening life expectancy over the past 15 years in European nations.

### GBD 2021 Europe Life Expectancy Collaborators

### Affiliations

### Contributors

### Data sharing

Citations and metadata for all input data used in this analysis are available for download from the GBD 2021 Sources Tool (https://ghdx.healthdata.org/gbd-2021/sources). To visualise or download the estimates produced in this analysis, please visit the GBD Compare tool (https://vizhub.healthdata.org/gbd-compare/) and GBD Results tool (https://vizhub.healthdata.org/gbd-results/). A Global Health Data Exchange record with the life expectancy decomposition estimates is available from: https://ghdx.healthdata.org/record/ihme-data/gbd-2021-europe-le-change-cause-1990-2021.

## Declaration of interests

J Ärnlöv reports payment or honoraria for lectures from AstraZeneca, Boehringer Ingelheim, and Novartis; and participation on a data safety monitoring board or advisory board with AstraZeneca, Astella, and Boehringer Ingelheim; all outside the submitted work. T Bärnighausen reports grants or contracts paid to their institution from National Institutes of Health, Alexander von Humboldt Foundation, German National Research Foundation, EU, German Ministry of Education and Research, German Ministry of the Environment, Wellcome, and KfW; payment or honoraria for lectures, presentations, speakers bureaus, manuscript writing, or educational events from *PLOS Medicine* as Editor-in-Chief; participation on advisory boards for NIH-funded research projects in Africa on climate change and health, unpaid; and stock or stock options in the Climate Change and Health Evaluation and Response System (CHEERS), a small-to-medium-sized enterprise focusing on approaches to measure climate change and health-related variables in population cohorts; all outside the submitted work. J L Baker reports grants or contracts paid to their institution from Novo Nordisk Foundation, World Cancer Research Fund, Independent Research Council Denmark, and EU Horizon; consulting fees from Novo Nordisk; payment or honoraria for lectures, presentations, speakers bureaus, manuscript writing, or educational events paid to their institution from Novo Nordisk; support for attending meetings and/or travel with the European Association for the Study of Obesity; participation on a data safety monitoring board or advisory board with Novo Nordisk, paid to their institution; and leadership or fiduciary roles in board, society, committee, or advocacy groups, paid or unpaid, with the European Association for the Study of Obesity; all outside the submitted work. S Barteit reports support for attending meetings and/or travel with the Wellcome Trust; and stock or stock options with CHEERS; all outside the submitted work. M Beghi reports payment or honoraria for lectures, presentations, speakers bureaus, manuscript writing, or educational events from Angelini and Lundbeck, all outside the submitted work. Y Bejot reports consulting fees from Medtronic, Novartis, and Boehringer Ingelheim; payment or honoraria for lectures, presentations, speakers bureaus, manuscript writing, or educational events from BMS, Pfizer, Medtronic, Amgen, NovoNordisk, and Servier; support for attending meetings and/or travel with Medtronic; and leadership or fiduciary roles in other board, society, committee, or advocacy groups, unpaid, with the French Neurovascular Society; all outside the submitted work. M Bell reports grants or contracts paid to their institution from US EPA, NIH, Hutchinson Postdoctoral Fellowship, Health Effects Institute, Yale Women Faculty Forum, Robert Wood Johnson Foundation, Yale Institute for Biospheric Studies, and Wellcome Trust Foundation; consulting fees from Clinique and ToxiMap; payment or honoraria for lectures, presentations, speakers bureaus, manuscript writing, or educational events from Colorado School of Public Health, Duke University, University of Texas, Data4Justice, Korea University, University of Pennsylvania (for speaking), IOP Publishing (for editorial duties), NIH, Health Canada, EHS, PAC-10, UKRI, AXA Research Fund Fellowship, University of Texas (for grant review), Korea University (for research), Harvard University and University of Montana (on an external advisory committee), and SciQuest (for an online survey/workshop); support for attending meetings and/or travel from Colorado School of Public Health, University of Texas, Duke University, Harvard University, American Journal of Public Health, Columbia University, Harvard University, CMAS Conference, Nature Conference; and leadership or fiduciary roles in other board, society, committee, or advocacy groups from Fifth National Climate Assessment (unpaid), *Lancet* Countdown (unpaid), US EPA Clean Air Scientific Advisory Committee (paid), Johns Hopkins EHE Advisory Board (unpaid), Harvard External Advisory Committee for a training grant (unpaid), WHO Global Air Pollution and Health Technical Advisory Group (unpaid), National Academies Panels and Committees (unpaid); all outside the submitted work. L Belo reports support from FCT in the scope of the project UIDP/04378/2020 and UIDB/04378/2020 of UCIBIO and the project LA/P/0140/2020 of i4HB. A Beloukas reports grants or contracts paid to the University of West Attica from Gilead and GSK/ViiV; payment or honoraria for lectures, presentations, speakers bureaus, manuscript writing, or educational events paid to the University of West Attica from Gilead and GSK; support for attending meetings and/or travel paid to the University of West Attica from Gilead and GSK; and receipt of equipment, materials, drugs, medical writing, gifts or other services from Cepheid in the form of FOC reagents for a research project; all outside the submitted work. P J G Bettencourt reports patents planned, issued or pending (numbers WO2020229805A1, BR112021022592A2, EP3965809A1, OA1202100511, US2023173050A1, EP4265271A2, EP4275700A2); and other support from Botnar Foundation as a project reviewer; all outside the submitted work. S Bhaskar reports grants or contracts from the Japan Society for the Promotion of Science (JSPS), Japanese Ministry of Education, Culture, Sports, Science and Technology (for a Grant-in-Aid for Scientific Research, grant ID: 23KF0126) and from JSPS and the Australian Academy of Science for a JSPS International Fellowship (grant ID: P23712); and leadership or fiduciary roles in board, society, committee, or advocacy groups, paid or unpaid, with Rotary District 9675, Sydney, Australia, as the District Chair (Diversity, Equity & Inclusion), with the Global Health & Migration Hub Community, Global Health Hub Germany, Berlin, Germany (Chair, Founding Member and Manager), with *PLOS One, BMC Neurology, Frontiers in Neurology, Frontiers in Stroke, Frontiers in Public Health, Journal of Aging Research, Neurology International, Diagnostics*, and *BMC Medical Research Methodology* (Editorial Board Member), with the College of Reviewers, Canadian Institutes of Health Research, Government of Canada (Member), with the World Headache Society, Bengaluru, India (Director of Research), with the Cariplo Foundation, Milan, Italy (Expert Adviser/Reviewer), with the National Cerebral and Cardiovascular Center, Department of Neurology, Suita, Osaka, Japan (Visiting Director), with Cardiff University Biobank, Cardiff, UK (Member, Scientific Review Committee), and with the Rotary Reconciliation Action Plan (Chair); all outside the submitted work. A L Catapano reports grants or contracts from Amryt Pharma, Menarini, and Ultragenyx; consulting fees from Menarini–Menarini Ricerche and Sanofi; and payment or honoraria for lectures, presentations, speakers bureaus, manuscript writing, or educational events from Amarin Amgen Amryt Pharma, Astrazeneca, Daiichi Sankyo Esperion Ionis Pharmaceutical Medscaper, Menarini, Merck, Novartis, NovoNordisk, Peervoice Pfizer Recordati Regeneron, Sandoz, Sanofi The Corpus, Ultragenyx, and Viatris; all outside the submitted work. J Conde reports grants or contracts from the European Research Council Starting Grant (ERC-StG-2019-848325); and patents planned, issued, or pending with Universidade Nova de Lisboa (Surfactant-Based Hydrogel, Methods and Uses Thereof); all outside the submitted work. S Cortese reports grants or contracts from NIHR and the European Research Agency; and payment or honoraria for lectures, presentations, speakers bureaus, manuscript writing, or educational events from the British Association of Psychopharmacology, Canadian ADHD Resource Alliance, Medice, and the Association for Child and Adolescent Mental Health; all outside the submitted work. S Das reports leadership or fiduciary roles in board, society, committee, or advocacy groups, paid or unpaid, with the Association for Diagnostics and Laboratory Medicine (ADLM; Program Chair), with the Academy Membership Committee of ADLM (Member), ADLM Genomics Division (General Secretary), and CLSI USA (Member of Expert Committee and Nominating Committee), all outside the submitted work. A K Demetriades reports non-fiduciary leadership roles in board, society, committee or advocacy groups, paid or unpaid, with the European Association of Neurosurgical Societies (Immediate Past President) and the Global Neuro Foundation (Vice-President), all outside the submitted work. T C Ekundayo reports grants or contracts from the University of South Africa outside the submitted work. S-M Fereshtehnejad reports payment or honoraria for lectures, presentations, speakers bureaus, manuscript writing, or educational events from Health Advances and the European Science Foundation, outside the submitted work. N Ghith reports grants or contracts from the Novo Nordisk Foundation (NNF16OC0021856) for her salary during her employment at the Technical University of Denmark between 2019 and 2022; and support for attending meetings and/or travel from the Danish Data Science Institute at the Technical University of Denmark; all outside the submitted work. P Gill reports support for the present manuscript from the UK NIHR Senior Investigator Award paid to the University of Warwick. M Hultström reports support for the present manuscript from the Swedish Heart Lung Foundation and the Knut och Alice Wallenberg Foundation, paid to their institution. M Hultström also reports payment or honoraria for lectures, presentations, speakers bureaus, manuscript writing, or educational events from the Swedish Society of Anaesthesiology and Intensive Care; support for attending meetings and/or travel from the American Physiological Society and the Swedish Intensive Care Society; and leadership or fiduciary roles in board, society, committee, or advocacy groups, paid or unpaid, with the American Physiological Society (Programming Committee); all outside the submitted work. B Langguth reports grants or contracts paid to their institution from the EU's Horizon 2020 Research and Innovation Programme, German Research Foundation, German Bundesministerium für Bildung und Forschung, EU, Bavarian-Czech University Association, and the Bavarian State; consulting fees from Schwabe, Neuromod, Sea Pharma, and Rovi; payment or honoraria for lectures, presentations, speakers bureaus, manuscript writing, or educational events from Schwabe, Neuromod, Medical Tribune, and Streamed Up; payment for expert testimony from the Bavarian State; support for attending meetings and/or travel from the EU UNITI project; has received funding from the EU's Horizon 2020 Research and Innovation Programme (Grant Agreement Number 848261) and Rovi; leadership or fiduciary roles in board, society, committee, or advocacy groups, unpaid, with Tinnitus Research Initiative, German Society for Brain Stimulation in Psychiatry, and Retex; stock or stock options with Sea Pharma; and receipt of equipment, materials, drugs, medical writing, gifts, or other services paid to their institution from Neurocare and Daymed; all outside the submitted work. H J Larson reports grants or contracts from Gates Foundation, GSK, and NIHR paid to LSHTM; consulting fees from ApiJect as strategy adviser and Gates MRI for vaccine confidence/acceptance research and advising; honoraria for lectures, presentations, speakers bureaus, manuscript writing, or educational events from Merrimon Honaray Lectureship; support for travel from the 2024 Asia Philanthropy Forum and the 2024 Science Technology Society Forum; and leadership or fiduciary roles in board, society, committee, or advocacy groups, unpaid, with PATH Board of Directors; all outside the submitted work. M Lee reports support for the present manuscript from the Ministry of Education of the Republic of Korea and the National Research Foundation of Korea (NRF-2023S1A3A2A05095298). D Lindholm reports stock or stock options with AstraZeneca during time of prior employment and other support from Astra Zeneca as a prior employee, all outside the submitted work. S Lorkowski reports grants or contracts paid to their institution from DSM-Firmenich (formerly DSM Nutritional Products); consulting fees from Danone, Novartis Pharma, and Swedish Orphan Biovitrum; payment or honoraria for lectures, presentations, speakers bureaus, manuscript writing, or educational events from AMARIN Germany, Amedes Holding, AMGEN, Berlin-Chemie, Boehringer Ingelheim Pharma, Daiichi Sankyo Deutschland, Danone, Hubert Burda Media Holding, Janssen-Cilag, Lilly Deutschland, Novartis Pharma, Novo Nordisk Pharma, Roche Pharma, Sanofi-Aventis, Swedish Orphan Biovitrum, and SYNLAB Holding Deutschland; support for attending meetings and/or travel from AMGEN; and participation on a data safety monitoring board or advisory board with AMGEN, Daiichi Sankyo Deutschland, Novartis Pharma, and Sanofi-Aventis; all outside the submitted work. H R Marateb reports grants or contracts paid to their institution from the Beatriu de Pinós post-doctoral program from the Office of the Secretary of Universities and Research from the Ministry of Business and Knowledge of the Government of Catalonia (programme number 2020 BP 00261), outside the submitted work. R J Maude reports support for the present manuscript from the Wellcome Trust (grant number 220211) as it provides core funding for Mahidol Oxford Tropical Medicine Research and contributes to his salary. He is required by Wellcome to acknowledge this grant in all publications. A-F Mentis reports grants or contracts from MilkSafe: a novel pipeline to enrich formula milk using omics technologies, which was research co-financed by the European Regional Development Fund of the EU and Greek national funds through the Operational Program Competitiveness, Entrepreneurship and Innovation, under the call RESEARCH–CREATE–INNOVATE (project code T2EDK-02222), as well as from ELIDEK (Hellenic Foundation for Research and Innovation, MIMS-860), both outside of the present manuscript; payment for expert testimony from Fondazione Capiplo, Italy, as an external peer-reviewer; leadership or fiduciary roles in board, society, committee, or advocacy groups, paid or unpaid, with *Systematic Reviews*, with *Annals of Epidemiology* as Editorial Board Member, and with *Translational Psychiatry* as Associate Editor; stock or stock options in a family winery; and other support as senior scientific officer at BGI Group, Shenzhen, China; all outside the submitted work. L Monasta reports support for the present manuscript from the Italian Ministry of Health (Ricerca Corrente 34/2017) via payments made to the Institute for Maternal and Child Health IRCCS Burlo Garofolo. F Mughal reports support for the present manuscript from an NIHR Doctoral Fellowship (award number 300957). A Ortiz reports grants from Sanofi paid to their institution IIS-FJD Universidad Autonoma de Madrid (UAM), and is Director of the Catedra Mundipharma-UAM of diabetic kidney disease and the Catedra Astrazeneca-UAM of chronic kidney disease and electrolytes; payment or honoraria for lectures, presentations, speakers bureaus, manuscript writing, or educational events from Advicciene, Astellas, Astrazeneca, Amicus, Amgen, Fresenius Medical Care, GSK, Bayer, Sanofi-Genzyme, Menarini, Kyowa Kirin, Alexion, Idorsia, Chiesi, Otsuka, Novo-Nordisk, and Vifor Fresenius Medical Care Renal Pharma; support for attending meetings and/travel from Advicciene, Astellas, Astrazeneca, Fresenius Medical Care, Boehringer-Ingelheim Bayer, Sanofi-Genzyme, Menarini, Chiesi, Otsuka, and Sysmex; participation on a data safety monitoring board or advisory board with Astellas, Astrazeneca, Boehringer-Ingelheim, Fresenius Medical Care, Bayer, Sanofi-Genzyme, Idorsia, Chiesi, Otsuka, Novo Nordisk, and Sysmex; and leadership or fiduciary roles in board, society, committee, or advocacy groups, unpaid, with the ERA Council and SOMANE; all outside the submitted work. R F Palma-Alvarez reports payment or honoraria for lectures, presentations, speakers bureaus, manuscript writing, or educational events from Angelini, Casen Recordati, Lundbeck, Neuraxpharm, Rubió, Servier, and Takeda; and support for attending meetings and/or travel from Angelini, Italfarmaco, Advanz Pharma, Takeda, and Lundbeck; all outside the submitted work. S K Panda reports support for the present manuscript via salary from Siksha ‘O’ Anusandhan (Deemed to be University). S K Panda also reports grants or contracts from the Science & Technology Department, Government of Odisha (Letter Number 3444/ST) outside the submitted work. R Passera reports being a member of the data safety monitoring board of the clinical trial Consolidation with ADCT-402 (loncastuximab tesirine) after immunochemotherapy: a phase II study in BTKi-treated/ineligible relapse/refractory mantle cell lymphoma (MCL) patients, sponsor Fondazione Italiana Linfomi, Alessandria; and leadership or fiduciary roles in board, society, committee, or advocacy groups as member of the EBMT Statistical Committee, European Society for Blood and Marrow Transplantation, Paris, France (unpaid), and as a past member (2020-2023, biostatistician) of the IRB/IEC Comitato Etico AO SS Antonio e Biagio Alessandria-ASL AL-VC (paid reimbursement of expenses); all outside the submitted work. L Ronfani reports support for the present manuscript from the Italian Ministry of Health (Ricerca Corrente 34/2017), via payments made to the Institute for Maternal and Child Health IRCCS Burlo Garofolo. N Scarmeas reports grants or contracts from Novo Nordisk as local Principal Investigator of a recruiting site for a multinational, multicenter industry sponsored phase III treatment trial for Alzheimer's disease (funding to institution); and participation on a data safety monitoring board or advisory board with an Albert Einstein College of Medicine NIH-funded study Multicultural healthy diet to reduce cognitive decline & AD risk (Chair of Data Safety Monitoring Board), and with a Primus-AD public-private-funded phase II study in Germany (Data Safety Monitoring Board Member; unpaid); all outside the submitted work. B M Schaarschmidt reports grants or contracts from Else Kröner-Fresenius Foundation, Deutsche Forschungsgemeinschaft, and PharmaCept; payment or honoraria for lectures, presentations, speakers bureaus, manuscript writing, or educational events from AstraZeneca; and support for attending meetings and/or travel from Bayer AG; all outside the submitted work. J P Silva reports support for the present manuscript from the Portuguese Foundation for Science and Technology via payment of salary (contract with reference 2021.01789.CEECIND/CP1662/CT0014). L M L R Silva reports a research contract with Instituto Politécnico of Guarda, Portugal outside the submitted work. J Sipilä reports grants or contracts from Siun Sote Foundation and Maire Taponen Foundation; payment or honoraria for lectures, presentations, speakers bureaus, manuscript writing, or educational events from Novartis; support for attending meetings and/or travel from Lundbeck; participation on a data safety monitoring board or advisory board with Boehringer-Ingelheim and Sandoz; and stock or stock options in Orion Corp; all outside the submitted work. J B Soriano reports support for the present manuscript via JBS and has received pharmaceutical company grants from 2020 to 2024 from Chiesi, GSK, Linde, and Novartis via Hospital Universitario de La Princesa. J B Soriano also participated in speaking activities, advisory committees, and consultancies from 2020 to 2024 sponsored by Air Liquide, Almirall, AstraZeneca, Boehringer Ingelheim, CHEST, Chiesi, CNPT, ERS, FTH, Gebro, Grifols, GSK, IHME, Laminar Pharma, Linde, Lipopharma, Menarini, Mundipharma, Novartis, OMS/WHO, Pfizer, ResApp, RiRL, ROVI, SEPAR, Seqirus, WHO EUR, Takeda, and Zambon. J B Soriano declares never, directly or indirectly, received any funding from tobacco manufacturers or their affiliates. J Sundström reports direct or indirect stock ownership in companies (Anagram Kommunikation, Sence Research, Symptoms Europe, MinForskning) providing services to companies and authorities in the health sector including Amgen, AstraZeneca, Bayer, Boehringer, Eli Lilly, Gilead, GSK, Göteborg University, Itrim, Ipsen, Janssen, Karolinska Institutet, LIF, Linköping University, Novo Nordisk, Parexel, Pfizer, Region Stockholm, Region Uppsala, Sanofi, STRAMA, Takeda, TLV, Uppsala University, Vifor Pharma, WeMind, all outside the submitted work. R Tabarés-Seisdedos reports grants or contracts from the Valencian Regional Government's Ministry of Education (PROMETEO/CIPROM/2022/58) and the Spanish Ministry of Science, Innovation and Universities (PID2021-129099OB-I00) outside the submitted work. D Trico reports support for attending meetings and/or travel from AstraZeneca, Eli Lilly, and Novo Nordisk; participation on a data safety monitoring board or advisory board with Amarin and Boehringer Ingelheim; and receipt of equipment, materials, drugs, medical writing, gifts or other services, paid to their institution, from PharmaNutra and Abbott; all outside the submitted work. S J Tromans reports grants or contracts paid to their institution, the University of Leicester, from NHS Digital, via the Department of Health and Social Care as part of the 2023 Adult Psychiatric Morbidity Survey team collecting epidemiological data on community-based adults living in England; and leadership or fiduciary roles in other board, society, committee or advocacy groups, paid or unpaid, with the Neurodevelopmental Psychiatry Special Interest Group and Psychiatry of Intellectual Disability Faculty at the Royal College of Psychiatrist as Academic Secretary, with *BMC Psychiatry, Advances in Autism, Advances in Mental Health and Intellectual Disability*, and *Progress in Neurology and Psychiatry* as Editorial Board Member, and for *Psychiatry of Intellectual Disability Across Cultures* (Oxford University Press) as Editor; all outside the submitted work. P Willeit reports consulting fees from Novartis Pharmaceuticals outside the submitted work. Y Yasufuku reports grants or contracts from Shionogi & Co paid to their institution, Osaka University, outside the submitted work. M Zielińska reports other financial or non-financial interests as an AstraZeneca employee outside the submitted work. E Zweck reports payment or honoraria for lectures, presentations, speakers bureaus, manuscript writing, or educational events from Abiomed outside the submitted work. All other authors declare no competing interests. The views expressed in this Article are those of the authors and not necessarily those of the NHS, NIHR, or the Department of Health and Social Care.

## References

[1] Roser M The Spanish flu: the global impact of the largest influenza pandemic in history. March 4, 2020. https://ourworldindata.org/spanish-flu-largest-influenza-pandemic-in-history.

[2] Crofts S, Stripe N Our population—where are we? How did we get here? Where are we going? How the UK's population has changed since the start of the 20th century. March 27, 2020. https://www.ons.gov.uk/peoplepopulationandcommunity/populationandmigration/populationestimates/articles/ourpopulationwherearewehowdidwegetherewherearewegoing/2020-03-27.

[3] Raleigh VS Trends in life expectancy in EU and other OECD countries. OECD Health Working Papers, no 108. Feb 28, 2019. https://www.oecd.org/en/publications/trends-in-life-expectancy-in-eu-and-other-oecd-countries_223159ab-en.html.

[4] Organisation for Economic Co-operation and Development, EU Health at a glance: Europe 2018: state of health in the EU Cycle. Nov 22, 2018. https://www.oecd.org/en/publications/health-at-a-glance-europe-2018_health_glance_eur-2018-en.html.

[5] Schöley J, Aburto JM, Kashnitsky I (2022). Life expectancy changes since COVID-19. Nat Hum Behav.

[6] Wang H, Paulson KR, Pease SA (2022). Estimating excess mortality due to the COVID-19 pandemic: a systematic analysis of COVID-19-related mortality, 2020–21. Lancet.

[7] Davis HE, McCorkell L, Vogel JM, Topol EJ (2023). Long COVID: major findings, mechanisms and recommendations. Nat Rev Microbiol.

[8] Public Health England Health profile for England: 2018. Sept 11, 2018. https://www.gov.uk/government/publications/health-profile-for-england-2018.

[9] Leon DA, Jdanov DA, Shkolnikov VM (2019). Trends in life expectancy and age-specific mortality in England and Wales, 1970–2016, in comparison with a set of 22 high-income countries: an analysis of vital statistics data. Lancet Public Health.

[10] Schumacher AE, Kyu HH, Aali A (2024). Global age-sex-specific mortality, life expectancy, and population estimates in 204 countries and territories and 811 subnational locations, 1950–2021, and the impact of the COVID-19 pandemic: a comprehensive demographic analysis for the Global Burden of Disease Study 2021. Lancet.

[11] Brauer M, Roth GA, Aravkin AY (2024). Global burden and strength of evidence for 88 risk factors in 204 countries and 811 subnational locations, 1990–2021: a systematic analysis for the Global Burden of Disease Study 2021. Lancet.

[12] Naghavi M, Ong KL, Aali A (2024). Global burden of 288 causes of death and life expectancy decomposition in 204 countries and territories and 811 subnational locations, 1990–2021: a systematic analysis for the Global Burden of Disease Study 2021. Lancet.

[13] Vollset SE, Ababneh HS, Abate YH (2024). Burden of disease scenarios for 204 countries and territories, 2022–2050: a forecasting analysis for the Global Burden of Disease Study 2021. Lancet.

[14] Weir HK, Thun MJ, Hankey BF (2003). Annual report to the nation on the status of cancer, 1975–2000, featuring the uses of surveillance data for cancer prevention and control. J Natl Cancer Inst.

[15] National Cancer Institute Joinpoint Trend Analysis Software 2022. https://surveillance.cancer.gov/joinpoint/.

[16] Zheng P, Afshin A, Biryukov S (2022). The Burden of Proof studies: assessing the evidence of risk. Nat Med.

[17] Wickham H (2016). ggplot2: elegant graphics for data analysis.

[18] The R Foundation The R project for statistical computing. https://www.R-project.org/.

[19] Modig K, Andersson T, Vaupel J, Rau R, Ahlbom A (2017). How long do centenarians survive? Life expectancy and maximum lifespan. J Intern Med.

[20] Global Burden of Disease Collaborative Network Global Burden of Disease Study 2019 (GBD 2019) reference life table. 2021. https://ghdx.healthdata.org/record/ihme-data/global-burden-disease-study-2019-gbd-2019-reference-life-table.

[21] Bajekal M, Scholes S, Love H (2012). Analysing recent socioeconomic trends in coronary heart disease mortality in England, 2000–2007: a population modelling study. PLoS Med.

[22] Unal B, Sözmen K, Arık H (2013). Explaining the decline in coronary heart disease mortality in Turkey between 1995 and 2008. BMC Public Health.

[23] Petersen J, Benzeval M (2016). Untreated hypertension in the UK household population—who are missed by the general health checks?. Prev Med Rep.

[24] Monasta L, Abbafati C, Logroscino G (2019). Italy's health performance, 1990–2017: findings from the Global Burden of Disease Study 2017. Lancet Public Health.

[25] Institut National du Cancer 2021–2030 France ten-year cancer-control strategy. 2021–2025 roadmap. January, 2022. http://sante.gouv.fr/IMG/pdf/2021-2030_france_ten-year_cancer-control_strategy_2021-2025_roadmap.pdf.

[26] Minister of Social Affairs and Public Health National Cancer Plan 2008–2010. March 10, 2008. http://www.epaac.eu/from_heidi_wiki/Belgium_National_Cancer_Plan_2008-2010_English.pdf.

[27] Norwegian Ministry of Health and Care Services Together—against cancer. National Cancer Strategy 2013–2017. http://www.walkazrakiem.pl/sites/default/files/library/files/norwegian_national_cancer_strategy_2013.pdf.

[28] Swanton RH (2006). The National Service Framework: six years on. Heart.

[29] Gilmore AB, Fabbri A, Baum F (2023). Defining and conceptualising the commercial determinants of health. Lancet.

[30] Anderson P, Stockwell T, Natera G, Kaner E (2024). Minimum unit pricing for alcohol saves lives, so why is it not implemented more widely?. BMJ.

[31] Wyper GMA, Mackay DF, Fraser C (2023). Evaluating the impact of alcohol minimum unit pricing on deaths and hospitalisations in Scotland: a controlled interrupted time series study. Lancet.

[32] Probst C, Kilian C, Sanchez S, Lange S, Rehm J (2020). The role of alcohol use and drinking patterns in socioeconomic inequalities in mortality: a systematic review. Lancet Public Health.

[33] Murray CJL, Aravkin AY, Zheng P (2020). Global burden of 87 risk factors in 204 countries and territories, 1990–2019: a systematic analysis for the Global Burden of Disease Study 2019. Lancet.

[34] Institute for Health Metrics and Evaluation Dietary risks—level 2 risk. http://www.healthdata.org/research-analysis/diseases-injuries-risks/factsheets/2021-dietary-risks-level-2-risk.

[35] Bloomfield HE, Koeller E, Greer N, MacDonald R, Kane R, Wilt TJ (2016). Effects on health outcomes of a Mediterranean diet with no restriction on fat intake: a systematic review and meta-analysis. Ann Intern Med.

[36] Guasch-Ferré M, Salas-Salvadó J, Ros E (2017). The PREDIMED trial, Mediterranean diet and health outcomes: how strong is the evidence?. Nutr Metab Cardiovasc Dis.

[37] Willett W, Rockström J, Loken B (2019). Food in the Anthropocene: the EAT–*Lancet* Commission on healthy diets from sustainable food systems. Lancet.

[38] Clarsen B, Nylenna M, Klitkou ST (2022). Changes in life expectancy and disease burden in Norway, 1990–2019: an analysis of the Global Burden of Disease Study 2019. Lancet Public Health.

[39] WHO Regional Office for Europe Evaluation of the Norwegian nutrition policy with focus on the Action Plan on Nutrition 2007–2011. 2013. https://iris.who.int/handle/10665/350603.

[40] The Scottish Public Health Observatory Diet and nutrition: introduction and policy context. https://www.scotpho.org.uk/risk-factors/diet-and-nutrition/introduction-and-policy-context/.

[41] Warburton DER, Bredin SSD (2019). Health benefits of physical activity: a strengths-based approach. J Clin Med.

[42] Marmot M, Allen J, Boyce T, Goldblatt P, Morrison J Health equity in England: the Marmot Review 10 years on. 2020. http://www.instituteofhealthequity.org/the-marmot-review-10-years-on.

[43] Wilkinson RG, Pickett KE (2006). Income inequality and population health: a review and explanation of the evidence. Soc Sci Med.

[44] Marmot M, Bobak M (2000). International comparators and poverty and health in Europe. BMJ.

[45] Toffolutti V, Suhrcke M (2019). Does austerity really kill?. Econ Hum Biol.

[46] Hiam L, Harrison D, McKee M, Dorling D (2018). Why is life expectancy in England and Wales ‘stalling’?. J Epidemiol Community Health.

[47] The World Bank Current health expenditure per capita. https://data.worldbank.org/indicator/SH.XPD.CHEX.PC.CD.

[48] Stuckler D, Reeves A, Loopstra R, Karanikolos M, McKee M (2017). Austerity and health: the impact in the UK and Europe. Eur J Public Health.

[49] Vodden A, Holdroyd I, Bentley C (2023). Evaluation of the national governmental efforts between 1997 and 2010 in reducing health inequalities in England. Public Health.

[50] Alexiou A, Fahy K, Mason K (2021). Local government funding and life expectancy in England: a longitudinal ecological study. Lancet Public Health.

[51] McCartney G, McMaster R, Popham F, Dundas R, Walsh D (2022). Is austerity a cause of slower improvements in mortality in high-income countries? A panel analysis. Soc Sci Med.

[52] Parkinson J, Minton J, Lewsey J, Bouttell J, McCartney G (2018). Drug-related deaths in Scotland 1979–2013: evidence of a vulnerable cohort of young men living in deprived areas. BMC Public Health.

[53] Parkinson J, Minton J, Lewsey J, Bouttell J, McCartney G (2017). Recent cohort effects in suicide in Scotland: a legacy of the 1980s?. J Epidemiol Community Health.

[54] Minton J, Green M, McCartney G, Shaw R, Vanderbloemen L, Pickett K (2017). Two cheers for a small giant? Why we need better ways of seeing data: a commentary on: ‘Rising morbidity and mortality in midlife among White non-Hispanic Americans in the 21st century’. Int J Epidemiol.

[55] McCartney G, Bouttell J, Craig N (2016). Explaining trends in alcohol-related harms in Scotland, 1991–2011 (I): the role of incomes, effects of socio-economic and political adversity and demographic change. Public Health.

[56] Minton J, Shaw R, Green MA, Vanderbloemen L, Popham F, McCartney G (2017). Visualising and quantifying ‘excess deaths’ in Scotland compared with the rest of the UK and the rest of Western Europe. J Epidemiol Community Health.

[57] Catillon M, Cutler D, Getzen T Two hundred years of health and medical care: the importance of medical care for life expectancy gains. Working paper 25330. December, 2018. https://www.nber.org/papers/w25330.

[58] Eurostat Description of the Eurostat method for the calculation of the life expectancies at all ages. https://ec.europa.eu/eurostat/cache/metadata/Annexes/demo_mor_esms_an_1.pdf.

[59] Wang H, Abbas KM, Abbasifard M (2020). Global age-sex-specific fertility, mortality, healthy life expectancy (HALE), and population estimates in 204 countries and territories, 1950–2019: a comprehensive demographic analysis for the Global Burden of Disease Study 2019. Lancet.

[60] Evans A Changing trends in mortality: an international comparison: 2000 to 2016. Aug 7, 2018. https://www.ons.gov.uk/peoplepopulationandcommunity/birthsdeathsandmarriages/lifeexpectancies/articles/changingtrendsinmortalityaninternationalcomparison/2000to2016.

[61] Organisation for Economic Co-operation and Development Is the last mile the longest? Economic gains from gender equality in Nordic countries. May 14, 2018. https://www.oecd.org/en/publications/is-the-last-mile-the-longest-economic-gains-from-gender-equality-in-nordic-countries_9789264300040-en.html.

[62] WHO What you need to know about Health in All Policies. June 23, 2015. https://www.who.int/publications/m/item/what-you-need-to-know-about-health-in-all-policies–key-messages.pdf?sfvrsn=a4982d1_1.

[63] Greszczuk C Implementing Health in All Policies: lessons from around the world. Aug 28, 2019. https://www.health.org.uk/reports-and-analysis/reports/implementing-health-in-all-policies.

[64] Loopstra R, McKee M, Katikireddi SV, Taylor-Robinson D, Barr B, Stuckler D (2016). Austerity and old-age mortality in England: a longitudinal cross-local area analysis, 2007-2013. J R Soc Med.

[65] Watkins J, Wulaningsih W, Da Zhou C (2017). Effects of health and social care spending constraints on mortality in England: a time trend analysis. BMJ Open.

[66] Whitty CS, Atherton F, McBride M Technical report on the COVID-19 pandemic in the UK. A technical report for future UK Chief Medical Officers, Government Chief Scientific Advisers, National Medical Directors and public health leaders in a pandemic. Dec 1, 2022. https://covid19.public-inquiry.uk/wp-content/uploads/2023/07/24103332/INQ000087225.pdf.

[67] Xie Y, Xu E, Bowe B, Al-Aly Z (2022). Long-term cardiovascular outcomes of COVID-19. Nat Med.

