# Supplementary material for: Changing life expectancy in European countries 1990–2021: a subanalysis of causes and risk factors from the Global Burden of Disease Study 2021
Source: Lancet Public Health. 2025 Feb 18;10(3):e172–88. doi: 10.1016/S2468-2667(25)00009-X (PMC11876102; doi:10.1016/S2468-2667(25)00009-X)
Supplement: Supplementary appendix 1 [file mmc1.pdf]

THE LANCET
Public Health

Supplementary appendix 1

This appendix formed part of the original submission and has been peer reviewed. We post it as supplied by the authors.

Supplement to: GBD 2021 Europe Life Expectancy Collaborators. Changing life expectancy in European countries 1990–2021: a subanalysis of causes and risk factors from the Global Burden of Disease Study 2021. *Lancet Public Health* 2025; published online Feb 18. https://doi.org/10.1016/S2468-2667(25)00009-X.

***Appendix Figure* 1: Mean annual changes in life expectancy at birth for both sexes combined, by country and cause of death from 1990 to 2011 and 2011 to 2019, with a standardised baseline year for each time period, ordered by 2019 life expectancy**

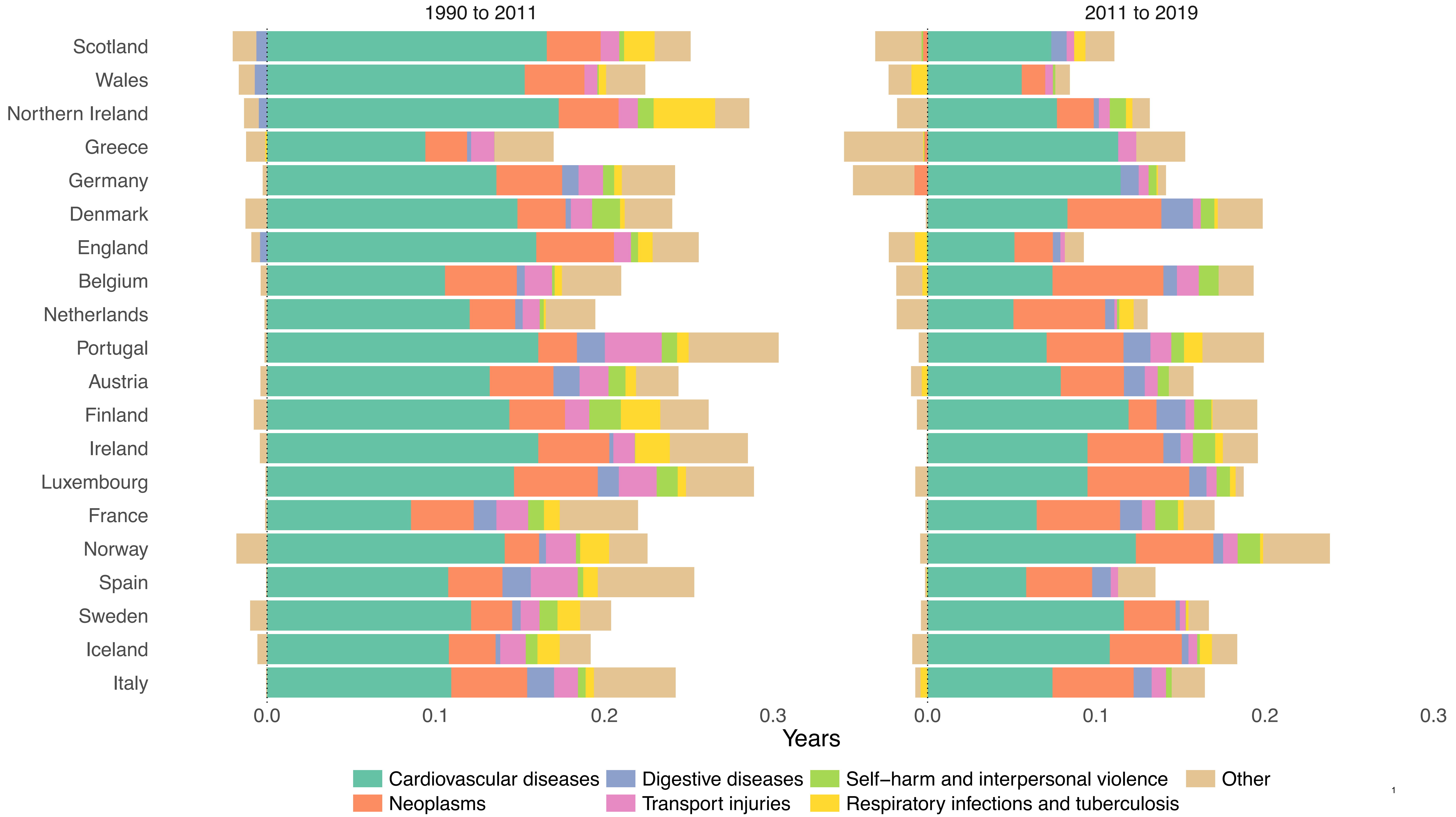

Appendix Figure 2: Age−standardised summary exposure values for both sexes combined on a 0−100 scale (where 0 means minimum risk and 100 means the entire population is at maximum risk) for major risk factors for cardiovascular disease and neoplasms, from 1990 to 2019

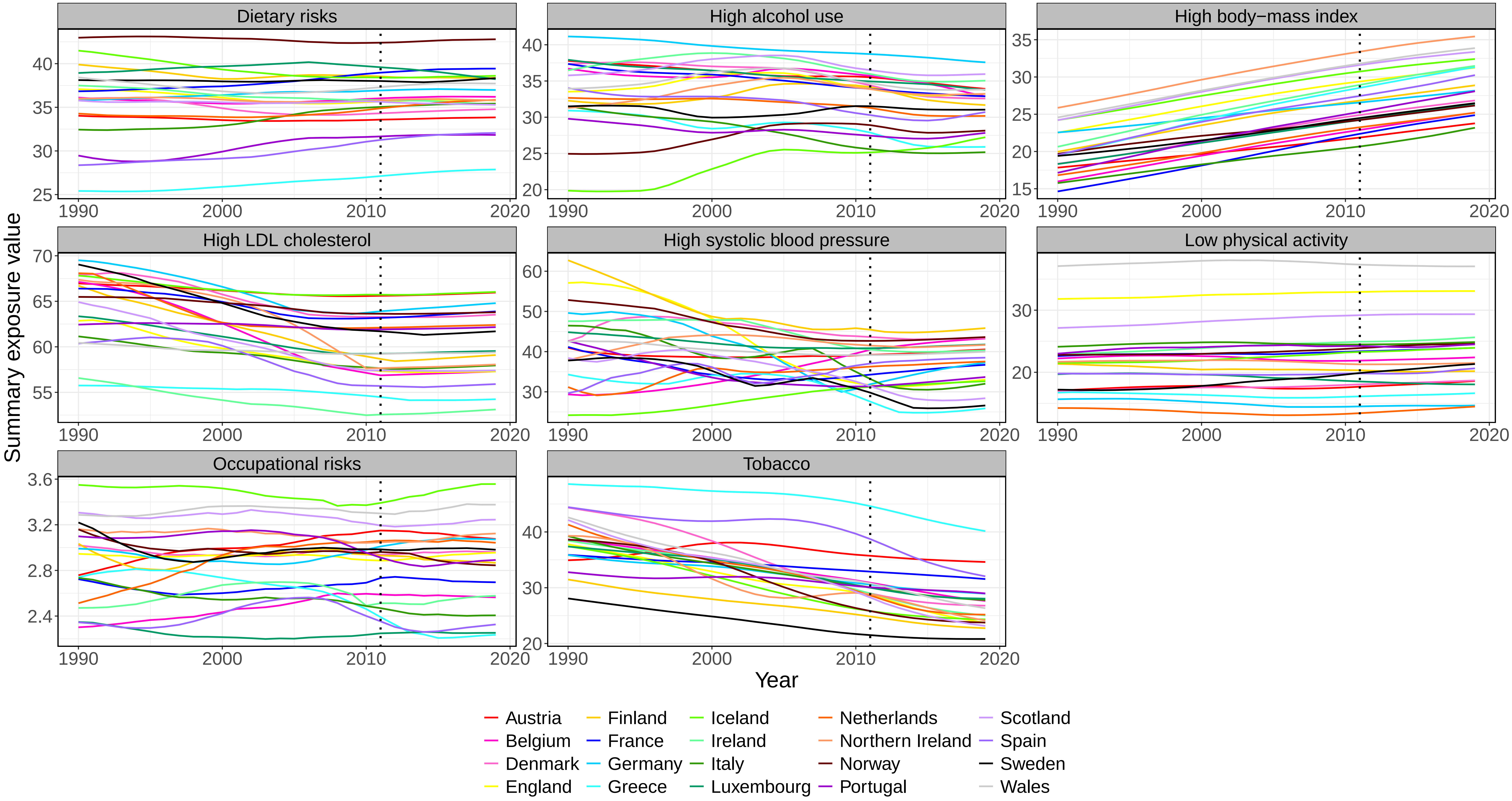

Appendix Figure 3: **Mean annual changes in life expectancy in years and mean annual change in age−standardised summary exposure values, for major risk factors associated with cardiovascular diseases and neoplasm from 1990 to 2011 and 2011 to 2019, for both sexes combined**

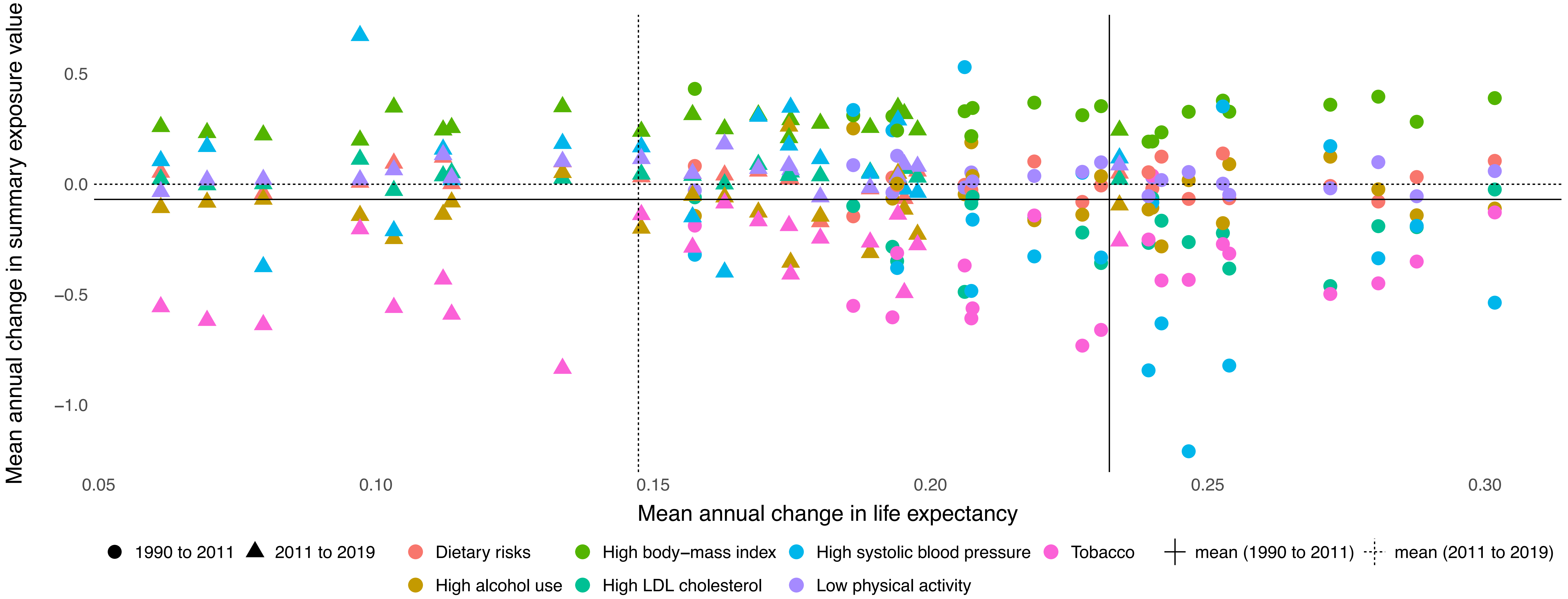

Each datapoint represents a single country with circular points for the time period 1990 to 2011 and triangular points for 2011 to 2019. Each data point is coloured according to risk factor. The crossed axis lines show the mean values across all countries for 1990 to 2011 (solid lines) and 2011 to 2019 (dashed lines) for mean annual change in summary exposure value (SEV) (y-axis) and mean annual change in life expectancy (x-axis). The mean change in SEV in 2011-19 was set at zero as the baseline. Occupational risks not presented on figure due to no perceivable changes

Definitions of risk factors for cardiovascular disease and neoplasms

GBD Level 2 Risk Factor	Definition
Air pollution	Air pollution includes ambient particulate matter pollution (PM2.5), household air pollution from the use of solid fuels for cooking (HAP), ambient ozone pollution, and nitrogen dioxide pollution
Dietary risks	Dietary risks are an aggregate risk factor for all of the GBD dietary risks, including diet low in whole grains, fruit, fibre, legumes, nuts and seeds, seafood omega-3 fatty acids, omega-6 polyunsaturated fatty acids, vegetables, milk, and calcium; and diet high in sodium, trans fatty acids, red meat, processed meat, and sugar-sweetened beverages
High alcohol use	Alcohol consumption in excess of the region-, age-, sex-, and year-specific theoretical minimum risk exposure level (TMREL). Current drinkers are defined as individuals consuming at least one alcoholic beverage in the past year. We estimate the level of alcohol exposure for current drinkers with the reported average grams of pure alcohol consumed per day (g/day)
High BMI	High BMI for adults (ages 20 and older) is defined as BMI greater than 20–23 kg/m2 . High BMI for children (ages 2–19) is defined as being overweight or obese based on International Obesity Task Force standards
High fasting plasma glucose	High fasting plasma glucose is defined as fasting plasma glucose greater than 4·9–5·3 mmol/L
High LDL cholesterol	We estimated blood concentration of LDL-c in units of mmol/L. We used a TMREL with a uniform distribution between 0·9 and 1·4 mmol/L
High systolic blood pressure	We estimated brachial SBP in mm Hg. We used a TMREL of SBP ranging from 105 to 115 mm Hg
Kidney dysfunction	Kidney dysfunction is defined as estimated glomerular filtration rate (eGFR) <60 ml/min/1·73 m2 or albumin to creatinine ratio (ACR) ≥30 mg/g. The theoretical minimum risk exposure level value is ACR <30 mg/g and eGFR ≥60 ml/min/1·73 m2
Low physical activity	Low physical activity was measured in total metabolic equivalent (MET)-minutes per week and was defined as objectively measured, average weekly physical activity (at work, home, transport-related, and recreational) of less than 3600–4400 MET-minutes per week
Non-optimal temperature	Non-optimal temperature is an aggregate of the burden attributable to low and high temperatures. Heat and cold effects relate to effects above and below the TMREL. The population-weighted mean TMREL is 25·6°C

Occupational risks	Occupational risks constitute an aggregation of all individual occupational risks modelled as Level 3 risk factors. These include occupational injuries; ergonomic factors; and occupational exposure to particulate matter, fumes and gases, carcinogens, noise, and asthmagens
Other environmental risks	Other environmental risks includes exposure to residential radon and both acute and chronic exposure to lead
Tobacco smoke	Tobacco includes tobacco smoking, chewing tobacco use, and secondhand smoke exposure
Unsafe sex	Unsafe sex is defined as the risk of disease due to sexual transmission. Unsafe sex includes 100% of cervical cancer and STIs apart from those congenitally acquired, and a fraction of HIV based on data reporting the proportion of HIV incidence through sexual transmission

All GBD risk factors are defined in the IHME factsheets for risk factors, available from:

https://www.healthdata.org/research-analysis/diseases-injuries-risks/factsheets

Last accessed 25 January 2025

Calculation of mean annual change in life expectancy

The mean annual changes in life expectancy for each country were estimated using data from the first and last years' life expectancy within each time interval.

The mean annual change in life expectancy (ΔLE) was calculated as:

$$\Delta LE = \frac{LE_{t_2} - LE_{t_1}}{(t_2 - t_1)}$$

where t_1 = is the first year of each time interval, and

t_2 = is the last year of each time interval

Identification of the leading causes that contributed to life expectancy

In figures 2-4 in the main paper, the life expectancy decomposition figures visualise causes that contributed to changes in life expectancy for each country individually. However, the identification of the top 5 causes was based on life expectancy decomposition by cause across all selected countries combined. Pooled decomposition values for the combined group of countries by cause were not available, so the top 5 causes were identified by taking a mean of all selected countries' decomposition values for each cause and selecting the 5 that were the biggest contributors to changes in life expectancy. Respiratory infections and tuberculosis were additionally included across all time intervals to allow for comparisons before and after the COVID-19 pandemic.

For the first two time intervals (1990 to 2011 and 2011 to 2019), the identification of the top 5 causes was based on the decomposition data for 2011 to 2019, to allow for comparison of how different causes contributed to changes in life expectancy across the two time intervals prior to disruptions of the COVID-19 pandemic. For the latter time interval, covering the pandemic, the top 5 causes were based on the 2019 to 2021 decomposition values.

Estimation of uncertainty intervals

All estimates were calculated as the mean of 1000 draws, and 95% uncertainty limits were the 2.5th and 97.5th percentile of all draws.
