# Supplementary material for: Changing life expectancy in European countries 1990–2021: a subanalysis of causes and risk factors from the Global Burden of Disease Study 2021
Source: Lancet Public Health. 2025 Feb 18;10(3):e172–88. doi: 10.1016/S2468-2667(25)00009-X (PMC11876102; doi:10.1016/S2468-2667(25)00009-X)
Supplement: Supplementary appendix 2 [file mmc2.pdf]

THE LANCET
Public Health

Supplementary appendix 2

This appendix formed part of the original submission and has been peer reviewed. We post it as supplied by the authors.

Supplement to: GBD 2021 Europe Life Expectancy Collaborators. Changing life expectancy in European countries 1990–2021: a subanalysis of causes and risk factors from the Global Burden of Disease Study 2021. *Lancet Public Health* 2025; published online Feb 18. https://doi.org/10.1016/S2468-2667(25)00009-X.

Appendix 2: Authorship appendix to "Changing life expectancy in European countries 1990–2021: a subanalysis of causes and risk factors from the Global Burden of Disease Study 2021"

This appendix provides further authorship detail for "Changing life expectancy in European countries 1990–2021: a subanalysis of causes and risk factors from the Global Burden of Disease Study 2021"

Table of Contents

GBD 2021 Europe Life Expectancy Collaborators **2**

Affiliations **5**

Authors' Contributions **23**

- Providing data or critical feedback on data sources 23
- Developing methods or computational machinery 24
- Providing critical feedback on methods or results 24
- Drafting the work or revising it critically for important intellectual content 26
- Managing the estimation or publications process 28

GBD 2021 Europe Life Expectancy Collaborators

Full Name

Nicholas Steel,* Clarissa Maria Mercedes Bauer-Staeb,† John A Ford,‡ Cristiana Abbafati, Mohammed Altigani Abdalla, Atef Abdelkader, Parsa Abdi, Roberto Ariel Abeldaño Zuñiga, Olugbenga Olusola Abiodun, Hassan Abolhassani, Eman Abu-Gharbieh, Hana J Abukhadijah, Ahmed Abu-Zaid, Isaac Yeboah Addo, Giovanni Addolorato, Victor Adekanmbi, Juliana Bunmi Adetunji, Temitayo Esther Adeyeoluwa, Emilie E Agardh, Williams Agyemang-Duah, Danish Ahmad, Anisuddin Ahmed, Ayman Ahmed, Syed Anees Ahmed, Karolina Akinosoglou, Mohammed Ahmed Akkaif, Salah Al Awaidy, Syed Mahfuz Al Hasan, Omar Ali Mohammed Al Zaabi, Robert W Aldridge, Abdelazeem M Algammal, Adel Ali Saeed Al-Gheethi, Abid Ali, Mohammed Usman Ali, Syed Shujait Ali, Waad Ali, Gianfranco Alicandro, Sheikh Mohammad Alif, Adel Al-Jumaily, Peter Allebeck, Ahmad Alrawashdeh, Rami H Al-Rifai, Mohammed A Alsabri, Najim Z Alshahrani, Deborah Oyine Aluh, Mohammad Al-Wardat, Walid A Al-Zyoud, Sohrab Amiri, Deanna Anderlini, Catalina Liliana Andrei, Abhishek Anil, Saeid Anvari, Anayochukwu Edward Anyasodor, Seth Christopher Yaw Appiah, Michele Aquilano, Jalal Arabloo, Mosab Arafat, Demelash Areda, Abdulfatai Aremu, Keivan Armani, Benedetta Armocida, Johan Ärnlöv, Muhammad Asaduzzaman, Thomas Astell-Burt, Avinash Aujayeb, Marcel Ausloos, Sina Azadnajafabad, Shahkaar Aziz, Ahmed Y Azzam, Giridhara Rathnaiah Babu, Andreea Corina Badache, Ashish D Badiye, Saeed Bahramian, Atif Amin Baig, Jennifer L Baker, Hansi Bansal, Till Winfried Bärnighausen, Mark Thomaz Ugliara Barone, Amadou Barrow, Sandra Barteit, Shahid Bashir, Hameed Akande Bashiru, João Diogo Basso, Mohammad-Mahdi Bastan, Sanjay Basu, Kavita Batra, Matteo Bauckneht, Bernhard T Baune, Massimiliano Beghi, Maryam Beiranvand, Yannick Béjot, Michelle L Bell, Olorunjuwon Omolaja Bello, Luis Belo, Apostolos Beloukas, Alice A Beneke, Paulo J G Bettencourt, Akshaya Srikanth Bhagavathula, Neeraj Bhala, Sonu Bhaskar, Francesca Bisulli, Tone Bjørge, Aadam Olalekan Bodunrin, Alejandro Botero Carvajal, Souad Bouaoud, Carol Brayne, Hermann Brenner, Adam D M Briggs, Nikolay Ivanovich Briko, Raffaele Bugiardini, Danilo Buonsenso, Reinhard Busse, Yasser Bustanji, Florentino Luciano Caetano dos Santos, Mehtap Çakmak Barsbay, Angelo Capodici, Giulia Carreras, Andrea Carugno, Felix Carvalho, Márcia Carvalho, Joao Mauricio Castaldelli-Maia, Giulio Castelpietra, Alberico L Catapano, Maria Sofia Cattaruzza, Luca Cegolon, Edina Cenko, Ester Cerin, Sonia Cerrai, Anis Ahmad Chaudhary, Bryan Chong, Sonali Gajanan Choudhari, Dinh-Toi Chu, Isaac Sunday Chukwu, Sheng-Chia Chung, Iolanda Cioffi, Joao Conde, Samuele Cortese, Rosa A S Couto, Michael H Criqui, Natalia Cruz-Martins, Omid Dadras, Mary Anne Teresa Dallat, Emanuele D'Amico, Lucio D'Anna, Samuel Demissie Darcho, Paul I Dargan, Saswati Das, Alejandro de la Torre-Luque, Cristian Del Bo', Andreas K Demetriades, Nikolaos Dervenis, Brecht Devleesschauwer, Arkadeep Dhali, Kuldeep Dhama, Mostafa Dianatinasab, Michael J Diaz, Deepa Dongarwar, Mario D'Oria, Ojas Prakashbhai Doshi, Robert Kokou Dowou, Senbagam Duraisamy, Oyewole Christopher Durojaiye, Arkadiusz Marian Dziedzic, David Edvardsson, Kristina Edvardsson, Terje Andreas Eikemo, Michael Ekholuenetale, Temitope Cyrus Ekundayo, Rabie Adel El Arab, Frank J Elgar, Muhammed Elhadi, Chadi Eltaha, Francesco Esposito, Natalia Fabin, Adeniyi Francis Fagbamigbe, Omotayo Francis Fagbule, Aliasghar Fakhri-Demeshghieh, Luca Falzone, Carla Sofia e Sá Farinha, Pawan Sirwan Faris, Folorunso Oludayo Fasina, Patrick Fazeli, Timur Fazylov, Alireza Feizkhah, Ginenus Fekadu, Xiaoqi Feng, Seyed-Mohammad Fereshtehnejad, Daniela Ferrante, Pietro Ferrara, Nuno Ferreira, Getahun Fetensa, Florian Fischer, Marco Fonzo, Arianna Fornari, Daniela Fortuna, Celia Fortuna Rodrigues, Matteo Foschi, Sebastian S Fox, Alberto Freitas, Takeshi Fukumoto, Muktar A Gadanya, Silvano Gallus, Lucia Galluzzo, Balasankar Ganesan, Mohammad Arfat Ganiyani, Xiang Gao, MA Garcia-Gordillo, Federica Gazzelloni, Miglas Welay Gebregergis, Teferi Gebru Gebremeskel, Delaram J Ghadimi,

Khalid Yaser Ghailan, Nermin Ghith, Ehsan Gholami, Alessandro Gialluisi, Paramjit Singh Gill, Tara Gillam, Giorgia Giussani, James C Glasbey, Scott D Glenn, Laszlo Göbölös, Mohamad Goldust, Mahaveer Golechha, Pouya Goleij, Davide Golinelli, Giuseppe Gorini, Simon Matthew Graham, Robert Griebler, Ashna Grover, Stefano Guicciardi, Sasidhar Gunturu, Vijai Kumar Gupta, Roberth Steven Gutiérrez-Murillo, Awoke Derbie Habteyohannes, Nils Haep, Nguyen Hai Nam, Sebastian Haller, Rifat Hamoudi, Senad Handanagic, Josep Maria Haro, Hamidreza Hasani, Md Saquib Hasnain, Rasmus J Havmoeller, Simon I Hay, Jeffrey J Hebert, Behzad Heibati, Henk B M Hilderink, Yuta Hiraike, Nguyen Quoc Hoan, Mehdi Hosseinzadeh, Sorin Hostiuc, Hanno Hoven, Chengxi Hu, Junjie Huang, Andrew Hughes, Michael Hultström, Kiavash Hushmandi, Javid Hussain, M Azhar Hussain, Adalia Ikiroma, Arit Inok, Md Rabiul Islam, Sheikh Mohammed Shariful Islam, Gaetano Isola, Mahalaxmi Iyer, Louis Jacob, Haitham Jahrami, Ammar Abdulrahman Jairoun, Sanobar Jaka, Mihajlo Jakovljevic, Talha Jawaid, Bijay Mukesh Jeswani, Jost B Jonas, Charity Ehimwenma Joshua, Billingsley Kaambwa, Zubair Kabir, Dler H Hussein Kadir, Rajesh Kamath, Kehinde Kazeem Kanmodi, Neeti Kapoor, Paschalis Karakasis, Marina Karanikolos, Ibraheem M Karaye, Joonas H Kauppila, Sina Kazemian, Emmanuelle Kesse-Guyot, Faham Khamesipour, Ajmal Khan, Shaghayegh Khanmohammadi, Khaled Khatab, Moawiah Mohammad Khatatbeh, Moein Khormali, Atulya Aman Khosla, Majid Khosravi, Mahmood Khosrowjerdi, Jagdish Khubchandani, Kwanghyun Kim, Min Seo Kim, Adnan Kisa, Sezer Kisa, Ann Kristin Skrindo Knudsen, Gerbrand Koren, Md Abdul Kuddus, Ilari Kuitunen, Mukhtar Kulimbet, Rakesh Kumar, Setor K Kunutsor, Om P Kurmi, Dian Kusuma, Ville Kytö, Carlo La Vecchia, Hanpeng Lai, Tea Lallukka, Francesco Lanfranchi, Berthold Langguth, Ariane Laplante-Lévesque, Heidi Jane Larson, Anders O Larsson, Munjae Lee, Paul H Lee, Seung Won Lee, Wei-Chen Lee, Daniel Lindholm, Christine Linehan, Xuefeng Liu, Erand Llanaj, José Francisco López-Gil, Stefan Lorkowski, Giancarlo Lucchetti, Alessandra Lugo, Raimundas Lunevicius, Lisha Luo, Hawraz Ibrahim M Amin, Zheng Feei Ma, Nikolaos Machairas, Monika Machoy, Kashish Malhotra, Ahmad Azam Malik, Ali Mansour, Emmanuel Manu, Hamid Reza Marateb, Daniela Martini, Miquel Martorell, Roy Rillera Marzo, Yasith Mathangasinghe, Medha Mathur, Fernanda Penido Matozinhos, Richard James Maude, Andrea Maugeri, Juergen May, Mahsa Mayeli, Mohsen Mazidi, Martin McKee, Enkeleint A Mechili, Sepideh Mehravar, Tesfahun Mekene Meto, Hadush Negash Meles, Alexios-Fotios A Mentis, Atte Meretoja, Tuomo J Meretoja, Sachith Mettananda, Georgia Micha, Irmina Maria Michalek, Ted R Miller, Giuseppe Minervini, Antonio Mirijello, Gabriele Mocciaro, Atousa Moghadam Fard, Jama Mohamed, Nouh Saad Mohamed, Abdollah Mohammadian-Hafshejani, Shafiu Mohammed, Lorenzo Monasta, Stefania Mondello, Mohammad Ali Moni, Paula Moraga, Lidia Morawska, Tilahun Belete Mossie, Rohith Motappa, Sumaira Mubarik, Lorenzo Muccioli, Ulrich Otto Mueller, Faraz Mughal, Francesk Mulita, Daniel Munblit, Yanjinlkham Munkhsaikhan, Christopher J L Murray, Mohsen Naghavi, Pirouz Naghavi, Ganesh R Naik, Soroush Najdaghi, Atta Abbas Naqvi, Delaram Narimani Davani, Gustavo G Nascimento, Abdallah Y Naser, Abdulqadir J Nashwan, Javaid Nauman, Samidi Nirasha Kumari Navaratna, Athare Nazri-Panjaki, Chakib Nejjari, Evangelia Nena, Henok Biresaw Netsere, Anh Hoang Nguyen, Phat Tuan Nguyen, Van Thanh Nguyen, Lawrence Achilles Nnyanzi, Syed Toukir Ahmed Noor, Mehran Nouri, Fred Nugen, Mario Cesare Nurchis, Ogochukwu Janet Nzoputam, Bogdan Oancea, Martin James O'Donnell, Michael Safo Oduro, Oluwaseun Adeolu Ogundijo, Ropo Ebenezer Ogunsakin, Sylvester Reuben Okeke, Osaretin Christabel Okonji, Andrew T Olagunju, Susan Oliver, Isaac Iyinoluwa Olufadewa, Alberto Ortiz, Mayowa O Owolabi, Mahesh Padukudru P A, Jagadish Rao Padubidri, Raul Felipe Felipe Palma-Alvarez, Sujogya Kumar Panda, Songhomitra Panda-Jonas, Georgios D Panos, Leonidas D Panos, Ioannis Pantazopoulos, Shahina Pardhan, Romil R Parikh, Roberto Passera, Shankargouda Patil, Dimitrios Patoulias, Shrikant Pawar, Umberto Pensato, Gavin Pereira, Norberto Perico, Simone Perna, Fanny Emily

Petermann-Rocha, Hoang Nhat Pham, Anil K Philip, Daniela Pierannunzio, Manon Pigeolet, Enrico Pisoni, Dimitri Poddighe, Ramesh Poluru, Maarten J Postma, Jalandhar Pradhan, Elisabetta Pupillo, Jagadeesh Puvvula, Alberto Raggi, Mosiur Rahman, Muhammad Aziz Rahman, Diego Raimondo, Ivano Raimondo, Shakthi Kumaran Ramasamy, Sheena Ramazanu, Rishabh Kumar Rana, Sowmya J Rao, Davide Rasella, Ahmed Mustafa Rashid, Santosh Kumar Rauniyar, Ilari Rautalin, David Laith Rawaf, Salman Rawaf, Murali Mohan Rama Krishna Reddy, Elrashdy M Moustafa Mohamed Redwan, Lennart Reifels, Giuseppe Remuzzi, Mohsen Rezaeian, Ana Isabel Ribeiro, Anupa Rijal, Jefferson Antonio Buendia Rodriguez, Michele Romoli, Luca Ronfani, Kevin T Root, Himanshu Sekhar Rout, Nitai Roy, Michele Russo, Aly M A Saad, Cameron John Sabet, Mamta Sachdeva Dhingra, Umar Saeed, Mehdi Safari, Mahdi Safdarian, Mohamed A Saleh, Mohammed Z Y Salem, Giovanni A Salum, Vijaya Paul Samuel, Abdallah M Samy, Aswini Saravanan, Babak Saravi, Chinmoy Sarkar, Jennifer Saulam, Nikolaos Scarmeas, Benedikt Michael Schaarschmidt, Christophe Schinckus, Markus P Schlaich, Jurgen Carlo Schmidt, Art Schuermans, Austin E Schumacher, Falk Schwendicke, Catherine Schwinger, Sadaf G Sepanlou, Mahan Shafie, Hamid R Shahsavari, Masood Ali Shaikh, Husain Shakil, Sunder Sham, Muhammad Aaqib Shamim, Nigussie Tadesse Sharew, Amin Sharifan, Amin Shavandi, Rekha Raghuveer Shenoy, Mahabalesh Shetty, Pavanchand H Shetty, Premalatha K Shetty, Mika Shigematsu, Aminu Shittu, Ivy Shiue, Seyed Afshin Shorofi, Rajan Shrestha, Roman Shrestha, Emmanuel Edwar Siddig, João Pedro Silva, Luís Manuel Lopes Rodrigues Silva, Soraia Silva, Puneetpal Singh, Surjit Singh, Jussi O T Sipilä, Anna Aleksandrovna Skryabina, Anton Sokhan, Soroush Soraneh, Joan B Soriano, Ireneous N Soyiri, Michael Spartalis, Paschalis Steiropoulos, Leo Stockfelt, Jing Sun, Johan Sundström, David Sunkersing, Katharina S Sunnerhagen, Chandan Kumar Swain, Lukasz Szarpak, Sree Sudha T Y, Payam Tabaee Damavandi, Rafael Tabarés-Seisdedos, Seyyed Mohammad Tabatabaei, Celine Tabche, Ramin Tabibi, Jabeen Taiba, Manoj Tanwar, Nathan Y Tat, Nuno Taveira, Mohamad-Hani Temsah, Rasiah Thayakaran, Tenaw Yimer Tiruye, Mathilde Touvier, Marcos Roberto Tovani-Palone, Jasmine T Tran, Ngoc Ha Tran, Thang Huu Tran, Domenico Trico, Samuel Joseph Tromans, Evangelia Eirini Tsermpini, Lorainne Tudor Car, Munkhtuya Tumurkhuu, Saeed Ullah, Brigid Unim, Asokan Govindaraj Vaithinathan, Mario Valenti, Jef Van den Eynde, Orsolya Varga, Tommi Juhani Vasankari, Balachandar Vellingiri, Massimiliano Veroux, Dominique Vervoort, Jorge Hugo Villafañe, Francesco S Violante, Giuseppe Vizzielli, Alice Vodden, Stein Emil Vollset, Theo Vos, Hatem A Wafa, Yanzhong Wang, Emebet Gashaw Wassie, Kosala Gayan Weerakoon, Ronny Westerman, Nuwan Darshana Wickramasinghe, Peter Willeit, Marcin W Wojewodzic, Axel Walter Wolf, Charles D A Wolfe, Grant M A Wyper, Xiaoyue Xu, Yuichi Yasufuku, Sanni Yaya, Saber Yezli, Arzu Yiğit, Dong Keon Yon, Chuanhua Yu, Fathiah Zakham, Aurora Zanghì, Michael Zastrozhin, Mohammed G M Zeariya, Liqun Zhang, Zhiqiang Zhang, Claire Chenwen Zhong, Bin Zhu, Makan Ziafati, Magdalena Zielińska, Elric Zweck, Sa'ed H Zyoud, and John N Newton.[§]

*First author

†Second author

‡Third author

§Senior author

Affiliations

Department of Primary Care and Public Health (Prof N Steel PhD), Norwich Medical School (T Gillam MSc), University of East Anglia, Norwich, UK; Office for Health Improvement and Disparities (C M M Bauer-Staeb PhD, S S Fox MSc, A Hughes MSc), Academic Public Health England (Prof S Rawaf MD), Office for Health Improvement and Disparities (Prof N Steel PhD), Department of Health and Social Care, London, UK; Department of Legal and Economic Studies (C Abbafati PhD), Department of Public Health and Infectious Diseases (M S Cattaruzza PhD), La Sapienza University, Rome, Italy; Hull York Medical School (M A Abdalla PhD), University of Hull, Hull, UK; Department of Mathematics and Sciences (A Abdelkader PhD), Ajman University, Ajman, United Arab Emirates; Department of Medicine (P Abdi BEng), Memorial University, St. John's, NL, Canada; Postgraduate Department (Prof R Abeldaño Zuñiga PhD), University of Sierra Sur, Miahuatlan de Porfirio Diaz, Mexico; Yhteiskuntadatatieteen keskus (Centre for Social Data Science) (Prof R Abeldaño Zuñiga PhD), Department of Public Health (Prof T Lallukka PhD), University of Helsinki, Helsinki, Finland (T J Meretoja MD); Department of Internal Medicine (O O Abiodun FWACP), Federal Medical Centre, Abuja, Nigeria; Research Center for Immunodeficiencies (H Abolhassani PhD), Non-communicable Diseases Research Center (M Bastan MD), Iranian Research Center for HIV/AIDS (IRCHA) (O Dadras PhD), Cardiac Primary Prevention Research Center (S Kazemian MD), Department of Cardiac Electrophysiology (S Kazemian MD), School of Medicine (S Khanmohammadi MD), Sina Trauma and Surgery Research Center (M Khormali MD), NeuroTRACT Association (A Moghadam Fard MD), Digestive Diseases Research Institute (S G Sepanlou MD), Department of Neurology (M Shafie MD), Sina Hospital (A Sharifan PharmD), Tehran University of Medical Sciences, Tehran, Iran; Department of Medical Biochemistry and Biophysics (H Abolhassani PhD), Department of Global Public Health (E E Agardh PhD, Prof P Allebeck MD), Department of Neurobiology, Care Sciences and Society (Prof J Ärnlöv PhD), Department of Neurobiology, Care Sciences, and Society (S Fereshtehnejad PhD), Department of Molecular Medicine and Surgery (Prof J H Kauppila MD), Karolinska Institutet (Karolinska Institute), Stockholm, Sweden; Department of Clinical Sciences (Prof E Abu-Gharbieh PhD), Department of Basic Biomedical Sciences (Prof Y Bustanji PhD), Clinical Sciences Department (Prof R Hamoudi PhD), Department of Finance and Economics (Prof M Hussain PhD), College of Medicine (Prof M A Saleh PhD), University of Sharjah, Sharjah, United Arab Emirates; Department of Biopharmaceutics and Clinical Pharmacy (Prof E Abu-Gharbieh PhD), University of Jordan, Amman, Jordan; Medical Research Center (H J Abukhadijah MPH), Nursing & Midwifery Research Department (NMRD) (A J Nashwan PhD), Hamad Medical Corporation, Doha, Qatar; Department of Biochemistry and Molecular Medicine (A Abu-Zaid PhD), Alfaisal University, Riyadh, Saudi Arabia; College of Graduate Health Sciences (A Abu-Zaid PhD), University of Tennessee, Memphis, TN, USA; School of Medicine (I Y Addo PhD), School of Architecture, Design, and Planning (Prof T Astell-Burt PhD), University of Sydney, Sydney, NSW, Australia (S R Okeke PhD); Centre for Social Research in Health (I Y Addo PhD, S R Okeke PhD), School of Population Health (X Feng PhD, X Xu PhD), University of New South Wales, Sydney, NSW, Australia; Internal Medicine and Alcohol Related Disease Unit (Prof G Addolorato MD), Fondazione Policlinico Universitario A. Gemelli IRCCS, Rome, Italy; Department of Medical and Surgical Sciences (Prof G Addolorato MD), Università Cattolica di Roma (Catholic University of Rome), Rome, Italy; Department of Obstetrics and Gynecology (V Adekanmbi PhD), Department of Family Medicine (W Lee PhD), University of Texas Medical Branch, Galveston, TX, USA; Department of Biochemistry (J B Adetunji PhD), Osun State University, Osogbo, Nigeria; Department of Pharmacology and Therapeutics (T E Adeyeoluwa PhD), Department of Microbiology (O O Bello PhD, T C Ekundayo PhD), University of Medical Sciences, Ondo, Ondo, Nigeria; Department of Veterinary Medicine (T E

Adeyeoluwa PhD), Department of Epidemiology and Medical Statistics (A F Fagbamigbe PhD), Department of Periodontology and Community Dentistry (O F Fagbule FWACS), Department of Veterinary Public Health and Preventive Medicine (O A Ogundijo MSc), Faculty of Public Health (I I Olufadewa MHS), Department of Medicine (Prof M O Owolabi DrM), University of Ibadan, Ibadan, Nigeria; Department of Public Health Sciences (W Agyemang-Duah PhD), Queen's University, Kingston, ON, Canada; School of Medicine and Psychology (D Ahmad PhD), Australian National University, Canberra, ACT, Australia; Public Health Foundation of India, Gandhinagar, India (D Ahmad PhD); Maternal and Child Health Division (A Ahmed MS, S Noor MS), International Centre for Diarrhoeal Disease Research, Bangladesh, Dhaka, Bangladesh; Department of Women's and Children's Health (A Ahmed MS), Department of Surgical Sciences (M Hultström PhD), Department of Medical Cell Biology (M Hultström PhD), Department of Medical Sciences (Prof A O Larsson PhD, D Lindholm MD, Prof J Sundström PhD), Uppsala University, Uppsala, Sweden; Institute of Endemic Diseases (A Ahmed MSc), Unit of Basic Medical Sciences (E E Siddig MD), University of Khartoum, Khartoum, Sudan; Swiss Tropical and Public Health Institute (A Ahmed MSc), University of Basel, Basel, Switzerland; Brody School of Medicine (S Ahmed PhD), Department of Computer Science (A O Bodunrin MSc), East Carolina University, Greenville, NC, USA; Department of Internal Medicine (K Akinosoglou PhD), University of Patras, Patras, Greece; Department of Internal Medicine and Infectious Diseases (K Akinosoglou PhD), University General Hospital of Patras, Patras, Greece; Department of Cardiology (M Akkaif PhD), Fudan University, Shanghai, China; Department of Communicable Diseases (S Al Awaidy MSc), Ministry of Health, Muscat, Oman; Middle East, Eurasia, and Africa Influenza Stakeholders Network, Muscat, Oman (S Al Awaidy MSc); Division of Public Health Sciences (S Al Hasan PhD), Department of Surgery (S Azadnajafabad MD), Washington University in St. Louis, St. Louis, MO, USA; Department of Adult Health and Critical Care, College of Nursing (O A Al Zaabi PhD), Department of Geography (W Ali PhD), Sultan Qaboos University, Muscat, Oman; Institute of Health Informatics (R W Aldridge PhD), Department of Health Informatics (S Chung PhD), Surgical Biotechnology Department (Prof R Hamoudi PhD), Department of Population Health Sciences (D Sunkersing PhD), University College London, London, UK; Institute for Health Metrics and Evaluation (R W Aldridge PhD, S D Glenn MSc, Prof S I Hay FMedSci, Prof H J Larson PhD, Prof C J L Murray DPhil, Prof M Naghavi PhD, A E Schumacher PhD, Prof S E Vollset DrPH, Prof T Vos PhD), Department of Health Metrics Sciences, School of Medicine (R W Aldridge PhD, Prof S I Hay FMedSci, Prof C J L Murray DPhil, Prof M Naghavi PhD, Prof S E Vollset DrPH, Prof T Vos PhD), University of Washington, Seattle, WA, USA; Department of Bacteriology, Immunology, and Mycology (Prof A M Algammal PhD), Suez Canal University, Ismailia, Egypt; Global Centre for Environmental Remediation (A A S Al-Gheethi PhD), University of Newcastle, Newcastle, NSW, Australia; Cooperative Research Centre for Contamination Assessment and Remediation of the Environment, Newcastle, NSW, Australia (A A S Al-Gheethi PhD); Department of Zoology (A Ali PhD), Abdul Wali Khan University Mardan, Mardan, Pakistan; Department of Medical Rehabilitation (Physiotherapy) (M U Ali PhD), University of Maiduguri, Maiduguri, Nigeria; Department of Rehabilitation Sciences (M U Ali PhD), Hong Kong Polytechnic University, Hong Kong, China; Center for Biotechnology and Microbiology (S S Ali PhD), University of Swat, Swat, Pakistan; Department of Pathophysiology and Transplantation (G Alicandro PhD), Department of Food, Environmental and Nutritional Sciences (C Del Bo' PhD), Università degli Studi di Milano (University of Milan), Milan, Italy; Cystic Fibrosis Center (G Alicandro PhD), Fondazione IRCCS Ospedale Maggiore Policlinico (IRCCS "Ca' Granda Maggiore Policlinico" Hospital Foundation), Milan, Italy; Institute of Health and Wellbeing (S M Alif PhD), Federation University Australia, Melbourne, VIC, Australia; School of Public Health and Preventive Medicine (S M Alif PhD), Monash

University, Melbourne, VIC, Australia; School of Physics, Mathematics and Computing (Prof A Al-Jumaily PhD), Dobney Hypertension Centre (Prof M P Schlaich MD), The University of Western Australia, Perth, WA, Australia; Information and Communication Technology Research Pole (Lab-STICC) (Prof A Al-Jumaily PhD), ENSTA Bretagne, Brest, France; Department of Allied Medical Sciences (A Alrawashdeh PhD), Department of Rehabilitation Sciences (M Al-Wardat PhD), Jordan University of Science and Technology, Irbid, Jordan; Institute of Public Health (R H Al-Rifai PhD), College of Medicine and Health Sciences (J Nauman PhD), United Arab Emirates University, Al Ain, United Arab Emirates; Department of Emergency Medicine (M A Alsabri MD), Sana'a University, Sanaa, Yemen; Pediatric Emergency Medicine Department (M A Alsabri MD), St. Christopher's Hospital for Children, Philadelphia, PA, USA; Department of Family and Community Medicine (N Z Alshahrani MD), University of Jeddah, Jeddah, Saudi Arabia; Lisbon Institute of Global Mental Health (D O Aluh MSc), Nova University of Lisbon, Lisbon, Nigeria; Clinical Pharmacy and Pharmacy Management (D O Aluh MSc), University of Nigeria Nsukka, Nsukka, Nigeria; Department of Biomedical Engineering (W A Al-Zyoud PhD), German Jordanian University, Amman, Jordan; Spiritual Health Research Center (S Amiri PhD), Nephrology and Urology Research Center (K Hushmandi PhD), Baqiyatallah University of Medical Sciences, Tehran, Iran; Centre for Sensorimotor Performance (D Anderlini MD), The University of Queensland, Brisbane, QLD, Australia (M Moni PhD); Neurology Department (D Anderlini MD), Royal Brisbane and Women's Hospital, Brisbane, QLD, Australia; Department of Cardiology (Prof C Andrei PhD), Department of Legal Medicine and Bioethics (Prof S Hostiuc PhD), Carol Davila University of Medicine and Pharmacy, Bucharest, Romania; Department of Pharmacology (A Anil MD, A Saravanan MD, M Shamim MBBS, S Singh MD), All India Institute of Medical Sciences, Jodhpur, India; All India Institute of Medical Sciences, Bhubaneswar, India (A Anil MD); Regenerative Medicine, Organ Procurement and Transplantation Multi-disciplinary Center (S Anvari MD), Department of Social Medicine and Epidemiology (A Feizkhah MD), Guilan University of Medical Sciences, Rasht, Iran; Rural Health Research Institute (A E Anyasodor PhD, Prof J Sun PhD), Charles Sturt University, Orange, NSW, Australia; Department of Sociology and Social Work (S Appiah PhD), Kwame Nkrumah University of Science and Technology, Kumasi, Ghana; Center for International Health (S Appiah PhD), Ludwig Maximilians University, Munich, Germany; CyberKnife Center (M Aquilano MD), Istituto Fiorentino Di Cura E Assistenza (IFCA), florence, Italy; Health Management and Economics Research Center (J Arabloo PhD), School of Medicine (M Bastan MD), Department of Ophthalmology (H Hasani MD), Research Center of Pediatric Infectious Diseases (F Khamesipour PhD), Department of Health Economics (M Khosravi PhD), Iran University of Medical Sciences, Tehran, Iran; College of Pharmacy (M Arafat PhD), Al Ain University, Abu Dhabi, United Arab Emirates; College of Art and Science (D Areda PhD), Ottawa University, Surprise, AZ, USA; School of Life Sciences (D Areda PhD), Arizona State University, Tempe, AZ, USA; Department of Veterinary Pharmacology and Toxicology (A Aremu PhD), University of Ilorin, Ilorin, Nigeria; School of Public Health (K Armani PhD, S Basu PhD), Department of Brain Sciences (L D'Anna PhD), National Heart & Lung Institute (Prof D Munblit PhD), WHO Collaborating Centre for Public Health Education and Training (D L Rawaf MD), Department of Primary Care and Public Health (Prof S Rawaf MD, C Tabche MSc, L Tudor Car PhD), The George Institute for Global Health (Prof S Yaya PhD), Imperial College London, London, UK; Faculty of Pharmaceutical Sciences (K Armani PhD), UCSI University, Kuala Lumpur, Malaysia; Department of Cardiovascular, Endocrine-Metabolic Diseases and Aging (B Armocida MD), Istituto Superiore di Sanità (ISS), Rome, Italy; School of Health and Social Studies (Prof J Ärnlöv PhD), Dalarna University, Falun, Sweden; Department of Community Medicine and Global Health (M Asaduzzaman MPH), University of Oslo, Oslo, Norway; Northumbria HealthCare NHS Foundation Trust, Newcastle

upon Tyne, UK (A Aujayeb MBBS); School of Business (Prof M Ausloos PhD), Department of Health Sciences (S J Tromans PhD), University of Leicester, Leicester, UK; Department of Statistics and Econometrics (Prof M Ausloos PhD), Bucharest University of Economic Studies, Bucharest, Romania; Leeds Institute of Rheumatic and Musculoskeletal Medicine (S Azadnajafabad MD), University of Leeds, Leeds, UK; Institute of Biotechnology and Genetic Engineering (S Aziz MS), The University of Agriculture, Peshawar, Pakistan; ASIDE Healthcare, Lewes, DE, USA (A Azzam MD); Faculty of Medicine (A Azzam MD), October 6 University, 6th of October City, Egypt; Department of Population Medicine (Prof G Babu PhD), Qatar University, Doha, Qatar; School of Health Sciences (A C Badache MSc), Orebro University, Orebro, Sweden; Swedish Institute of Disability Research (A C Badache MSc), Örebro University, Örebro, Sweden; Department of Forensic Science (A D Badiye PhD, N Kapoor PhD), Government Institute of Forensic Science Nagpur, Nagpur, India; Rashtrasant Tukadoji Maharaj Nagpur University, Nagpur, India (A D Badiye PhD); School of Medicine (S Bahramian MD), Heart Failure Research Center (S Najdaghi MD, D Narimani Davani MD), Neuroscience Research Center (S Najdaghi MD), Isfahan University of Medical Sciences, Isfahan, Iran; International Medical School (A A Baig PhD), Management and Science University, Alam, Malaysia; Center for Clinical Research and Prevention (J L Baker PhD), Bispebjerg University Hospital, Frederiksberg, Denmark; Department of Forensic Science (H Bansal MSc), Government Institute of Forensic Science, Nagpur, Nagpur, India; Heidelberg Institute of Global Health (HIGH) (Prof T W Bärnighausen MD, Prof S Mohammed PhD), Department of Ophthalmology (S Panda-Jonas MD), Heidelberg University, Heidelberg, Germany; T.H. Chan School of Public Health (Prof T W Bärnighausen MD, E Zweck MD), Center for Primary Care (S Basu PhD), Harvard Business School (F Caetano dos Santos PhD), Department of Global Health and Social Medicine (M Pigeolet MD), Harvard University, Boston, MA, USA; Programs, Partnerships, Research and Education (M T U Barone PhD), International Diabetes Federation, São Paulo, Brazil; International Diabetes Federation, Brussels, Belgium (M T U Barone PhD); Department of Public and Environmental Health (A Barrow MPH), University of The Gambia, Banjul, The Gambia; Department of Epidemiology (A Barrow MPH), Division of Pulmonary, Critical Care, and Sleep (M Beiranvand PhD), College of Medicine (A A Beneke MS, M J Diaz BS, K T Root BS), University of Florida, Gainesville, FL, USA; Heidelberg Institute of Global Health (HIGH) (S Barteit PhD), Heidelberg University Hospital, Heidelberg, Germany; University Institute of Food Science and Technology (S Bashir PhD), The University of Lahore, Lahore, Pakistan; Department of Animal Sciences (H A Bashiru PhD), Obafemi Awolowo University, Ile-Ife, Nigeria; Faculty of Pharmacy (J D Basso PharmD, S Silva MSc), Coimbra Chemistry Centre (J D Basso PharmD), Coimbra Institute for Biomedical Imaging and Translational Research (S Silva MSc), University of Coimbra, Coimbra, Portugal; Department of Medical Education (K Batra PhD), University of Nevada Las Vegas, Las Vegas, NV, USA; Department of Health Sciences (DISSAL) (M Bauckneht PhD), University of Genoa, Genova, Italy; Department of Nuclear Medicine (M Bauckneht PhD), IRCCS Ospedale Policlinico San Martino, Genova, Italy; Department of Psychiatry (Prof B T Baune PhD), University of Münster, Münster, Germany; Department of Psychiatry (Prof B T Baune PhD), Melbourne Medical School, Melbourne, VIC, Australia; Department of Mental Health (M Beghi MD), AUSL Romagna, Ravenna, Italy; Department of Neurology (Prof Y Béjot PhD), University Hospital of Dijon, Dijon, France; Dijon Stroke Registry (Prof Y Béjot PhD), University of Burgundy, Dijon, France; School of the Environment (Prof M L Bell PhD), Department of Dermatology (M Goldust MD), Department of Radiology and Biomedical Imaging (M Mayeli MD), Department of Genetics (S Pawar PhD), Department of Internal Medicine (R Shrestha PhD), Yale University, New Haven, CT, USA; School of Health Policy and Management (Prof M L Bell PhD), Korea University, Seoul, South Korea; Department of Biological Sciences (Prof L Belo PhD), Research Unit on

Applied Molecular Biosciences (UCIBIO) (Prof L Belo PhD, Prof F Carvalho PhD, Prof C Fortuna Rodrigues PhD, J Silva PhD), Associated Laboratory for Green Chemistry (LAQV) (M Carvalho PhD), Department of Chemical Sciences (R A S Couto MD), Institute for Research and Innovation in Health (i3S) (Prof N Cruz-Martins PhD), Faculty of Engineering (Prof C Fortuna Rodrigues PhD), MEDCIDS, Faculty of Medicine of the University of Porto (A Freitas PhD), Epidemiology Research Unit (EPIUnit) (A Ribeiro PhD), University of Porto, Porto, Portugal; Department of Biomedical Sciences (Prof A Beloukas PhD), National AIDS Reference Center of Southern Greece (Prof A Beloukas PhD), University of West Attica, Athens, Greece; Faculty of Medicine (P J G Bettencourt PhD), Universidade Católica Portuguesa (Catholic University of Portugal), Sintra, Portugal; Center for Interdisciplinary Research in Health (CIIS) (P J G Bettencourt PhD), Universidade Católica Portuguesa (Catholic University of Portugal), Lisbon, Portugal; Department of Public Health (A S Bhagavathula PhD), North Dakota State University, Fargo, ND, USA; Division of Gastroenterology and Hepatology (A S Bhagavathula PhD), Mayo Clinic, Jacksonville, FL, USA; Institute of Applied Health Research (N Bhala PhD), School of Medicine - Division of Ophthalmology & Visual Sciences (Prof G D Panos MD), University of Nottingham, Nottingham, UK; Institute of Applied Health Research (N Bhala PhD, K Malhotra MBBS, R Thayakaran PhD), NIHR Global Health Research Unit on Global Surgery (J C Glasbey MSc), University of Birmingham, Birmingham, UK; Global Health Neurology Lab (S Bhaskar MD), NSW Brain Clot Bank, Sydney, NSW, Australia; Division of Cerebrovascular Medicine and Neurology (S Bhaskar MD), National Cerebral and Cardiovascular Center, Suita, Japan; Department of Biomedical and NeuroMotor Sciences (Prof F Bisulli PhD), Department of Medical and Surgical Sciences (Prof R Bugiardini MD, E Cenko MD, Prof F S Violante MD), Dipartimento di Scienze Biomediche e Neuromotorie (DIBINEM) (F Esposito MD), Department of Biomedical and Neuromotor Sciences (S Guicciardi MD, L Muccioli MD), University of Bologna, Bologna, Italy; UOC Clinica Neurologica (Prof F Bisulli PhD), IRCCS Istituto delle Scienze Neurologiche di Bologna (Institute of Neurological Sciences of Bologna), Bologna, Italy; Department of Global Public Health and Primary Care (Prof T Bjørge PhD), Center for International Health (C Schwinger PhD), University of Bergen, Bergen, Norway; Department of Research (M W Wojewodzic PhD), Cancer Registry of Norway, Oslo, Norway (Prof T Bjørge PhD); Facultad de Salud (Faculty of Health) (Prof A Botero Carvajal PhD), Universidad Santiago de Cali (Santiago de Cali University), Cali, Colombia; Department of Medicine (Prof S Bouaoud DrPH), University Ferhat Abbas of Setif, Setif, Algeria; Department of Epidemiology and Preventive Medicine (Prof S Bouaoud DrPH), University Hospital Saadna Abdenour, Setif, Algeria; Department of Psychiatry (Prof C Brayne MD), Department of Public Health and Primary Care (Prof P Willeit PhD), University of Cambridge, Cambridge, UK; Division of Clinical Epidemiology and Aging Research (Prof H Brenner MD), German Cancer Research Center, Heidelberg, Germany; School of Primary Care, Population Sciences and Medical Education (A D M Briggs PhD), School of Psychology (Prof S Cortese PhD), Southampton Clinical Trials Unit (P H Lee PhD), Faculty of Medicine (R Thayakaran PhD), University of Southampton, Southampton, UK; Department of Epidemiology and Evidence-Based Medicine (Prof N I Briko DSc), Department of Paediatrics and Paediatric Infectious Diseases (Prof D Munblit PhD), I.M. Sechenov First Moscow State Medical University, Moscow, Russia; Department of Woman and Child Health and Public Health (D Buonsenso MD), Fondazione Policlinico Universitario A. Gemelli IRCCS (Agostino Gemelli University Polyclinic IRCCS), Rome, Italy; Global Health Research Institute (D Buonsenso MD), Università Cattolica del Sacro Cuore (Catholic University of Sacred Heart), Rome, Italy; Department of Health Care Management (Prof R Busse PhD), Technische Universität Berlin, Berlin, Germany; School of Pharmacy (Prof Y Bustanji PhD), The University of Jordan, Amman, Jordan; Faculty of Health Sciences (M Çakmak Barsbay PhD), Ankara University, Ankara, Turkiye; Department of Health Management (Direzione

Sanitaria) (A Capodici MD), IRCCS Istituto Ortopedico Rizzoli, Bologna, Italy; Interdisciplinary Research Center for Health Science (A Capodici MD), Sant'Anna School of Advanced Studies, Pisa, Italy; Oncological Network, Prevention and Research Institute (G Gorini MD), Institute for Cancer Research, Prevention and Clinical Network, Florence, Italy (G Carreras PhD); Dermatology Unit, Department of Medicine and Surgery (A Carugno MD), University of Insubria, Varese, Italy; PhD Program in Molecular and Translational Medicine (DIMET) (A Carugno MD), University of Milan Bicocca, Milan, Italy; Faculty of Health Sciences (M Carvalho PhD), University Fernando Pessoa, Porto, Portugal; Department of Psychiatry (Prof J Castaldelli-Maia PhD), University of São Paulo, São Paulo, Brazil; Division of Country Health Policies and Systems (CPS) (G Castelpietra PhD), World Health Organisation, -, Italy; Mental Health Flagship (G Castelpietra PhD), World Health Organization (WHO), Copenhagen, Denmark; Department of Pharmacological and Biomolecular Sciences (Prof A L Catapano PhD), Department of Food, Environmental and Nutritional Sciences (I Cioffi PhD, Prof D Martini PhD), Department of Clinical Sciences and Community Health (Prof C La Vecchia MD), University of Milan, Milan, Italy; MultiMedica Sesto San Giovanni IRCCS, Sesto San Giovanni, Italy (Prof A L Catapano PhD); Department of Medical, Surgical, and Health Sciences (L Cegolon PhD, Prof M D'Oria MD), University of Trieste, Trieste, Italy; Public Health Unit (L Cegolon PhD), University Health Agency Giuliano-Isontina (ASUGI), Trieste, Italy; Mary MacKillop Institute for Health Research (Prof E Cerin PhD), Australian Catholic University, Melbourne, VIC, Australia; School of Public Health (Prof E Cerin PhD), Department of Urban Planning and Design (C Sarkar PhD), University of Hong Kong, Hong Kong, China; Institute of Clinical Physiology (S Cerrai MSc), Italian National Council of Research, Pisa, Italy; Department of Biology (A A Chaudhary PhD), Al-Imam Mohammad Ibn Saud Islamic University, Riyadh, Saudi Arabia; Department of Medicine (B Chong MBBS), Saw Swee Hock School of Public Health (S Ramazanu PhD), National University of Singapore, Singapore, Singapore; Department of Community Medicine (Prof S G Choudhari MD), Jawaharlal Nehru Medical College, Wardha, India; The Interdisciplinary Research Group on Biomedicine and Health (D Chu PhD), Faculty of Applied Sciences (D Chu PhD), VNU International School (VNUIS), Hanoi, Vietnam; Department of Paediatric Surgery (I S Chukwu BMedSc), Federal Medical Centre, Umuahia, Nigeria; Health Data Research UK, London, UK (S Chung PhD); Nova Medical School (Prof J Conde PhD), Nova University of Lisbon, Lisbon, Portugal; Department of Child and Adolescent Psychiatry (Prof S Cortese PhD), Department of Population Health (S Jaka MD), New York University, New York, NY, USA; Department of Family Medicine and Public Health (Prof M H Criqui MD), University of California San Diego, La Jolla, CA, USA; Department of Diagnostic and Therapeutic Technologies (Prof N Cruz-Martins PhD), Cooperativa de Ensino Superior Politécnico e Universitário (Polytechnic and University Higher Education Cooperative), Vila Nova de Famalicão, Portugal; Research Center for Child Psychiatry (O Dadras PhD), Heart Center (V Kytö MD), Department of Clinical Neurosciences (J O T Sipilä PhD), University of Turku, Turku, Finland; Centre for Public Health (M T Dallat PhD), Queen's University, Belfast, Northern Ireland; Department of Medical and Surgical Sciences and Advanced Technologies "GF Ingrassia" (Prof E D'Amico MD, A Maugeri PhD, Prof M Veroux PhD), Department of Biomedical and Biotechnological Sciences (L Falzone PhD), Department of General Surgery and Medical-Surgical Specialties (Prof G Isola PhD), University of Catania, Catania, Italy; Department of Public Health (S D Darcho MPH), Haramaya University, Harar, Ethiopia; Department of Clinical Toxicology (Prof P I Dargan FEAPCCT), Guy's and St. Thomas' NHS Foundation Trust, London, UK; Faculty of Life Sciences and Medicine (Prof P I Dargan FEAPCCT), Department of Twin Research and Genetic Epidemiology (M Mazidi PhD), School of Population Health and Environmental Sciences (H A Wafa MPH), School of Life Course and Population Sciences (Prof Y Wang PhD, Prof C D A Wolfe MD), King's College London, London, UK;

Department of Biochemistry (S Das MD), Ministry of Health and Welfare, New Delhi, India; Department of Legal Medicine, Psychiatry and Pathology (A de la Torre-Luque PhD), Universidad Complutense de Madrid (Complutense University of Madrid), Madrid, Spain; Department of Neurosurgery (A K Demetriades MD), University of Edinburgh, Edinburgh, UK; Department of Neurosurgery (A K Demetriades MD), National Health Service (NHS) Scotland, Edinburgh, UK; St Paul's Eye Unit (N Dervenis MD), Royal Liverpool University Hospital, Liverpool, UK; Department of Ophthalmology (N Dervenis MD), 2nd Cardiology Department (P Karakasis MSc), School of Medicine - First Department of Ophthalmology (Prof G D Panos MD), Second Department of Cardiology (Prof D Patoulias PhD), Aristotle University of Thessaloniki, Thessaloniki, Greece; Department of Epidemiology and Public Health (Prof B Devleesschauwer PhD), Sciensano, Brussels, Belgium; Department of Translational Physiology (Prof B Devleesschauwer PhD), Infectiology and Public Health, Ghent, Belgium; Sheffield Teaching Hospitals NHS Foundation Trust, Sheffield, UK (A Dhali MBBS); Division of Pathology (K Dhama PhD), ICAR-Indian Veterinary Research Institute, Bareilly, India; Department of Epidemiology (M Dianatinasab MSc), Sunway University, Selangor, Malaysia; Department of Epidemiology (M Dianatinasab MSc), Care and Public Health Research Institute (CAPHRI) (R Kamath MHA), Maastricht University, Maastricht, Netherlands; Health Science Center (D Dongarwar MS), University of Texas, Houston, TX, USA; Cardio-Thoraco-Vascular Department (Prof M D'Oria MD), Azienda Sanitaria Universitaria Giuliano Isontina, Trieste, Italy; Independent Consultant, South Plainfield, NJ, USA (O P Doshi MS); Department of Epidemiology and Biostatistics (R K Dowou MPhil), Department of Population and Behavioural Sciences (E Manu PhD), University of Health and Allied Sciences, Ho, Ghana; Faculty of Science and Humanities (S Duraisamy PhD), SRM Institute of Science and Technology, Kattankulathur, India; Department of Infection and Tropical Medicine (O C Durojaiye MPH), University of Sheffield, Sheffield, UK; Department of Conservative Dentistry with Endodontics (A M Dziedzic DSc), Medical University of Silesia, Katowice, Poland; School of Nursing and Midwifery (Prof D Edvardsson PhD, Prof M Rahman PhD), La Trobe University, Melbourne, VIC, Australia; School of Nursing and Midwifery (K Edvardsson PhD), La Trobe University, Bundoora, VIC, Australia; Centre for Global Health Inequalities Research (CHAIN) (Prof T Eikemo PhD, H Hoven PhD), Department of Circulation and Medical Imaging (J Nauman PhD), Norwegian University of Science and Technology, Trondheim, Norway; Faculty of Science and Health (M Ekholuenetale PhD), University of Portsmouth, Hampshire, UK; Almoosa College of Health Sciences, Al Ahsa, Saudi Arabia (R A El Arab PhD); School of Population and Global Health (Prof F J Elgar PhD), McGill University, Montreal, QC, Canada; Faculty of Medicine (M Elhadi MD), University of Tripoli, Tripoli, Libya; Houston Methodist Hospital, Houston, TX, USA (M Elhadi MD); Department of Pediatrics (C Eltaha MD), University of Texas, Dallas, TX, USA; Department of Biomedical Sciences (N Fabin MD, M Valenti MD), Humanitas University, Milan, Italy; Dermatology Unit (M Valenti MD), IRCCS Humanitas Research Hospital, Milan, Italy (N Fabin MD); Research Centre for Healthcare and Community (A F Fagbamigbe PhD), Faculty of Health and Life Sciences (O P Kurmi PhD), Coventry University, Coventry, UK; Department of Periodontology and Community Dentistry (O F Fagbule FWACS), Department of Medicine (Prof M O Owolabi DrM), University College Hospital, Ibadan, Ibadan, Nigeria; Department of Food Hygiene and Quality Control (A Fakhri-Demeshghieh DVM), University of Tehran, Tehran, Iran; Epidemiology and Biostatistics Unit (L Falzone PhD), IRCCS Pascale, Naples, Italy; Dissemination Division (C S e Farinha MSc), National Institute of Statistics, Lisbon, Portugal; Activity Planning and Control Unit (C S e Farinha MSc), Directorate-General of Health (DGS), Lisbon, Portugal; Department of Biology (P S Faris PhD), Department of Statistics (Prof D H Kadir PhD), Department of Chemistry (H I M Amin PhD), Salahaddin University-Erbil, Erbil, Iraq; Department of Biology (P S Faris PhD), Department of Business

Administrations (Prof D H Kadir PhD), Department of Medical Biochemical Analysis (H I M Amin PhD), Cihan University-Erbil, Erbil, Iraq; Department of Veterinary Tropical Diseases (Prof F O Fasina PhD), School of Health Systems & Public Health (R E Ogunsakin PhD), University of Pretoria, Pretoria, South Africa; Animal Production and Health Division (EMPRES) (Prof F O Fasina PhD), Food and Agriculture Organization of the United Nations, Rome, Italy; Department of Biology and Medicine (P Fazeli MSc), Brown University, Providence, RI, USA; Laboratory of Experimental Medicine (T Fazylov MD), Research and Publication Activity Division (M Kulimbet MSc), Kazakh National Medical University, Almaty, Kazakhstan; Department of Infectious Diseases and Public Health (G Fekadu PhD), City University of Hong Kong, Hong Kong, China; Department of Pharmacy (G Fekadu PhD), Department of Nursing (G Fetensa MSc), Wollega University, Nekemte, Ethiopia; National Institute of Environmental Health (X Feng PhD), Chinese Center for Disease Control and Prevention, Beijing, China; Division of Neurology (S Fereshtehnejad PhD), Institute of Health Policy, Management, and Evaluation (H Shakil MD), Division of Neurosurgery (H Shakil MD), University of Toronto, Toronto, ON, Canada; Department of Translational Medicine (D Ferrante PhD), University of Piemonte Orientale, Italy, Novara, Italy; Center for Public Health Research (P Ferrara PhD), University of Milan Bicocca, Monza, Italy; Laboratory of Public Health (P Ferrara PhD), IRCCS Istituto Auxologico Italiano, Milan, Italy; Department of Social Sciences (Prof N Ferreira PhD), University of Nicosia, Nicosia, Cyprus; Institute of Public Health (F Fischer PhD), Charité Universitätsmedizin Berlin (Charité Medical University Berlin), Berlin, Germany; Department of Cardiac, Thoracic, Vascular Sciences and Public Health (M Fonzo MD), University of Padova, Padova, Italy; Neurology, Public Health, Disability Unit (A Fornari PhD), Fondazione IRCCS Istituto Neurologico Carlo Besta di Milano, Milano, Italy; Innovation in Healthcare and Social Services Department (D Fortuna MSc), Emilia-Romagna Region, Bologna, Italy; Department of Neuroscience (M Foschi MD), Multiple Sclerosis Research Center, Ravenna, Italy; Department of Biotechnological and Applied Clincial Sciences (M Foschi MD), University of L'Aquila, L'Aquila, Italy; Center for Health Technology and Services Research (CINTESIS), Porto, Portugal (A Freitas PhD); Department of Dermatology (T Fukumoto PhD), Kobe University, Kobe, Japan; Department of Community Medicine (Prof M A Gadanya MD), Bayero University Kano, Kano, Nigeria; Department of Community Medicine (Prof M A Gadanya MD), Aminu Kano Teaching Hospital, Kano, Nigeria; Department of Medical Epidemiology (S Gallus PhD), Laboratory of Neurological Disorders (G Giussani PhD), Department of Environmental Health Sciences (A Lugo PhD), Mario Negri Institute for Pharmacological Research, Milan, Italy; Department of Cardiovascular, Endocrine-metabolic Diseases, and Aging (L Galluzzo MA), ISS - Italian National Institute of Health, Rome, Italy; Institute of Health and Wellbeing (B Ganesan PhD), Federation University Australia, Churchill, VIC, Australia; Department of General Medicine (M Ganiyani MD), Grant Medical College & Sir J.J. Group of Hospitals, Mumbai, India; Department of Medicine (M Ganiyani MD), Department of Medical Oncology (A A Khosla MD), Miami Cancer Institute, Miami, FL, USA; Department of Biostatistics (X Gao PhD), Key Lab of Environment and Health (X Gao PhD), Xuzhou Medical University, Xuzhou, China; Faculty of Business and Management (M Garcia-Gordillo PhD), Universidad Autonóma de Chile (Autonomous University of Chile), Talca, Chile; Independent Consultant, Rome, Italy (F Gazzelloni MSc); Department of Midwifery (M W Gebregergis MSc), Department of Medical Laboratory Sciences (H N Meles MSc), Adigrat University, Adigrat, Ethiopia; Department of Reproductive and Family Health (T G Gebremeskel PhD), Axum College of Health Science, Axum, Ethiopia; College of Medicine and Public Health (T G Gebremeskel PhD, B Kaambwa PhD, G R Naik PhD), Health Economics Unit (B Kaambwa PhD), Department of Nursing and Health Sciences (S Shorofi PhD), Flinders University, Adelaide, SA, Australia; School of Medicine (D J Ghadimi MD), Department of Health (M Safari PhD), Shahid Beheshti University

of Medical Sciences, Tehran, Iran; Department of Public Health (K Y Ghailan PhD), Jazan University, Jazan, Saudi Arabia; Center of Health Management (K Y Ghailan PhD), Aden University, Aden, Yemen; Research Group for Childhood Cancer (N Ghith PhD), Cancer Research Institute, Danish Cancer Society, Copenhagen, Denmark; Department of Electrical and Computer Engineering (E Gholami PhD), University of California Davis, Davis, CA, USA; Department of Epidemiology and Prevention (A Gialluisi PhD), IRCCS Neuromed, Pozzilli, Italy; Warwick Medical School (Prof P S Gill DM, Prof J A B Rodriguez PhD), University of Warwick, Coventry, UK; Department of Cardiac Surgery (L Göbölös PhD), Cleveland Clinic Abu Dhabi, Abu Dhabi, United Arab Emirates; Lerner College of Medicine (L Göbölös PhD), Department of Quantitative Health Science (X Liu PhD), Case Western Reserve University, Cleveland, OH, USA; Department of Health Systems and Policy Research (Prof M Golechha PhD), Indian Institute of Public Health, Gandhinagar, India; Department of Genetics (P Goleij MSc), Sana Institute of Higher Education, Sari, Iran; Universal Scientific Education and Research Network (USERN) (P Goleij MSc), Kermanshah University of Medical Sciences, Kermanshah, Iran; Department of Life Sciences, Health and Healthcare Professions (Prof D Golinelli MD), Link Campus University, Rome, Italy; Health Services Research, Evaluation and Policy Unit (Prof D Golinelli MD), AUSL della Romagna, Ravenna, Italy; Nuffield Department of Orthopaedics, Rheumatology, and Musculoskeletal Sciences (S M Graham PhD), Nuffield Department of Medicine (Prof R J Maude PhD), University of Oxford, Oxford, UK; Liverpool Orthopaedic and Trauma Service (S M Graham PhD), Department of Surgery (Prof R Lunevicius DSc), University of Liverpool, Liverpool, UK; Competence Centre Health Promotion and Healthcare (R Griebler PhD), Austrian National Public Health Institute, Vienna, Austria; Department of Endocrinology (A Grover MD), National Institutes of Health, Bethesda, MD, USA; Department of the Health Directorate (S Guicciardi MD), Local Health Authority of Bologna, Bologna, Italy; Department of Psychiatry (S Gunturu MD), Bronxcare Health System, Bronx, NY, USA; Department of Psychiatry (S Gunturu MD), Icahn School of Medicine at Mount Sinai, New York, NY, USA; School of Biotechnology (V Gupta PhD), Dublin City University, Dublin, Ireland; Department of Biomedical Gerontology (R S Gutiérrez-Murillo PhD), Pontifical Catholic University of Rio Grande do Sul, Porto Alegre, Brazil; Department of Medical Microbiology (A D Habteyohannes PhD), Department of Psychiatry (T B Mossie MSc), College of Medicine and Health Sciences (H B Netsere MSc), Bahir Dar University, Bahir Dar, Ethiopia; Department of Surgery (N Haep MD), Charité Medical University Berlin, Berlin, Germany; Clinician Scientist Program (N Haep MD), Berlin Institute of Health, Berlin, Germany; Department of Liver Tumor, Cancer Center (N Hai Nam PhD), Liver Transplant Unit (N Hai Nam PhD), Cho Ray Hospital, Ho Chi Minh City, Vietnam; Department of Infectious Disease Epidemiology (S Haller MD), Robert Koch Institute, Berlin, Germany; Department of Public Health (S Haller MD), Charité Institute of Public Health, Berlin, Germany; Trends and Analysis Sector (S Handanagic MD), European Monitoring Centre for Drugs and Drug Addiction, Lisbon, Portugal; Research Unit (J M Haro MD), Parc Sanitari Sant Joan de Deu, Barcelona, Spain; Department of Mental Health (J M Haro MD), Carlos III Health Institute (Prof R Tabarés-Seisdedos PhD), Biomedical Research Networking Center for Mental Health Network (CiberSAM), Madrid, Spain; Department of Pharmacy (Prof M S Hasnain PhD), Marwadi University, Rajkot, India; Skaane University Hospital (R J Havmoeller PhD), Skaane County Council, Malmö, Sweden; Faculty of Kinesiology (Prof J J Hebert PhD), University of New Brunswick, Fredericton, NB, Canada; School of Allied Health (Prof J J Hebert PhD), Murdoch University, Murdoch, WA, Australia; Department of Medicine (B Heibati PhD), University of Alberta, Edmonton, AB, Canada; Health and Society (H B M Hilderink DrPH), National Institute for Public Health and the Environment, Bilthoven, Netherlands; Graduate School of Medicine (Y Hiraike PhD), Department of Global Health Policy (S K Rauniyar PhD), University of Tokyo, Tokyo, Japan;

School of Dentistry (N Hoan DDS), Hanoi Medical University, Hanoi, Vietnam; School of Computer Science (Prof M Hosseinzadeh PhD), Duy Tan University, Da Nang, Vietnam; Jadara University Research Center (Prof M Hosseinzadeh PhD), Jadara University, Irbid, Jordan; Department of Clinical Legal Medicine (Prof S Hostiuc PhD), National Institute of Legal Medicine Mina Minovici, Bucharest, Romania; Institute for Occupational and Maritime Medicine (ZfAM) (H Hoven PhD), University Medical Center Hamburg-Eppendorf (UKE), Hamburg, Germany; Department of Psychology (C Hu PhD), Tsinghua University, Beijing, China; Faculty of Medicine (J Huang MD), Jockey Club School of Public Health and Primary Care (C Zhong PhD), The Chinese University of Hong Kong, Hong Kong, China; Department of Biological Sciences and Chemistry (Prof J Hussain PhD), Natural and Medical Sciences Research Center (A Khan PhD), School of Pharmacy (A K Philip PhD), University of Nizwa, Nizwa, Oman; Department of Social Sciences and Business (Prof M Hussain PhD), Roskilde University, Roskilde, Denmark; Collaborative Alliance Research and Education (CARE) Programme (A Ikiroma PhD), Episcope Research Service, Aberdeen, Scotland; Faculty of Health and Life Sciences (A Inok PhD, Prof J N Newton MSc), University of Exeter, Exeter, UK; School of Pharmacy (M Islam PhD), BRAC University, Dhaka, Bangladesh; Institute for Physical Activity and Nutrition (Prof S Islam PhD), Deakin University, Burwood, VIC, Australia; Department of Microbiology (M Iyer PhD), Human Genetics and OMICS, Department of Zoology (B Vellingiri PhD), Central University of Punjab, Bathinda, India; Department of Physical and Medicine (L Jacob MD), Université Paris Cité, Paris, France; Research and Development Unit (L Jacob MD), Biomedical Research Networking Center for Mental Health Network (CiberSAM), Barcelona, Spain (R F Palma-Alvarez PhD); College of Medicine and Medical Sciences (H Jahrami PhD), Arabian Gulf University, Manama, Bahrain; Ministry of Health, Manama, Bahrain (H Jahrami PhD); Department of Health and Safety (A A Jairoun PhD), Dubai Municipality, Dubai, United Arab Emirates; The World Academy of Sciences UNESCO, Trieste, Italy (Prof M Jakovljevic PhD); Shaanxi University of Technology, Hanzhong, China (Prof M Jakovljevic PhD); Department of Pharmacology (T Jawaid PhD), Imam Mohammad Ibn Saud Islamic University, Riyadh, Saudi Arabia; Department of Internal Medicine (B M Jeswani MBBS), GCS Medical College, Hospital & Research Centre, Ahmedabad, India; Rothschild Foundation Hospital (Prof J B Jonas MD), Institut Français de Myopie, Paris, France; Singapore Eye Research Institute (Prof J B Jonas MD), Singapore Eye Research Institute, Singapore, Singapore; Department of Economics (C E Joshua BSc), National Open University, Benin City, Nigeria; School of Public Health (Z Kabir PhD), University College Cork, Cork, Ireland; Prasanna School of Public Health (R Kamath MHA), Department of Pharmacology (R R Shenoy PhD), Manipal Academy of Higher Education, Manipal, India; Faculty of Dentistry (K K Kanmodi MPH), University of Puthisastra, Phnom Penh, Cambodia; Office of the Executive Director (K K Kanmodi MPH), Cephas Health Research Initiative Inc, Ibadan, Nigeria; European Observatory on Health Systems and Policies, London, UK (M Karanikolos PhD); Department of Health Services Research and Policy (M Karanikolos PhD, Prof M McKee DSc), Department of Infectious Disease Epidemiology (Prof H J Larson PhD), London School of Hygiene & Tropical Medicine, London, UK; School of Health Professions and Human Services (I M Karaye MD), Hofstra University, Hempstead, NY, USA; Department of Anesthesiology (I M Karaye MD), Montefiore Medical Center, Bronx, NY, USA; Surgery Research Unit (Prof J H Kauppila MD), Oulu Business School (I Shiue PhD), Martti Ahtisaari Institute (I Shiue PhD), University of Oulu, Oulu, Finland; Department of Human Nutrition (E Kesse-Guyot PhD), National Research Institute for Agriculture, Food and Environment, Jouy-en-Josas, France; Department of Health, Medicine and Human Biology (M Touvier PhD), Sorbonne Paris Nord University, Bobigny, France (E Kesse-Guyot PhD); Halal Research Center of the Islamic Republic of Iran (IRI) (F Khamesipour PhD), Iran Food and Drug Administration, Tehran, Iran; Department of Epidemiology (S

Khanmohammadi MD), Non-Communicable Diseases Research Center (NCDRC), Tehran, Iran; College of Health, Wellbeing and Life Sciences (Prof K Khatab PhD), Sheffield Hallam University, Sheffield, UK; College of Arts and Sciences (Prof K Khatab PhD), Ohio University, Zanesville, OH, USA; Department of Basic Medical Sciences (Prof M M Khatatbeh PhD), Yarmouk University, Irbid, Jordan; Department of Internal Medicine (A A Khosla MD), Corewell Health East William Beaumont University Hospital, Royal Oak, MI, USA; Department of Health Management and Economics (M Khosravi PhD), Qom University of Medical Sciences, Qom, Iran; Research Department (M Khosrowjerdi PhD), Inland Norway University of Applied Sciences, Elverum, Norway; Department of Public Health (J Khubchandani PhD), New Mexico State University, Las Cruces, NM, USA; Graduate School of Public Health (K Kim PhD), Kosin University, Busan, South Korea; Broad Institute of MIT and Harvard, Cambridge, MA, USA (M Kim MD); Cardiovascular Research Center (A Schuermans BSc), Massachusetts General Hospital, Boston, MA, USA (M Kim MD); School of Health Sciences (Prof A Kisa PhD), Kristiania University College, Oslo, Norway; Department of International Health and Sustainable Development (Prof A Kisa PhD), Tulane University, New Orleans, LA, USA; Department of Nursing and Health Promotion (S Kisa PhD), Faculty of Health Sciences (Prof A W Wolf PhD), Oslo Metropolitan University, Oslo, Norway; Centre for Disease Burden (A S Knudsen PhD), Department of Disease Burden (C Schwinger PhD), GBD Collaborating Unit (Prof S E Vollset DrPH), Norwegian Institute of Public Health, Bergen, Norway; Copernicus Institute of Sustainable Development (G Koren PhD), Utrecht University, Utrecht, Netherlands; Department of Mathematics (M Kuddus PhD), Department of Population Science and Human Resource Development (Prof M Rahman DrPH), University of Rajshahi, Rajshahi, Bangladesh; James Cook University, Townsville, QLD, Australia (M Kuddus PhD); Department of Pediatrics (I Kuitunen PhD), Kuopio University Hospital, Kuopio, Finland; Institute of Clinical Medicine (I Kuitunen PhD), University of Eastern Finland, Kuopio, Finland; Center of Medicine and Public Health (M Kulimbet MSc), Asfendiyarov Kazakh National Medical University, Almaty, Kazakhstan; College of Public Health & Health Informatics (R Kumar PhD), Department of Public Health (M G M Zeariya PhD), University of Hail, Hail, Saudi Arabia; Section of Cardiology (Prof S K Kunutsor PhD), University of Manitoba, Winnipeg, MB, Canada; Translational Health Sciences (Prof S K Kunutsor PhD), University of Bristol, Bristol, UK; Department of Medicine (O P Kurmi PhD), Department of Psychiatry and Behavioural Neurosciences (Prof A T Olagunju PhD), McMaster University, Hamilton, ON, Canada; Department of Health Services Research and Management (D Kusuma DSc), City University of London, London, UK; Faculty of Public Health (D Kusuma DSc), University of Indonesia, Depok, Indonesia; Clinical Research Center (V Kytö MD), Turku University Hospital, Turku, Finland; Department of Occupational and Environmental Health (H Lai PhD), Huazhong University of Science and Technology, Wuhan, China; Department of Respiratory and Critical Care Medicine (H Lai PhD), Northern Jiangsu People's Hospital, Yangzhou, China; Department of Health Sciences (DISSAL) (F Lanfranchi MD), University of Genoa, Genoa, Italy; Department of Psychiatry and Psychotherapy (B Langguth PhD), University of Regensburg, Regensburg, Germany; Department of Behavioural Sciences and Learning (Prof A Laplante-Lévesque PhD), Linköping University, Linköping, Sweden; Department of Clinical Chemistry and Pharmacology (Prof A O Larsson PhD), Uppsala University Hospital, Uppsala, Sweden; Department of Medical Science (M Lee PhD), Ajou University School of Medicine, Suwon, South Korea; Department of Precision Medicine (Prof S Lee MD), Sungkyunkwan University, Suwon-si, South Korea; Department of Medicine (D Lindholm MD), Norrtälje Hospital (Tiohundra), Norrtälje, Sweden; UCD Centre for Disability Studies (C Linehan PhD), University College Dublin, Dublin, Ireland; Lerner Research Institute (X Liu PhD), Cleveland Clinic, Cleveland, OH, USA; Department of Molecular Epidemiology (E Llanaj PhD), German Institute of Human Nutrition Potsdam-Rehbrücke, Potsdam, Germany; German

Center for Diabetes Research (DZD), München-Neuherberg, Germany (E Llanaj PhD); One Health Research Group (J López-Gil PhD), Universidad de Las Américas (University of the Americas), Quito, Ecuador; Institute of Nutritional Sciences (Prof S Lorkowski PhD), Friedrich Schiller University Jena, Jena, Germany; Competence Cluster for Nutrition and Cardiovascular Health (nutriCARD), Jena, Germany (Prof S Lorkowski PhD); School of Medicine (Prof G Lucchetti PhD), Federal University of Juiz de Fora, Juiz de Fora, Brazil; Department of Emergency General and Trauma Surgery (Prof R Lunevicius DSc), Liverpool University Hospitals NHS Foundation Trust, Liverpool, UK; Center for Evidence-Based and Translational Medicine (L Luo MPH), Department of Epidemiology and Biostatistics (Prof S Mubarik PhD, Prof C Yu PhD), Wuhan University, Wuhan, China; Centre for Public Health and Wellbeing (Z Ma PhD), University of the West of England, Bristol, UK; 2nd Department of Propaedeutic Surgery (N Machairas PhD), 3rd Department of Cardiology (M Spartalis PhD), University of Athens, Athens, Greece; Department of Periodontology (Prof M Machoy PhD), Pomeranian Medical University, Szczecin, Poland; Rama Medical College Hospital and Research Centre, Uttar Pradesh, India (K Malhotra MBBS); Rabigh Faculty of Medicine (Prof A Malik PhD), King Abdulaziz University, Jeddah, Saudi Arabia; Information and Communication Technology Research Pole (Lab-STICC) (Prof A Mansour PhD), ENSTA, Brest, France; Department of Biomedical Engineering (H Marateb PhD), University of Isfahan, Isfahan, Iran; Biomedical Engineering Research Center (CREB) (H Marateb PhD), Universitat Politècnica de Catalunya (Barcelona Tech - UPC), Barcelona, Barcelona, Spain; Department of Nutrition and Dietetics (M Martorell PhD), Centre for Healthy Living (M Martorell PhD), University of Concepción, Concepción, Chile; Faculty of Humanities and Health Sciences (Prof R R Marzo MD), Curtin University, Sarawak, Malaysia; Jeffrey Cheah School of Medicine and Health Sciences (Prof R R Marzo MD), Monash University, Subang Jaya, Malaysia; Department of Anatomy and Developmental Biology (Y Mathangasinghe PhD), Monash University, Clayton, VIC, Australia; Department of Anatomy, Genetics and Biomedical Informatics (Y Mathangasinghe PhD), Postgraduate Institute of Medicine (S N K Navaratna MD), University of Colombo, Colombo, Sri Lanka; Department of Community Medicine (M Mathur MD), Geetanjali Medical College and Hospital, Udaipur, India; Department of Maternal-Child Nursing and Public Health (Prof F P Matozinhos PhD), Federal University of Minas Gerais, Belo Horizonte, Brazil; Department of Epidemiology (Prof R J Maude PhD), Mahidol Oxford Tropical Medicine Research Unit, Bangkok, Thailand; Department of Infectious Disease Epidemiology (Prof J May MD), Bernhard Nocht Institute for Tropical Medicine, Hamburg, Germany; Department of Tropical Medicine (Prof J May MD), Medical Center Hamburg-Eppendorf (UKE), Hamburg, Germany; Department of Healthcare (Prof E A Mechili PhD), University of Vlora, Vlora City, Albania; Clinic of Social and Family Medicine (Prof E A Mechili PhD), University of Crete, Heraklion, Greece; Department of Medicine, Cedars Sinai Medical Center (S Mehravar MD), University of California Los Angeles, Los Angeles, CA, USA; Department of Public Health (T Mekene Meto MPH), Arba Minch University, Arba Minch, Ethiopia; International Dx Department (A A Mentis MD), BGI Genomics, Copenhagen, Denmark; General Administration Department (A Meretoja MD), Comprehensive Cancer Center (T J Meretoja MD), Department of Neurosurgery (I Rautalin PhD), Helsinki University Hospital, Helsinki, Finland; School of Health Sciences (A Meretoja MD), Melbourne School of Population and Global Health (L Reifels PhD), University of Melbourne, Melbourne, VIC, Australia; Department of Paediatrics (Prof S Mettananda DPhil), University of Kelaniya, Ragama, Sri Lanka; University Paediatrics Unit (Prof S Mettananda DPhil), Colombo North Teaching Hospital, Ragama, Sri Lanka; Anaesthesiology Department (G Micha PhD), "Helena Venizelou" General and Maternity Hospital, Athens, Greece; National Cancer Registry (I Michalek PhD), Department of Pathology (I Michalek PhD), Maria Sklodowska-Curie National Research Institute of Oncology, Warsaw, Poland;

Pacific Institute for Research & Evaluation, Beltsville, MD, USA (T R Miller PhD); School of Public Health (T R Miller PhD), Curtin University, Perth, WA, Australia; Multidisciplinary Department of Medical-Surgical and Dental Specialties (G Minervini PhD), University of Campania Luigi Vanvitelli, Naples, Italy; Saveetha Dental College and Hospitals (G Minervini PhD, M Tovani-Palone PhD), Centre of Molecular Medicine and Diagnostics (COMManD) (Prof S Patil PhD), Saveetha University, Chennai, India; Department of Medical Sciences (A Mirijello MD), IRCCS Casa Sollievo della Sofferenza General Hospital, San Giovanni Rotondo, Italy; Foundation for Liver Research (G Mocciaro PhD), Foundation for Liver Research, London, UK; University of Colorado Anschutz Medical Campus (A Moghadam Fard MD), University of Colorado, Aurora, CO, USA; College of Applied and Natural Science (J Mohamed MSc), University of Hargeisa, Hargeisa, Somalia; Molecular Biology Unit (N S Mohamed MSc), Bio-Statistical and Molecular Biology Department (N S Mohamed MSc), Sirius Training and Research Centre, Khartoum, Sudan; Modeling in Health Research Center (A Mohammadian-Hafshejani PhD), Shahrekord University of Medical Sciences, Shahrekord, Iran; Health Systems and Policy Research Unit (Prof S Mohammed PhD), Ahmadu Bello University, Zaria, Nigeria; Clinical Epidemiology and Public Health Research Unit (L Monasta DSc, L Ronfani PhD), Burlo Garofolo Institute for Maternal and Child Health, Trieste, Italy; Department of Biomedical and Dental Sciences and Morphofunctional Imaging (Prof S Mondello MD), Messina University, Messina, Italy; AI & Cyber Futures Institute (M Moni PhD), Charles Sturt University, Bathurst, NSW, Australia; Computer, Electrical, and Mathematical Sciences and Engineering Division (P Moraga PhD), King Abdullah University of Science and Technology, Thuwal, Saudi Arabia; International Laboratory for Air Quality and Health (Prof L Morawska PhD), Queensland University of Technology, Brisbane, QLD, Australia; Department of Community Medicine (R Motappa MD), Department of Forensic Medicine and Toxicology (Prof J Padubidri MD, P H Shetty MD), Department of Internal Medicine (M M R Reddy MD), Manipal College of Dental Sciences, Mangalore (Prof P K Shetty MDS), Manipal Academy of Higher Education, Mangalore, India; Unit of Pharmacotherapy, Epidemiology and Economics (Prof S Mubarik PhD), University of Groningen (Rijksuniversiteit Groningen), Groningen, Netherlands; Competence Center of Mortality-Follow-Up of the German National Cohort (R Westerman DSc), Federal Institute for Population Research, Wiesbaden, Germany (Prof U O Mueller MD); Center for Population and Health, Wiesbaden, Germany (Prof U O Mueller MD); School of Medicine (F Mughal FRCGP), Keele University, Keele, UK; Division of Psychology and Mental Health (F Mughal FRCGP), University of Manchester, Manchester, UK; Department of Surgery (F Mulita PhD), General University Hospital of Patras, Patras, Greece; Faculty of Medicine (F Mulita PhD), Department of Emergency Medicine (Prof I Pantazopoulos PhD), University of Thessaly, Larissa, Greece; Department of Community and Global Health (Y Munkhsaikhan MD), The University of Tokyo, Tokyo, Japan; Department of Computer Science (P Naghavi MS), University of Illinois, Urbana, IL, USA; Department of Engineering (G R Naik PhD), Western Sydney University, Sydney, NSW, Australia; School of Pharmacy (A Naqvi PhD), University of Reading, Reading, UK; National Dental Research Institute Singapore (G G Nascimento PhD), Duke-NUS Medical School, Singapore, Singapore; Department of Applied Pharmaceutical Sciences and Clinical Pharmacy (A Y Naser PhD), Isra University, Amman, Jordan; Department of Community Medicine (S N K Navaratna MD), University of Peradeniya, Kandy, Sri Lanka; Department of Health Promotion (A Nazri-Panjaki MSc), Zahedan University of Medical Sciences, Zahedan, Iran; Faculty of Medicine (Prof C Nejjari PhD), Euromed University of Fes, Fez, Morocco; Faculty of Medicine (Prof C Nejjari PhD), University Sidi Mohammed Ben Abdellah, Fez, Morocco; Department of Medicine (E Nena MD, P Steiropoulos MD), Democritus University of Thrace, Alexandroupolis, Greece; School of Nursing (H B Netsere MSc), University of Gondar, Gondar, Ethiopia; Department of General Medicine (A H Nguyen MD), Thai Binh

University of Medicine and Pharmacy, Thai Binh City, Vietnam; Department of Surgery (P T Nguyen MD), Danang Family Hospital, Danang, Vietnam; Tuberculosis Group (V T Nguyen MD), Oxford University Clinical Research Unit, Vietnam, Ho Chi Minh City, Vietnam; Department of General Medicine (V T Nguyen MD), Department of Internal Medicine (T H Tran MD), University of Medicine and Pharmacy at Ho Chi Minh City, Ho Chi Minh City, Vietnam; Center for Public Health (L A Nnyanzi PhD), Teesside University, Middlesbrough, UK; Department of Statistics (S Noor MS), Shahjalal University of Science and Technology, Sylhet, Bangladesh; Health Policy Research Center (M Nouri PhD), Non-communicable Disease Research Center (S G Sepanlou MD), Shiraz University of Medical Sciences, Shiraz, Iran; Health Research Institute (M Nouri PhD), School of Medicine (S Soraneh MD), Babol University of Medical Sciences, Babol, Iran; Department of Radiology (F Nugen PhD), Department of Cardiovascular Medicine (H Pham MD), Mayo Clinic, Rochester, MN, USA; School of Information (F Nugen PhD), University of California Berkeley, Berkeley, CA, USA; Department of Health Sciences and Public Health (M Nurchis PhD), School of Economics (M Nurchis PhD), Università Cattolica del Sacro Cuore, Rome, Italy; Department of Physiology (O J Nzoputam PhD), University of Benin, Edo, Nigeria; Department of Physiology (O J Nzoputam PhD), Benson Idahosa University, Benin City, Nigeria; Department of Applied Economics and Quantitative Analysis (Prof B Oancea PhD), University of Bucharest, Bucharest, Romania; Bioinformatics Department (Prof B Oancea PhD), National Institute of Research and Development for Biological Sciences, Bucharest, Romania; Department of Medicine (M J O'Donnell PhD), National University of Ireland - Galway, Galway, Ireland; PSSM Data Sciences (M Oduro PhD), Pfizer Inc., Groton, CT, USA; School of Pharmacy (O C Okonji MSc), University of the Western Cape, Cape Town, South Africa; Department of Psychiatry (Prof A T Olagunju PhD), University of Lagos, Lagos, Nigeria; Department of Literature, Film, and Theatre Studies (Prof S Oliver PhD), University of Essex, Colchester, UK; Slum and Rural Health Initiative Research Academy (I I Olufadewa MHS), Slum and Rural Health Initiative, Ibadan, Nigeria; Department of Medicine (Prof A Ortiz MD), Hospital Universitario de La Princesa (Prof J B Soriano MD), Universidad Autónoma de Madrid (Autonomous University of Madrid), Madrid, Spain; Department of Nephrology and Hypertension (Prof A Ortiz MD), The Institute for Health Research Foundation Jiménez Díaz University Hospital, Madrid, Spain; Department of Respiratory Medicine (Prof M P P A DNB), Jagadguru Sri Shivarathreeswara University, Mysore, India; Department of Mental Health (R F Palma-Alvarez PhD), Hospital Universitari Vall d'Hebron (CIBERSAM), Barcelona, Spain; Centre for Biotechnology (S K Panda PhD), Siksha ‘O’ Anusandhan (Deemed to be University), Bhubaneswar, India; Department of Neurology (L D Panos MD), Department of Emergency Medicine (Prof I Pantazopoulos PhD), University of Bern, Bern, Switzerland; Department of Neurology (L D Panos MD), University of Cyprus, Nicosia, Cyprus; Vision and Eye Research Institute (Prof S Pardhan PhD), Anglia Ruskin University, Cambridge, UK; Department of Epidemiology and Community Health (R R Parikh MD), University of Minnesota, Minneapolis, MN, USA; Department of Medical Sciences (R Passera PhD), University of Torino, Torino, Italy; Department of Imaging (R Passera PhD), AOU Città della Salute e della Scienza di Torino, Torino, Italy; College of Dental Medicine (Prof S Patil PhD), Roseman University of Health Sciences, South Jordan, UT, USA; Department of Biomedical Sciences (U Pensato MD), Humanitas University, Pieve Emanuele (MI), Italy; School of Population Health (Prof G Pereira PhD), Curtin University, Bentley, WA, Australia; Centre for Fertility and Health (Prof G Pereira PhD), Department of Chemical Toxicology (M W Wojewodzic PhD), Norwegian Institute of Public Health, Oslo, Norway; Mario Negri Institute for Pharmacological Research, Bergamo, Italy (N Perico MD, Prof G Remuzzi MD); Department of Food, Environmental and Nutritional Sciences (Prof S Perna PhD), University of Milan, Milano, Italy; Facultad de Medicina (Faculty of Medicine) (F E Petermann-Rocha

PhD), Universidad Diego Portales (Diego Portales University), Santiago, Chile; School of Cardiovascular and Metabolic Health (F E Petermann-Rocha PhD), School of Health and Wellbeing (G M A Wyper MSc), University of Glasgow, Glasgow, UK; Department of Internal Medicine (H Pham MD), University of Arizona, Tucson, AZ, USA; National Centre for Disease Prevention and Health Promotion (D Pierannunzio PhD), National Institute of Health, Roma, Italy; Department of Pediatric Orthopedic Surgery (M Pigeolet MD), Boston Children's Hospital, Boston, MA, USA; Air and Climate Unit (E Pisoni PhD), European Commission, Ispra, Italy; College of Health Sciences (CHS) (Prof D Poddighe PhD), VinUniversity, Hanoi, Vietnam; Clinical Academic Department of Pediatrics (Prof D Poddighe PhD), University Medical Center (UMC), Astana, Kazakhstan; Department of Data Management and Analysis (R Poluru PhD), The International Clinical Epidemiology Network (INCLEN) Trust International, New Delhi, India; University Medical Center Groningen (Prof M J Postma PhD), Interdisciplinary Center Psychopathology and Emotion Regulation (ICPE) (N T Sharew MSc), University of Groningen, Groningen, Netherlands; Center of Excellence in Higher Education for Pharmaceutical Care Innovation (Prof M J Postma PhD), Universitas Padjadjaran (Padjadjaran University), Bandung, Indonesia; Department of Humanities and Social Sciences (Prof J Pradhan PhD), National Institute of Technology Rourkela, Rourkela, India; Department of Neuroscience (E Pupillo PharmD), IRCCS - Istituto di Ricerche Farmacologiche Mario Negri, Milan, Italy; Department of Biostatistics, Epidemiology, and Informatics (J Puvvula PhD), University of Pennsylvania, Philadelphia, PA, USA; UO Neurologia, Salute Pubblica e Disabilità (The Neurology, Public Health and Disability Unit) (A Raggi PhD), Fondazione IRCCS Istituto Neurologico Carlo Besta (IRCCS Foundation Carlo Besta Neurological Institute), Milan, Italy; Institute of Health and Wellbeing (Prof M Rahman PhD), Federation University Australia, Berwick, VIC, Australia; Division of Gynecology and Human Reproduction Physiopathology (D Raimondo PhD), IRCCS Azienda Ospedaliero-Universitaria di Bologna, Bologna, Italy; Department of Medical, Surgical and Experimental Sciences (I Raimondo MD), University of Sassari, Sassari, Italy; Gynecology and Breast Care Center (I Raimondo MD), Mater Olbia Hospital (Qatar Foundation Endowment and Policlinico Universitario Agostino Gemelli IRCCS Foundation), Olbia, Italy; Department of Radiology (S Ramasamy MD), Stanford University, Stanford, CA, USA; School of Nursing & Health Sciences (S Ramazanu PhD), Hong Kong Metropolitan University, Hong Kong, China; Department of Community Medicine (R K Rana MD), Shaheed Nirmal Mahto Medical College and Hospital, Dhanbad, India; Department of Oral Pathology, Microbiology and Forensic Odontology (S Rao MDS), Sharavathi Dental College and Hospital, Shimogga, India; Institute of Collective Health (Prof D Rasella PhD), Federal University of Bahia, Salvador, Brazil; Barcelona Institute for Global Health, Barcelona, Spain (Prof D Rasella PhD); Department of Medicine (A M Rashid MD), Jinnah Sindh Medical University, Karachi, Pakistan; Baylor University, Dallas, TX, USA (A M Rashid MD); The National Institute for Stroke and Applied Neurosciences (I Rautalin PhD), Auckland University of Technology, Auckland, New Zealand; Inovus Medical, St Helens, UK (D L Rawaf MD); Department of Biological Sciences (Prof E M M Redwan PhD), King Abdulaziz University, Jeddah, Egypt; Department of Protein Research (Prof E M M Redwan PhD), Research and Academic Institution, Alexandria, Egypt; Department of Epidemiology and Biostatistics (Prof M Rezaeian PhD), Rafsanjan University of Medical Sciences, Rafsanjan, Iran; University of Southern Denmark, Aarhus, Denmark (A Rijal PhD); Department of Pharmacology and Toxicology (Prof J A B Rodriguez PhD), University of Antioquia, Medellin, Colombia; Maurizio Bufalini Hospital, Cesena, Italy (M Romoli MD); Department of Analytical and Applied Economics (Prof H Rout PhD, C Swain MPhil), RUSA Centre of Excellence in Public Policy and Governance (Prof H Rout PhD), Utkal University, Bhubaneswar, India; Department of Biochemistry and Food Analysis (N Roy PhD), Patuakhali Science and Technology University, Patuakhali, Bangladesh; Department of Cardiology (M

Russo PhD), S. Maria dei Battuti Hospital, Conegliano, Italy; Cardiovascular Department (Prof A M A Saad MD), Zagazig University, Zagazig, Egypt; Department of Medicine (C J Sabet MA), Georgetown University, Washington, DC, USA; Department of Pharmaceuticals (Prof M Sachdeva Dhingra PhD), Bihar College of Pharmacy, Patna, India; Operational Research Center in Healthcare (Prof U Saeed PhD), Near East University (NEU), Nicosia Cyprus, Turkiye; International Center of Medical Sciences Research (ICMSR), Islamabad, Pakistan (Prof U Saeed PhD); Department of Neurology (M Safdarian MD), Christian-Doppler University Hospital, Salzburg, Austria; Spinal Cord Injury and Tissue Regeneration Center Salzburg (SCI-TReCS) (M Safdarian MD), Paracelsus Medical University, Salzburg, Austria; Faculty of Pharmacy (Prof M A Saleh PhD), Mansoura University, Mansoura, Egypt; Technology Management Department (Prof M Z Y Salem PhD), University College of Applied Sciences, Gaza, Palestine; School of Economics and Management (Prof M Z Y Salem PhD), University of Kassel, Kassel, Germany; Department of Global Initiatives (Prof G A Salum PhD), Child Mind Institute, New York, NY, USA; Department of Psychiatry and Legal Medicine (Prof G A Salum PhD), Federal University of Rio Grande do Sul, Porto Alegre, Brazil; Department of Anatomy (Prof V P Samuel PhD), Ras Al Khaimah Medical and Health Sciences University, Ras Al Khaimah, United Arab Emirates; Department of Entomology (A M Samy PhD), Medical Ain Shams Research Institute (MASRI) (A M Samy PhD), Ain Shams University, Cairo, Egypt; Indira Gandhi Medical College and Research Institute, Puducherry, India (A Saravanan MD); Department of Orthopaedics and Trauma Surgery (B Saravi PhD), University of Freiburg, Freiburg, Germany; Department of Orthopaedics (B Saravi PhD), Loretto Hospital Freiburg, Freiburg, Germany; Department of Medical Informatics (J Saulam MSc), Kagawa University, Miki-cho, Japan; Food Processing and Nutrition (J Saulam MSc), Karnataka State Akkamahadevi Women's University, Vijayapura, India; Department of Neurology (Prof N Scarmeas PhD), National and Kapodistrian University of Athens, Athens, Greece; Department of Neurology (Prof N Scarmeas PhD), Columbia University, New York, NY, USA; Department of Diagnostic and Interventional Radiology and Neuroradiology (Prof B M Schaarschmidt MD), University Hospital Essen, Essen, Germany; Faculty of Business and Computing (Prof C Schinckus PhD), University of the Fraser Valley, Abbotsford, BC, Canada; Department of Finance (Prof C Schinckus PhD), International School of Management, Paris, France; Hypertension and Kidney Disease Laboratory (Prof M P Schlaich MD), Baker Heart and Diabetes Institute, Melbourne, VIC, Australia; Chief Data Officer Directorate (J C Schmidt MSc), UK Department of Health and Social Care, London, UK; Department of Cardiovascular Sciences (A Schuermans BSc, J Van den Eynde BSc), Katholieke Universiteit Leuven, Leuven, Belgium; Clinic for Conservative Dentistry and Periodontology (Prof F Schwendicke PhD), University Hospital of the Ludwig-Maximilians-University Munich, Munich, Germany; Department of Chemistry (H Shahsavari PhD), Institute for Advanced Studies in Basic Sciences (IASBS), Zanjan, Iran; Independent Consultant, Karachi, Pakistan (M A Shaikh MD); Department of Pathology and Laboratory Medicine (S Sham MD), Northwell Health, New York, NY, USA; Department of Nursing (N T Sharew MSc), Debre Berhan University, Debre Berhan, Ethiopia; Department for Evidence-based Medicine and Evaluation (A Sharifan PharmD), University for Continuing Education Krems, Krems, Austria; Department of Engineering (A Shavandi PhD), Free University of Brussels, Brussels, Belgium; K S Hegde Medical Academy (Prof M Shetty MD), Nitte University, Mangalore, India; National Institute of Infectious Diseases, Tokyo, Japan (M Shigematsu PhD); Department of Veterinary Public Health and Preventive Medicine (A Shittu MSc), Usmanu Danfodiyo University, Sokoto, Sokoto, Nigeria; Department of Medical-Surgical Nursing (S Shorofi PhD), Mazandaran University of Medical Sciences, Sari, Iran; Department of Public Health (R Shrestha MPH), Nepal Development Society, Pokhara, Nepal; Research Unit for Global Health (R Shrestha MPH), Aarhus University, Aarhus, Denmark; Department of Medical Microbiology and

Infectious Diseases (E E Siddig MD), Erasmus University, Rotterdam, Netherlands; Sport Physical Activity and Health Research & Innovation Center (SPRINT) (Prof L M R Silva PhD), Polytechnic Institute of Guarda, Guarda, Portugal; CICS-UBI Health Sciences Research Center (Prof L M R Silva PhD), University of Beira Interior, Covilhã, Portugal; Department of Human Genetics (P Singh PhD), Punjabi University, Patiala, India; Department of Neurology (J O T Sipilä PhD), North Karelia Central Hospital, Joensuu, Finland; Department of Infectious Diseases and Epidemiology (A A Skryabina MD), Pirogov Russian National Research Medical University, Moscow, Russia; Department of Infectious Diseases (Prof A Sokhan PhD), Kharkiv National Medical University, Kharkiv, Ukraine; Clinical Science Line (Prof A Sokhan PhD), Ludwig Boltzmann Institute of Osteologie, Vienna, Austria; Student Research Committee (S Soraneh MD), Urmia University of Medical Sciences, Urmia, Iran; Centro de Investigación Biomédica en Red Enfermedades Respiratorias (CIBERES) (Center for Biomedical Research in Respiratory Diseases Network), Madrid, Spain (Prof J B Soriano MD); Hull York Medical School (I N Soyiri PhD), University of Hull, Hull City, UK; Occupational and Environmental Medicine Department (L Stockfelt PhD), Institute of Neuroscience and Physiology (Prof K S Sunnerhagen PhD), Institute of Health and Care Sciences (Prof A W Wolf PhD), University of Gothenburg, Gothenburg, Sweden; Institute of Integrated Intelligence and Systems (Prof J Sun PhD), Griffith University, Brisbane, QLD, Australia; Cardiovascular Program (X Xu PhD), The George Institute for Global Health, Sydney, NSW, Australia (Prof J Sundström PhD); Department of Neurocare (Prof K S Sunnerhagen PhD), Sabzevar University of Medical Sciences, Gothenburg, Sweden; Department of Clinical Research and Development (Prof L Szarpak PhD), LUXMED Group, Warsaw, Poland; Collegium Medicul (Prof L Szarpak PhD), John Paul II Catholic University of Lublin, Lublin, Poland; Department of Pharmacology (S T Y MD), All India Institute of Medical Sciences, Deoghar, India; Department of Neurology (P Tabaee Damavandi MD), Neurocenter of Southern Switzerland (NSI), Lugano, Switzerland; Department of Medicine (Prof R Tabarés-Seisdedos PhD), University of Valencia, Valencia, Spain; Department of Medical Informatics (S Tabatabaei PhD), Clinial Research Development Unit (S Tabatabaei PhD), Mashhad University of Medical Sciences, Mashhad, Iran; Department of Health, Safety, and Environmental Management (R Tabibi PhD), Abadan School of Medical Sciences, Abadan, Iran; Department of Environmental, Agricultural and Occupational Health (J Taiba PhD), University of Nebraska Medical Center, Omaha, NE, USA; Sri Ramachandra Medical College and Research Institute, Chennai, India (J Taiba PhD); Department of Radiology (M Tanwar MD), University of Alabama at Birmingham, Birmingham, AL, USA; Taking Our Best Shot, Houston, TX, USA (N Y Tat MS); Department of Research and Innovation (N Y Tat MS), Enventure Medical Innovation, Houston, TX, USA; Egas Moniz School of Health and Science (Prof N Taveira PhD), Egas Moniz Cooperativa de Ensino Superior CRL, Monte da Caparica, Portugal; Faculty of Pharmacy (Prof N Taveira PhD), Universidade de Lisboa (University of Lisbon), Lisbon, Portugal; Pediatric Intensive Care Unit (Prof M Temsah MD), King Saud University, Riyadh, Saudi Arabia; Department of Allied Health and Human Performance (T Y Tiruye PhD), University of South Australia, Adelaide, SA, Australia; Public Health Department (T Y Tiruye PhD), Department of Human Nutrition and Food Sciences (E G Wassie MSc), Debre Markos University, Debre Markos, Ethiopia; Nutritional Epidemiology Research Team (EREN) (M Touvier PhD), National Institute for Health and Medical Research (INSERM), Paris, France; School of Medicine (J T Tran BS), Indiana University, Indianapolis, IN, USA; School of Biomedical Engineering (N Tran MD), University of Technology Sydney, Sydney, NSW, Australia; Department of Business Analytics (T H Tran MD), University of Massachusetts Dartmouth, Dartmouth, MA, USA; Department of Clinical and Experimental Medicine (D Trico MD), University of Pisa, Pisa, Italy; Adult Learning Disability Service (S J Tromans PhD), Leicestershire Partnership National Health Service Trust, Leicester, UK; Department

of Psychiatry (E Tsermpini PhD), Dalhousie University, Halifax, NS, Canada; Department of Internal Medicine (M Tumurkhuu PhD), Wake Forest University, Winston-Salem, NC, USA; International Center for Chemical and Biological Sciences (S Ullah MSc), University of Karachi, Karachi, Pakistan; Department of Cardiovascular, Endocrine-metabolic Diseases and Aging (B Unim PhD), National Institute of Health, Rome, Italy; College of Health and Sport Sciences (A G Vaithinathan MSc), University of Bahrain, Zallaq, Bahrain; Department of Public Health and Epidemiology (O Varga PhD), University of Debrecen, Debrecen, Hungary; UKK Institute, Tampere, Finland (Prof T J Vasankari PhD); Faculty of Medicine and Health Technology (Prof T J Vasankari PhD), Tampere University, Tampere, Finland; Department of Human Genetics & Molecular Biology (B Vellingiri PhD), Bharathiar University, Coimbatore, India; Department of Health Policy and Management (D Vervoort MD), Johns Hopkins University, Baltimore, MD, USA; Department of Physiotherapy (J H Villafañe PhD), Universidad Europea de Madrid (European University of Madrid), Villaviciosa de Odón, Spain; Occupational Medicine Unit (Prof F S Violante MD), Sant'Orsola Malpighi Hospital, Bologna, Italy; Department of Medicine (G Vizzielli PhD), University of Udine, Udine, Italy; Department of Mother Child Health (G Vizzielli PhD), Azienda Sanitaria Universitaria Friuli Centrale, Udine, Italy; Emergency Department (A Vodden MPH), University College London Hospitals, London, UK; Health Equity Evidence Centre (A Vodden MPH), Queen Mary University of London, London, UK (J A Ford PhD); Department of Parasitology (Prof K G Weerakoon PhD), Department of Community Medicine (N D Wickramasinghe MD), Rajarata University of Sri Lanka, Anuradhapura, Sri Lanka; Institute of Clinical Epidemiology, Public Health, Health Economics, Medical Statistics and Informatics (Prof P Willeit PhD), Medical University Innsbruck, Innsbruck, Austria; NIHR Biomedical Research Centre (Prof C D A Wolfe MD), Guy's and St. Thomas' Hospital and Kings College London, London, UK; Department of Biostatistics and Data Science (Y Yasufuku MSc), Osaka University, Suita, Japan; Biostatics, Epidemiology, and Science Computing Department (S Yezli PhD), King Faisal Specialist Hospital & Research Center, Riyadh, Saudi Arabia; Department of Health Management (A Yiğit PhD), Süleyman Demirel Üniversitesi (Süleyman Demirel University), Isparta, Turkiye; Department of Pediatrics (Prof D Yon MD), Kyung Hee University, Seoul, South Korea; Faculty of Medicine and Health Sciences (F Zakham PhD), Hodeidah University, Hodeidah, Yemen; Sant'Elia Hospital (A Zanghì MD), University of Catania, Caltanissetta, Italy; Department of Bioengineering and Therapeutical Sciences (Prof M Zastrozhin PhD), University of California San Francisco, San Francisco, CA, USA; Department of Administration (Prof M Zastrozhin PhD), PGxAI, San Francisco, CA, USA; Department of Zoology and Entomology (M G M Zeariya PhD), Al-Azhar University, Cairo, Egypt; Medical Oncology Department of Gastrointestinal Cancer (L Zhang MS), Cancer Hospital of Dalian University of Technology, Shenyang, China; School of Biomedical Engineering (L Zhang MS), Dalian University of Technology, Dalian, China; Tianjin Medical University General Hospital (Z Zhang MD), Tianjin Centers for Disease Control and Prevention, Tianjin, China; School of Public Health and Emergency Management (B Zhu PhD), Southern University of Science and Technology, Shenzhen, China; Noor Ophthalmology Research Center (M Ziafati MD), Noor Eye Hospital, Tehran, Iran; Department of Biochemistry and Pharmacogenomics (M Zielińska MPharm), Medical University of Warsaw, Warsaw, Poland; Department of Cardiology, Pulmonology, and Vascular Medicine (E Zweck MD), Heinrich-Heine-University, Duesseldorf, Germany; Department of Clinical and Community Pharmacy (Prof S H Zyoud PhD), An-Najah National University, Nablus, Palestine; Clinical Research Centre, An-Najah National University Hospital (Prof S H Zyoud PhD), An-Najah National University Hospital, Nablus, Palestine.

Authors' Contributions

Providing data or critical feedback on data sources

Cristiana Abbafati, Parsa Abdi, Roberto Ariel Abeldaño Zuñiga, Olugbenga Olusola Abiodun, Hassan Abolhassani, Ahmed Abu-Zaid, Victor Adekanmbi, Emilie E Agardh, Danish Ahmad, Ayman Ahmed, Salah Al Awaidy, Robert W Aldridge, Abdelazeem M Algammal, Abid Ali, Syed Shujait Ali, Sheikh Mohammad Alif, Peter Allebeck, Mohammad Al-Wardat, Deanna Anderlini, Saeid Anvari, Seth Christopher Yaw Appiah, Michele Aquilano, Jalal Arabloo, Keivan Armani, Benedetta Armocida, Johan Ärnlöv, Marcel Ausloos, Ahmed Y Azzam, Ashish D Badiye, Atif Amin Baig, Till Winfried Bärnighausen, Amadou Barrow, Shahid Bashir, Mohammad-Mahdi Bastan, Sanjay Basu, Michelle L Bell, Olorunjuwon Omolaja Bello, Apostolos Beloukas, Alice A Beneke, Akshaya Srikanth Bhagavathula, Neeraj Bhala, Sonu Bhaskar, Aadam Olalekan Bodunrin, Souad Bouaoud, Carol Brayne, Danilo Buonsenso, Florentino Luciano Caetano dos Santos, Mehtap Çakmak Barsbay, Joao Mauricio Castaldelli-Maia, Bryan Chong, Dinh-Toi Chu, Rosa A S Couto, Natalia Cruz-Martins, Lucio D'Anna, Samuel Demissie Darcho, Saswati Das, Alejandro de la Torre-Luque, Andreas K Demetriades, Nikolaos Dervenis, Arkadeep Dhali, Mostafa Dianatinasab, Michael J Diaz, Ojas Prakashbhai Doshi, Robert Kokou Dowou, Senbagam Duraisamy, Michael Ekholuenetale, Temitope Cyrus Ekundayo, Rabie Adel El Arab, Chadi Eltaha, Adeniyi Francis Fagbamigbe, Luca Falzone, Carla Sofia e Sá Farinha, Timur Fazylov, Alireza Feizkhah, Ginenus Fekadu, Seyed-Mohammad Fereshtehnejad, Celia Fortuna Rodrigues, Takeshi Fukumoto, Muktar A Gadanya, Silvano Gallus, Balasankar Ganesan, Mohammad Arfat Ganiyani, Federica Gazzelloni, Teferi Gebru Gebremeskel, Ehsan Gholami, James C Glasbey, Laszlo Göbölös, Mahaveer Golechha, Pouya Goleij, Sasidhar Gunturu, Vijai Kumar Gupta, Awoke Derbie Habteyohannes, Nils Haep, Senad Handanagic, Josep Maria Haro, Simon I Hay, Jeffrey J Hebert, Nguyen Quoc Hoan, Mehdi Hosseinzadeh, Chengxi Hu, Andrew Hughes, Michael Hultström, Javid Hussain, Sheikh Mohammed Shariful Islam, Gaetano Isola, Mahalaxmi Iyer, Haitham Jahrami, Mihajlo Jakovljevic, Bijay Mukesh Jeswani, Jost B Jonas, Charity Ehimwenma Joshua, Billingsley Kaambwa, Zubair Kabir, Dler H Hussein Kadir, Neeti Kapoor, Faham Khamesipour, Ajmal Khan, Khaled Khatab, Moawiah Mohammad Khatatbeh, Atulya Aman Khosla, Majid Khosravi, Jagdish Khubchandani, Adnan Kisa, Sezer Kisa, Gerbrand Koren, Ilari Kuitunen, Dian Kusuma, Ville Kytö, Anders O Larsson, Munjae Lee, Seung Won Lee, Xuefeng Liu, Erand Llanaj, Stefan Lorkowski, Zheng Feei Ma, Monika Machoy, Kashish Malhotra, Daniela Martini, Roy Rillera Marzo, Medha Mathur, Andrea Maugeri, Enkeleint A Mechili, Tesfahun Mekene Meto, Atte Meretoja, Tuomo J Meretoja, Sachith Mettananda, Irmina Maria Michalek, Nouh Saad Mohamed, Abdollah Mohammadian-Hafshejani, Shafiu Mohammed, Lorenzo Monasta, Mohammad Ali Moni, Rohith Motappa, Sumaira Mubarik, Ulrich Otto Mueller, Faraz Mughal, Francesk Mulita, Christopher J L Murray, Ganesh R Naik, Abdallah Y Naser, Henok Biresaw Netsere, Anh Hoang Nguyen, Phat Tuan Nguyen, Van Thanh Nguyen, Lawrence Achilles Nnyanzi, Syed Toukir Ahmed Noor, Fred Nugen, Ogochukwu Janet Nzoputam, Bogdan Oancea, Michael Safo Oduro, Oluwaseun Adeolu Ogundijo, Osaretin Christabel Okonji, Andrew T Olagunju, Alberto Ortiz, Mayowa O Owolabi, Mahesh Padukudru P A, Jagadish Rao Padubidri, Sujogya Kumar Panda, Songhomitra Panda-Jonas, Shahina Pardhan, Romil R Parikh, Shankargouda Patil, Shrikant Pawar, Gavin Pereira, Simone Perna, Hoang Nhat Pham, Daniela Pierannunzio, Ramesh Poluru, Maarten J Postma, Jalandhar Pradhan, Jagadeesh Puvvula, Diego Raimondo, Shakthi Kumaran Ramasamy, Sowmya J Rao, Ahmed Mustafa Rashid, Santosh Kumar Rauniyar, Ilari Rautalin, Salman Rawaf, Elrashdy M Moustafa Mohamed Redwan, Ana Isabel Ribeiro, Jefferson Antonio Buendia Rodriguez, Luca Ronfani, Kevin T Root, Michele Russo, Aly M A Saad, Cameron John Sabet, Mamta Sachdeva Dhingra, Umar Saeed, Mehdi Safari, Mahdi Safdarian, Giovanni A Salum, Vijaya Paul Samuel, Abdallah M Samy, Babak Saravi, Chinmoy Sarkar, Art Schuermans,

Austin E Schumacher, Masood Ali Shaikh, Husain Shakil, Sunder Sham, Muhammad Aaqib Shamim, Amin Sharifan, Aminu Shittu, Roman Shrestha, Luís Manuel Lopes Rodrigues Silva, Anna Aleksandrovna Skryabina, Ireneous N Soyiri, Michael Spartalis, Nicholas Steel, Johan Sundström, David Sunkersing, Chandan Kumar Swain, Lukasz Szarpak, Sree Sudha T Y, Rafael Tabarés-Seisdedos, Seyyed Mohammad Tabatabaei, Jabeen Taiba, Mathilde Touvier, Marcos Roberto Tovani-Palone, Jasmine T Tran, Domenico Trico, Munkhtuya Tumurkhuu, Jef Van den Eynde, Tommi Juhani Vasankari, Balachandar Vellingiri, Theo Vos, Kosala Gayan Weerakoon, Ronny Westerman, Charles D A Wolfe, Grant M A Wyper, Sanni Yaya, Chuanhua Yu, Michael Zastrozhin, and Sa'ed H Zyoud.

Developing methods or computational machinery

Cristiana Abbafati, Olugbenga Olusola Abiodun, Robert W Aldridge, Abdelazeem M Algammal, Najim Z Alshahrani, Walid A Al-Zyoud, Ahmed Y Azzam, Giridhara Rathnaiah Babu, Amadou Barrow, Shahid Bashir, Mohammad-Mahdi Bastan, Akshaya Srikanth Bhagavathula, Aadam Olalekan Bodunrin, Souad Bouaoud, Mehtap Çakmak Barsbay, Rosa A S Couto, Lucio D'Anna, Mostafa Dianatinasab, Michael Ekholuenetale, Adeniyi Francis Fagbamigbe, Ehsan Gholami, Simon I Hay, Mehdi Hosseinzadeh, Chengxi Hu, Gaetano Isola, Haitham Jahrami, Bijay Mukesh Jeswani, Charity Ehimwenma Joshua, Dler H Hussein Kadir, Khaled Khatab, Atulya Aman Khosla, Majid Khosravi, Adnan Kisa, Erand Llanaj, Atousa Moghadam Fard, Abdollah Mohammadian-Hafshejani, Mohammad Ali Moni, Francesk Mulita, Christopher J L Murray, Anh Hoang Nguyen, Phat Tuan Nguyen, Van Thanh Nguyen, Mehran Nouri, Ropo Ebenezer Ogunsakin, Shakthi Kumaran Ramasamy, Jefferson Antonio Buendia Rodriguez, Umar Saeed, Abdallah M Samy, Austin E Schumacher, Seyed Afshin Shorofi, Michael Spartalis, Nicholas Steel, Chandan Kumar Swain, Sree Sudha T Y, Ngoc Ha Tran, Jorge Hugo Villafañe, Ronny Westerman, Grant M A Wyper, Michael Zastrozhin, and Mohammed G M Zeariya.

Providing critical feedback on methods or results

Cristiana Abbafati, Mohammed Altigani Abdalla, Atef Abdelkader, Parsa Abdi, Roberto Ariel Abeldaño Zuñiga, Olugbenga Olusola Abiodun, Hassan Abolhassani, Eman Abu-Gharbieh, Hana J Abukhadijah, Ahmed Abu-Zaid, Isaac Yeboah Addo, Victor Adekanmbi, Temitayo Esther Adeyeoluwa, Emilie E Agardh, Williams Agyemang-Duah, Danish Ahmad, Anisuddin Ahmed, Ayman Ahmed, Syed Anees Ahmed, Karolina Akinosoglou, Salah Al Awaidy, Syed Mahfuz Al Hasan, Omar Ali Mohammed Al Zaabi, Robert W Aldridge, Abdelazeem M Algammal, Adel Ali Saeed Al-Gheethi, Abid Ali, Mohammed Usman Ali, Syed Shujait Ali, Waad Ali, Gianfranco Alicandro, Sheikh Mohammad Alif, Adel Al-Jumaily, Peter Allebeck, Mohammed A Alsabri, Najim Z Alshahrani, Mohammad Al-Wardat, Walid A Al-Zyoud, Sohrab Amiri, Deanna Anderlini, Catalina Liliana Andrei, Saeid Anvari, Anayochukwu Edward Anyasodor, Michele Aquilano, Jalal Arabloo, Mosab Arafat, Demelash Areda, Benedetta Armocida, Muhammad Asaduzzaman, Thomas Astell-Burt, Avinash Aujayeb, Marcel Ausloos, Sina Azadnajafabad, Shahkaar Aziz, Ahmed Y Azzam, Giridhara Rathnaiah Babu, Andreea Corina Badache, Ashish D Badiye, Saeed Bahramian, Atif Amin Baig, Jennifer L Baker, Hansi Bansal, Till Winfried Bärnighausen, Mark Thomaz Ugliara Barone, Amadou Barrow, Sandra Barteit, Shahid Bashir, Hameed Akande Bashiru, João Diogo Basso, Mohammad-Mahdi Bastan, Sanjay Basu, Kavita Batra, Matteo Bauckneht, Bernhard T Baune, Clarissa Bauer-Staeb, Yannick Béjot, Michelle L Bell, Olorunjuwon Omolaja Bello, Apostolos Beloukas, Alice A Beneke, Paulo J G Bettencourt, Akshaya Srikanth Bhagavathula, Neeraj Bhala, Sonu Bhaskar, Francesca Bisulli, Aadam Olalekan Bodunrin, Alejandro Botero Carvajal, Souad Bouaoud, Hermann Brenner, Raffaele Bugiardini, Danilo Buonsenso, Reinhard Busse, Yasser Bustanji, Florentino Luciano Caetano dos Santos, Mehtap Çakmak Barsbay, Angelo Capodici, Márcia Carvalho, Joao Mauricio

Castaldelli-Maia, Giulio Castelpietra, Luca Cegolon, Edina Cenko, Ester Cerin, Bryan Chong, Sonali Gajanan Choudhari, Dinh-Toi Chu, Isaac Sunday Chukwu, Sheng-Chia Chung, Joao Conde, Samuele Cortese, Rosa A S Couto, Michael H Criqui, Natalia Cruz-Martins, Omid Dadras, Lucio D'Anna, Samuel Demissie Darcho, Saswati Das, Alejandro de la Torre-Luque, Andreas K Demetriades, Nikolaos Dervenis, Arkadeep Dhali, Kuldeep Dhama, Mostafa Dianatinasab, Michael J Diaz, Deepa Dongarwar, Mario D'Oria, Ojas Prakashbhai Doshi, Robert Kokou Dowou, Senbagam Duraisamy, Oyewole Christopher Durojaiye, Arkadiusz Marian Dziedzic, David Edvardsson, Kristina Edvardsson, Terje Andreas Eikemo, Michael Ekholuenetale, Temitope Cyrus Ekundayo, Rabie Adel El Arab, Frank J Elgar, Muhammed Elhadi, Chadi Eltaha, Natalia Fabin, Adeniyi Francis Fagbamigbe, Omotayo Francis Fagbule, Aliasghar Fakhri-Demeshghieh, Luca Falzone, Carla Sofia e Sá Farinha, Pawan Sirwan Faris, Patrick Fazeli, Alireza Feizkhah, Ginenus Fekadu, Xiaoqi Feng, Seyed-Mohammad Fereshtehnejad, Daniela Ferrante, Pietro Ferrara, Getahun Fetensa, Florian Fischer, Arianna Fornari, Celia Fortuna Rodrigues, Matteo Foschi, Alberto Freitas, Takeshi Fukumoto, Muktar A Gadanya, Lucia Galluzzo, Balasankar Ganesan, Xiang Gao, MA Garcia-Gordillo, Federica Gazzelloni, Miglas Welay Gebregergis, Teferi Gebru Gebremeskel, Delaram J Ghadimi, Khalid Yaser Ghailan, Nermin Ghith, Ehsan Gholami, Paramjit Singh Gill, Tara Gillam, Giorgia Giussani, James C Glasbey, Laszlo Göbölös, Mahaveer Golechha, Davide Golinelli, Simon Matthew Graham, Ashna Grover, Stefano Guicciardi, Sasidhar Gunturu, Vijai Kumar Gupta, Roberth Steven Gutiérrez-Murillo, Awoke Derbie Habteyohannes, Nils Haep, Nguyen Hai Nam, Sebastian Haller, Rifat Hamoudi, Senad Handanagic, Hamidreza Hasani, Md Saquib Hasnain, Rasmus J Havmoeller, Simon I Hay, Jeffrey J Hebert, Behzad Heibati, Yuta Hiraike, Nguyen Quoc Hoan, Mehdi Hosseinzadeh, Hanno Hoven, Chengxi Hu, Michael Hultström, Kiavash Hushmandi, Javid Hussain, M Azhar Hussain, Arit Inok, Md Rabiul Islam, Sheikh Mohammed Shariful Islam, Gaetano Isola, Mahalaxmi Iyer, Louis Jacob, Haitham Jahrami, Ammar Abdulrahman Jairoun, Sanobar Jaka, Mihajlo Jakovljevic, Bijay Mukesh Jeswani, Jost B Jonas, Charity Ehimwenma Joshua, Billingsley Kaambwa, Zubair Kabir, Dler H Hussein Kadir, Rajesh Kamath, Kehinde Kazeem Kanmodi, Neeti Kapoor, Paschalis Karakasis, Marina Karanikolos, Ibraheem M Karaye, Joonas H Kauppila, Sina Kazemian, Faham Khamesipour, Shaghayegh Khanmohammadi, Khaled Khatab, Moawiah Mohammad Khatatbeh, Moein Khormali, Atulya Aman Khosla, Majid Khosravi, Jagdish Khubchandani, Kwanghyun Kim, Min Seo Kim, Adnan Kisa, Sezer Kisa, Ann Kristin Skrindo Knudsen, Gerbrand Koren, Md Abdul Kuddus, Ilari Kuitunen, Setor K Kunutsor, Om P Kurmi, Dian Kusuma, Ville Kytö, Carlo La Vecchia, Hanpeng Lai, Tea Lallukka, Francesco Lanfranchi, Munjae Lee, Seung Won Lee, Wei-Chen Lee, Daniel Lindholm, Xuefeng Liu, Erand Llanaj, José Francisco López-Gil, Stefan Lorkowski, Giancarlo Lucchetti, Lisha Luo, Zheng Feei Ma, Nikolaos Machairas, Monika Machoy, Kashish Malhotra, Ahmad Azam Malik, Ali Mansour, Emmanuel Manu, Hamid Reza Marateb, Daniela Martini, Miquel Martorell, Roy Rillera Marzo, Yasith Mathangasinghe, Medha Mathur, Fernanda Penido Matozinhos, Richard James Maude, Andrea Maugeri, Mahsa Mayeli, Mohsen Mazidi, Martin McKee, Enkeleint A Mechili, Sepideh Mehravar, Tesfahun Mekene Meto, Hadush Negash Meles, Alexios-Fotios A Mentis, Atte Meretoja, Tuomo J Meretoja, Sachith Mettananda, Georgia Micha, Irmina Maria Michalek, Ted R Miller, Giuseppe Minervini, Atousa Moghadam Fard, Jama Mohamed, Nouh Saad Mohamed, Shafiu Mohammed, Stefania Mondello, Mohammad Ali Moni, Paula Moraga, Lidia Morawska, Tilahun Belete Mossie, Rohith Motappa, Sumaira Mubarik, Lorenzo Muccioli, Ulrich Otto Mueller, Faraz Mughal, Francesk Mulita, Yanjinlkham Munkhsaikhan, Christopher J L Murray, Pirouz Naghavi, Ganesh R Naik, Soroush Najdaghi, Atta Abbas Naqvi, Delaram Narimani Davani, Abdulqadir J Nashwan, Javaid Nauman, Athare Nazri-Panjaki, Chakib Nejjari, Evangelia Nena, Henok Biresaw Netsere, John N Newton, Anh Hoang Nguyen, Phat Tuan Nguyen, Van Thanh Nguyen, Lawrence Achilles Nnyanzi, Syed Toukir Ahmed

Noor, Mehran Nouri, Fred Nugen, Mario Cesare Nurchis, Ogochukwu Janet Nzoputam, Bogdan Oancea, Martin James O'Donnell, Michael Safo Oduro, Oluwaseun Adeolu Ogundijo, Osaretin Christabel Okonji, Andrew T Olagunju, Isaac Iyinoluwa Olufadewa, Mayowa O Owolabi, Mahesh Padukudru P A, Jagadish Rao Padubidri, Sujogya Kumar Panda, Songhomitra Panda-Jonas, Georgios D Panos, Leonidas D Panos, Ioannis Pantazopoulos, Shahina Pardhan, Romil R Parikh, Roberto Passera, Shankargouda Patil, Dimitrios Patoulias, Shrikant Pawar, Gavin Pereira, Simone Perna, Fanny Emily Petermann-Rocha, Hoang Nhat Pham, Anil K Philip, Daniela Pierannunzio, Manon Pigeolet, Enrico Pisoni, Ramesh Poluru, Maarten J Postma, Jalandhar Pradhan, Jagadeesh Puvvula, Alberto Raggi, Mosiur Rahman, Muhammad Aziz Rahman, Diego Raimondo, Ivano Raimondo, Shakthi Kumaran Ramasamy, Sheena Ramazanu, Rishabh Kumar Rana, Sowmya J Rao, Ahmed Mustafa Rashid, Santosh Kumar Rauniyar, Ilari Rautalin, David Laith Rawaf, Salman Rawaf, Murali Mohan Rama Krishna Reddy, Elrashdy M Moustafa Mohamed Redwan, Lennart Reifels, Mohsen Rezaeian, Ana Isabel Ribeiro, Anupa Rijal, Jefferson Antonio Buendia Rodriguez, Kevin T Root, Himanshu Sekhar Rout, Michele Russo, Aly M A Saad, Cameron John Sabet, Mamta Sachdeva Dhingra, Umar Saeed, Mehdi Safari, Mahdi Safdarian, Mohamed A Saleh, Giovanni A Salum, Vijaya Paul Samuel, Abdallah M Samy, Babak Saravi, Chinmoy Sarkar, Jennifer Saulam, Nikolaos Scarmeas, Christophe Schinckus, Markus P Schlaich, Art Schuermans, Austin E Schumacher, Falk Schwendicke, Sadaf G Sepanlou, Mahan Shafie, Hamid R Shahsavari, Masood Ali Shaikh, Husain Shakil, Muhammad Aaqib Shamim, Nigussie Tadesse Sharew, Amin Sharifan, Amin Shavandi, Rekha Raghuveer Shenoy, Mika Shigematsu, Aminu Shittu, Ivy Shiue, Seyed Afshin Shorofi, Rajan Shrestha, Roman Shrestha, Emmanuel Edwar Siddig, João Pedro Silva, Luís Manuel Lopes Rodrigues Silva, Soraia Silva, Puneetpal Singh, Anna Aleksandrovna Skryabina, Soroush Soraneh, Joan B Soriano, Ireneous N Soyiri, Michael Spartalis, Nicholas Steel, Leo Stockfelt, Jing Sun, David Sunkersing, Katharina S Sunnerhagen, Chandan Kumar Swain, Lukasz Szarpak, Sree Sudha T Y, Payam Tabaee Damavandi, Rafael Tabarés-Seisdedos, Seyyed Mohammad Tabatabaei, Celine Tabche, Ramin Tabibi, Jabeen Taiba, Manoj Tanwar, Nuno Taveira, Mohamad-Hani Temsah, Rasiah Thayakaran, Tenaw Yimer Tiruye, Mathilde Touvier, Marcos Roberto Tovani-Palone, Jasmine T Tran, Ngoc Ha Tran, Domenico Trico, Samuel Joseph Tromans, Lorainne Tudor Car, Munkhtuya Tumurkhuu, Saeed Ullah, Mario Valenti, Jef Van den Eynde, Orsolya Varga, Balachandar Vellingiri, Massimiliano Veroux, Dominique Vervoort, Jorge Hugo Villafañe, Francesco S Violante, Giuseppe Vizzielli, Alice Vodden, Stein Emil Vollset, Theo Vos, Hatem A Wafa, Yanzhong Wang, Kosala Gayan Weerakoon, Ronny Westerman, Nuwan Darshana Wickramasinghe, Peter Willeit, Marcin W Wojewodzic, Axel Walter Wolf, Charles D A Wolfe, Grant M A Wyper, Xiaoyue Xu, Yuichi Yasufuku, Sanni Yaya, Saber Yezli, Arzu Yiğit, Dong Keon Yon, Chuanhua Yu, Fathiah Zakham, Michael Zastrozhin, Mohammed G M Zeariya, Liqun Zhang, Zhiqiang Zhang, Claire Chenwen Zhong, Bin Zhu, Makan Ziafati, Magdalena Zielińska, Elric Zweck, and Sa'ed H Zyoud.

Drafting the work or revising it critically for important intellectual content

Cristiana Abbafati, Atef Abdelkader, Parsa Abdi, Roberto Ariel Abeldaño Zuñiga, Olugbenga Olusola Abiodun, Hassan Abolhassani, Eman Abu-Gharbieh, Hana J Abukhadijah, Ahmed Abu-Zaid, Isaac Yeboah Addo, Giovanni Addolorato, Victor Adekanmbi, Juliana Bunmi Adetunji, Emilie E Agardh, Danish Ahmad, Anisuddin Ahmed, Ayman Ahmed, Syed Anees Ahmed, Mohammed Ahmed Akkaif, Omar Ali Mohammed Al Zaabi, Robert W Aldridge, Abdelazeem M Algammal, Abid Ali, Mohammed Usman Ali, Syed Shujait Ali, Waad Ali, Gianfranco Alicandro, Peter Allebeck, Ahmad Alrawashdeh, Rami H Al-Rifai, Najim Z Alshahrani, Deborah Oyine Aluh, Mohammad Al-Wardat, Walid A Al-Zyoud, Sohrab Amiri, Deanna Anderlini, Abhishek Anil, Saeid Anvari, Anayochukwu Edward Anyasodor, Seth Christopher Yaw Appiah, Michele Aquilano, Jalal Arabloo, Abdulfatai Aremu, Benedetta Armocida, Johan Ärnlöv,

Muhammad Asaduzzaman, Avinash Aujayeb, Marcel Ausloos, Sina Azadnajafabad, Shahkaar Aziz, Ahmed Y Azzam, Giridhara Rathnaiah Babu, Andreea Corina Badache, Ashish D Badiye, Atif Amin Baig, Hansi Bansal, Till Winfried Bärnighausen, Mark Thomaz Ugliara Barone, Amadou Barrow, Sandra Barteit, Shahid Bashir, Hameed Akande Bashiru, João Diogo Basso, Mohammad-Mahdi Bastan, Sanjay Basu, Matteo Bauckneht, Bernhard T Baune, Clarissa Bauer-Staeb, Massimiliano Beghi, Maryam Beiranvand, Yannick Béjot, Michelle L Bell, Olorunjuwon Omolaja Bello, Luis Belo, Apostolos Beloukas, Paulo J G Bettencourt, Akshaya Srikanth Bhagavathula, Neeraj Bhala, Sonu Bhaskar, Tone Bjørge, Alejandro Botero Carvajal, Souad Bouaoud, Nikolay Ivanovich Briko, Raffaele Bugiardini, Danilo Buonsenso, Yasser Bustanji, Florentino Luciano Caetano dos Santos, Mehtap Çakmak Barsbay, Angelo Capodici, Giulia Carreras, Andrea Carugno, Felix Carvalho, Márcia Carvalho, Joao Mauricio Castaldelli-Maia, Giulio Castelpietra, Alberico L Catapano, Maria Sofia Cattaruzza, Luca Cegolon, Edina Cenko, Ester Cerin, Sonia Cerrai, Anis Ahmad Chaudhary, Bryan Chong, Dinh-Toi Chu, Iolanda Cioffi, Joao Conde, Samuele Cortese, Rosa A S Couto, Natalia Cruz-Martins, Emanuele D'Amico, Lucio D'Anna, Samuel Demissie Darcho, Paul I Dargan, Cristian Del Bo', Andreas K Demetriades, Nikolaos Dervenis, Arkadeep Dhali, Mostafa Dianatinasab, Michael J Diaz, Deepa Dongarwar, Mario D'Oria, Ojas Prakashbhai Doshi, Robert Kokou Dowou, Senbagam Duraisamy, Oyewole Christopher Durojaiye, Arkadiusz Marian Dziedzic, David Edvardsson, Terje Andreas Eikemo, Michael Ekholuenetale, Rabie Adel El Arab, Frank J Elgar, Muhammed Elhadi, Chadi Eltaha, Francesco Esposito, Natalia Fabin, Adeniyi Francis Fagbamigbe, Omotayo Francis Fagbule, Aliasghar Fakhri-Demeshghieh, Luca Falzone, Folorunso Oludayo Fasina, Seyed-Mohammad Fereshtehnejad, Pietro Ferrara, Nuno Ferreira, Getahun Fetensa, Florian Fischer, Marco Fonzo, Arianna Fornari, Daniela Fortuna, Celia Fortuna Rodrigues, Matteo Foschi, Alberto Freitas, Takeshi Fukumoto, Muktar A Gadanya, Silvano Gallus, Lucia Galluzzo, Balasankar Ganesan, Mohammad Arfat Ganiyani, Xiang Gao, MA Garcia-Gordillo, Miglas Welay Gebregergis, Delaram J Ghadimi, Nermin Ghith, Ehsan Gholami, Alessandro Gialluisi, Paramjit Singh Gill, James C Glasbey, Laszlo Göbölös, Mohamad Goldust, Davide Golinelli, Giuseppe Gorini, Ashna Grover, Stefano Guicciardi, Sasidhar Gunturu, Roberth Steven Gutiérrez-Murillo, Nils Haep, Nguyen Hai Nam, Rifat Hamoudi, Josep Maria Haro, Hamidreza Hasani, Md Saquib Hasnain, Rasmus J Havmoeller, Simon I Hay, Jeffrey J Hebert, Yuta Hiraike, Nguyen Quoc Hoan, Sorin Hostiuc, Hanno Hoven, Junjie Huang, Michael Hultström, M Azhar Hussain, Adalia Ikiroma, Arit Inok, Md Rabiul Islam, Sheikh Mohammed Shariful Islam, Gaetano Isola, Mahalaxmi Iyer, Louis Jacob, Haitham Jahrami, Sanobar Jaka, Mihajlo Jakovljevic, Talha Jawaid, Bijay Mukesh Jeswani, Jost B Jonas, Charity Ehimwenma Joshua, Kehinde Kazeem Kanmodi, Neeti Kapoor, Paschalis Karakasis, Marina Karanikolos, Joonas H Kauppila, Sina Kazemian, Emmanuelle Kesse-Guyot, Ajmal Khan, Shaghayegh Khanmohammadi, Khaled Khatab, Moawiah Mohammad Khatatbeh, Atulya Aman Khosla, Majid Khosravi, Mahmood Khosrowjerdi, Jagdish Khubchandani, Kwanghyun Kim, Min Seo Kim, Adnan Kisa, Sezer Kisa, Ann Kristin Skrindo Knudsen, Ilari Kuitunen, Mukhtar Kulimbet, Rakesh Kumar, Setor K Kunutsor, Om P Kurmi, Dian Kusuma, Ville Kytö, Carlo La Vecchia, Hanpeng Lai, Tea Lallukka, Francesco Lanfranchi, Berthold Langguth, Ariane Laplante-Lévesque, Heidi Jane Larson, Anders O Larsson, Paul H Lee, Seung Won Lee, Daniel Lindholm, Christine Linehan, Erand Llanaj, José Francisco López-Gil, Stefan Lorkowski, Giancarlo Lucchetti, Alessandra Lugo, Raimundas Lunevicius, Hawraz Ibrahim M Amin, Zheng Feei Ma, Nikolaos Machairas, Monika Machoy, Kashish Malhotra, Ahmad Azam Malik, Emmanuel Manu, Hamid Reza Marateb, Daniela Martini, Miquel Martorell, Roy Rillera Marzo, Yasith Mathangasinghe, Medha Mathur, Fernanda Penido Matozinhos, Andrea Maugeri, Juergen May, Mahsa Mayeli, Mohsen Mazidi, Enkeleint A Mechili, Hadush Negash Meles, Alexios-Fotios A Mentis, Atte Meretoja, Tuomo J Meretoja, Sachith Mettananda, Georgia Micha, Irmina Maria Michalek, Ted R Miller,

Giuseppe Minervini, Antonio Mirijello, Gabriele Mocciaro, Atousa Moghadam Fard, Nouh Saad Mohamed, Abdollah Mohammadian-Hafshejani, Shafiu Mohammed, Lorenzo Monasta, Stefania Mondello, Mohammad Ali Moni, Paula Moraga, Tilahun Belete Mossie, Rohith Motappa, Ulrich Otto Mueller, Faraz Mughal, Francesk Mulita, Daniel Munblit, Yanjinlkham Munkhsaikhan, Christopher J L Murray, Soroush Najdaghi, Delaram Narimani Davani, Gustavo G Nascimento, Abdallah Y Naser, Abdulqadir J Nashwan, Javaid Nauman, Samidi Nirasha Kumari Navaratna, Henok Biresaw Netsere, John N Newton, Phat Tuan Nguyen, Van Thanh Nguyen, Lawrence Achilles Nnyanzi, Mehran Nouri, Fred Nugen, Mario Cesare Nurchis, Ogochukwu Janet Nzoputam, Bogdan Oancea, Martin James O'Donnell, Oluwaseun Adeolu Ogundijo, Sylvester Reuben Okeke, Osaretin Christabel Okonji, Andrew T Olagunju, Susan Oliver, Alberto Ortiz, Mayowa O Owolabi, Mahesh Padukudru P A, Jagadish Rao Padubidri, Raul Felipe Felipe Palma-Alvarez, Sujogya Kumar Panda, Songhomitra Panda-Jonas, Leonidas D Panos, Ioannis Pantazopoulos, Shahina Pardhan, Romil R Parikh, Roberto Passera, Shankargouda Patil, Dimitrios Patoulias, Shrikant Pawar, Umberto Pensato, Gavin Pereira, Norberto Perico, Simone Perna, Fanny Emily Petermann-Rocha, Hoang Nhat Pham, Daniela Pierannunzio, Dimitri Poddighe, Ramesh Poluru, Maarten J Postma, Jalandhar Pradhan, Elisabetta Pupillo, Jagadeesh Puvvula, Alberto Raggi, Diego Raimondo, Ivano Raimondo, Shakthi Kumaran Ramasamy, Rishabh Kumar Rana, Sowmya J Rao, Davide Rasella, Ahmed Mustafa Rashid, Ilari Rautalin, David Laith Rawaf, Salman Rawaf, Elrashdy M Moustafa Mohamed Redwan, Giuseppe Remuzzi, Ana Isabel Ribeiro, Jefferson Antonio Buendia Rodriguez, Michele Romoli, Luca Ronfani, Kevin T Root, Nitai Roy, Michele Russo, Aly M A Saad, Cameron John Sabet, Mamta Sachdeva Dhingra, Umar Saeed, Mehdi Safari, Mohammed Z Y Salem, Vijaya Paul Samuel, Abdallah M Samy, Aswini Saravanan, Babak Saravi, Chinmoy Sarkar, Nikolaos Scarmeas, Benedikt Michael Schaarschmidt, Christophe Schinckus, Markus P Schlaich, Art Schuermans, Catherine Schwinger, Sadaf G Sepanlou, Mahan Shafie, Husain Shakil, Muhammad Aaqib Shamim, Amin Sharifan, Rekha Raghuveer Shenoy, Mahabalesh Shetty, Pavanchand H Shetty, Premalatha K Shetty, Mika Shigematsu, Aminu Shittu, Seyed Afshin Shorofi, Roman Shrestha, Emmanuel Edwar Siddig, João Pedro Silva, Luís Manuel Lopes Rodrigues Silva, Soraia Silva, Puneetpal Singh, Surjit Singh, Jussi O T Sipilä, Anna Aleksandrovna Skryabina, Anton Sokhan, Joan B Soriano, Ireneous N Soyiri, Michael Spartalis, Nicholas Steel, Paschalis Steiropoulos, Leo Stockfelt, Johan Sundström, David Sunkersing, Katharina S Sunnerhagen, Chandan Kumar Swain, Lukasz Szarpak, Sree Sudha T Y, Payam Tabaee Damavandi, Rafael Tabarés-Seisdedos, Celine Tabche, Jabeen Taiba, Manoj Tanwar, Nathan Y Tat, Nuno Taveira, Mohamad-Hani Temsah, Tenaw Yimer Tiruye, Mathilde Touvier, Marcos Roberto Tovani-Palone, Jasmine T Tran, Thang Huu Tran, Domenico Trico, Samuel Joseph Tromans, Evangelia Eirini Tsermpini, Brigid Unim, Asokan Govindaraj Vaithinathan, Mario Valenti, Jef Van den Eynde, Tommi Juhani Vasankari, Balachandar Vellingiri, Massimiliano Veroux, Dominique Vervoort, Giuseppe Vizzielli, Stein Emil Vollset, Theo Vos, Hatem A Wafa, Yanzhong Wang, Emebet Gashaw Wassie, Kosala Gayan Weerakoon, Ronny Westerman, Nuwan Darshana Wickramasinghe, Peter Willeit, Axel Walter Wolf, Grant M A Wyper, Yuichi Yasufuku, Sanni Yaya, Saber Yezli, Arzu Yiğit, Dong Keon Yon, Aurora Zanghì, Michael Zastrozhin, Mohammed G M Zeariya, Zhiqiang Zhang, Claire Chenwen Zhong, Bin Zhu, Makan Ziafati, Magdalena Zielińska, and Sa'ed H Zyoud.

Managing the estimation or publications process

Simon I Hay, Christopher J L Murray, and Nicholas Steel.
